# Video Recording of Patient-Clinician Interactions in Health Education: Scoping Review

**DOI:** 10.2196/70324

**Published:** 2026-07-13

**Authors:** Mariana Morgado, Yago Leira, Tiago Leitão, José João Mendes, Luís Proença

**Affiliations:** 1 Egas Moniz Center for Interdisciplinary Research (CiiEM), Egas Moniz School of Health & Science, Campus Universitário s/n, Quinta da Granja, Almada, 2829-511, Portugal, 351 212946800; 2 Periodontology Unit, Faculty of Medicine and Odontology, Universidade de Santiago de Compostela, Santiago de Compostela, Spain

**Keywords:** video recordings, patient-clinician interaction, medical education, clinical skills, clinician communication, communication skills, health professions education

## Abstract

**Background:**

Video recordings of patient-clinician interactions (PCIs) have become an integral tool in health professions education, with authentic opportunities to enhance clinical communication and decision-making skills. Despite their educational value, ethical and logistical challenges regarding their use remain underexplored.

**Objective:**

This scoping review aims to map the use of video-recorded PCIs in health education, categorizing types and educational purposes, examining applications across disciplines, and identifying ethical and logistical issues.

**Methods:**

Comprehensive searches were conducted in MEDLINE, Embase, and ERIC (Education Resources Information Center) databases, supplemented by gray literature, with the last search performed on October 30, 2024. Two independent reviewers screened and extracted data on study design, intervention characteristics, educational objectives, and barriers to implementation. Given the scoping review nature, risk of bias assessment was not conducted. Data synthesis involved descriptive analysis and thematic categorization of findings.

**Results:**

A total of 69 studies met inclusion criteria, predominantly from medical (n=28, 41%), dental (n=15, 22%), and nursing (n=8, 12%) education. Publication frequency increased over the past decade. Studies used varied video types, including authentic consultations, simulated encounters, and procedural recordings. Main educational aims focused on clinical skills, communication, professionalism, and reflective practice. Ethical reporting was inconsistent, with variable adherence to informed consent, patient privacy, and confidentiality standards. Common logistical barriers included limitations in technical infrastructure, data management, and curriculum integration.

**Conclusions:**

Video-recorded PCIs offer valuable educational resources but face challenges in ethical standardization and logistical feasibility. Future research should prioritize the development of ethical frameworks and standardized protocols, together with the evaluation of long-term educational and psychological outcomes, to support the responsible and scalable integration of PCI recordings into health education curricula.

## Introduction

### Rationale

Video-based learning is increasingly recognized as a transformative approach in health professions education, supported by several key educational theories. According to constructivist theory, learners actively build new knowledge by integrating multimedia content with prior experience, a process that is facilitated by the contextual richness of video recordings [1,2]. Cognitive load theory also supports video-based learning, demonstrating that well-designed video materials optimize cognitive processing, reduce extraneous mental effort, and enhance comprehension and retention through dual-channel presentation (visual and auditory) [3-5]. Experiential learning theory emphasizes the value of authentic, concrete experiences, such as those provided by video-recorded patient-clinician interactions (PCIs), which enable learners to observe, reflect, conceptualize, and experiment in a cycle that deepens practical understanding [6-8]. Social learning theory highlights the importance of observation and modeling, with video enabling learners to witness expert behaviors and communication strategies in realistic settings [9,10]. Finally, active learning principles are supported by using video as a springboard for discussion, reflection, and collaborative analysis, all of which are linked to improved learner engagement and knowledge retention [11,12].

Since the 1960s, there has been a significant evolution in the integration of video recordings into health education, initially used to assess and improve the technical and interpersonal skills of junior doctors [13]. Technological advances have broadened the scope of video applications, from procedural demonstrations to the recording of real or simulated PCIs [14]. These interactions now serve as valuable resources for developing clinical, communication, and reflective skills [15]. Studies have shown that watching video content before practical exercises improves performance, reduces anxiety, and enhances attitudes, particularly in fields such as nursing and medicine [16-18].

Despite the educational benefits of video recording in clinical education, its adoption raises important ethical and logistical challenges [19,20]. Critical to responsible implementation are ensuring informed consent, maintaining confidentiality, and adhering to legal frameworks such as HIPAA (Health Insurance Portability and Accountability Act) [21,22]. Logistical barriers, including technical setup, scheduling, and resource allocation, also impact the feasibility of integrating video into curricula [21-23]. While most patients and clinicians recognize the educational value of video recording, concerns about privacy and increased scrutiny persist [22,23].

In light of the increasing prevalence and complexity of video-based approaches, there is an evident requirement for a systematic mapping of the characteristics, educational outcomes, and practical challenges associated with the video recording of PCIs in health education.

### Objectives

This scoping review aims to systematically map the use of video recordings of PCIs in health education. More specifically, the review will examine the types and educational purposes of these recordings, compare their effectiveness with that of traditional teaching methods, evaluate their impact on learner outcomes such as clinical competence and communication skills, and identify key ethical and logistical considerations relating to their implementation.

## Methods

### Study Design

We conducted a scoping review in accordance with the Joanna Briggs Institute Reviewer Manual [24,25]. The scoping review is reported according to the PRISMA-ScR (Preferred Reporting Items for Systematic Reviews and Meta-Analyses Extension for Scoping Reviews) checklist [26] (Checklist 1). The final protocol was registered on the Open Science Framework [27] on October 31, 2024.

### Eligibility Criteria

The eligibility criteria for this scoping review were established a priori in order to comprehensively address the research questions. Studies were included if they assessed the usefulness of video recordings of PCIs as an educational tool in health education. These criteria focused on the types, characteristics, and educational outcomes of these video recordings, as well as the ethical and logistical considerations involved in their use. In order to ensure the relevance of the findings to educational contexts, studies were required to include learners in health education, including medical, nursing, dental, and allied health students of all educational levels, who used video recordings of PCIs as part of their training. The video recordings could include full-length consultations, specific clinical procedures, or role-playing scenarios designed to replicate real-world clinical interactions. Other forms of educational media, such as written case studies or noninteractive video content, were excluded to maintain the focus on interactive and video-based learning. All comparators were eligible for inclusion, encompassing studies that compared video-based learning with traditional teaching methods (eg, lectures or textbooks) or with different video formats (eg, live encounters vs simulated scenarios). Noncomparative studies were also considered, providing a comprehensive understanding of the impact of video-based learning. To capture a wide range of evidence, all types of publications were considered, including peer-reviewed articles, conference proceedings, editorials, and book chapters, and no restrictions were placed on the time period or educational setting (eg, universities, colleges, and vocational training programs) to allow for an exploration of trends and developments in the use of video recordings over time. Conversely, studies that did not incorporate learners in health education or that did not use video recordings as a teaching instrument were excluded in order to maintain the focus on the educational application of video-based learning. Studies published in languages other than English were excluded to ensure consistency in data extraction and analysis. Furthermore, unpublished studies and non–peer-reviewed literature were excluded to ensure the credibility and reliability of the findings.

### Information Sources

A comprehensive literature search was conducted in MEDLINE, Embase, and ERIC (Education Resources Information Center) to identify potentially relevant studies on the use of video recordings of PCIs in health education. The final date for the retrieval of articles was October 30, 2024, thereby ensuring that the information included in this review is current.

In addition to the electronic database searches, a comprehensive gray literature search was conducted in accordance with the CADTH (Canadian Agency for Drugs and Technologies in Health) Grey Matters checklist (Checklist 2). This entailed an investigation of a range of gray literature sources and relevant websites with a view to identifying unpublished or difficult-to-locate studies related to video-based learning. Furthermore, the reference lists of pertinent reviews were examined to ascertain any additional studies that may have been overlooked in the database searches. This approach facilitated a more comprehensive collection of information, encompassing both published literature and challenging-to-locate documents.

The final search results were exported into Zotero (Digital Scholar), a reference management software. The presence of duplicates was identified through the use of Zotero’s automated duplicate detection function, which uses a comparison of citation fields such as title, authors, publication year, and journal. A manual review was conducted when necessary in order to ensure that all duplicates were accurately identified and excluded prior to the screening process. This multifaceted search strategy was designed to ensure a comprehensive understanding of the existing literature on the use of video as an educational tool in health education.

### Search Strategy

To facilitate replication, the comprehensive search strategy used, including specific database terms, filters, and a systematic approach to the gray literature, is described below. The primary search strategy, conducted entirely in MEDLINE, is presented in Multimedia Appendix 1, with parallel structures applied to additional databases including Embase and ERIC. This search strategy was developed by one researcher, peer-reviewed by a more experienced member of the research team, and finalized on October 30, 2024.

### Selection of Sources of Evidence

The selection of sources for this scoping review was designed to enable a comprehensive mapping of the use and impact of video recordings of PCIs in health education. To ensure consistency, a calibration exercise was initially conducted. This involved all reviewers independently screening a set of 20 publications with the aim of ensuring a consistent interpretation of the inclusion criteria. This process revealed ambiguities, which were subsequently addressed through group discussions and resulted in the formulation of specific amendments to the screening and data extraction manual.

To facilitate the selection process and ensure consistency, a bespoke screening form was created in Google Sheets. The form included specific questions aligned with the PICO (population, intervention, comparison, and outcomes) framework, which were designed to elicit information regarding the type of video recording (eg, consultations, clinical procedures, and role-playing scenarios), its educational purpose (eg, teaching clinical or communication skills), and how it compares with traditional teaching methods. The form was then trialed by multiple reviewers using sample citations and full-text articles, enabling the questions and layout to be refined to ensure optimal clarity and ease of use. The use of Google Sheets facilitated data management and the efficient tracking of selection decisions.

In the formal screening phase, each title, abstract, and full-text publication identified in the search was evaluated independently by two reviewers (MM and TL), thereby ensuring unbiased study selection. The implementation of an independent duplicate screening process served to minimize the risk of relevant studies being omitted. Disagreements between the reviewers were resolved through consensus discussions. In the event of persistent disagreement, a third reviewer was consulted to make the final decision. This structured and standardized approach guaranteed that our scoping review was conducted systematically, thereby yielding a reliable set of sources for the evaluation of the role of video recordings in enhancing educational outcomes in health education.

### Data Charting Process

A structured data charting form was developed for the purpose of data extraction, with the objective of capturing detailed information from each included source. The charting form was designed with the specific intention of collecting data that aligned with the review’s PICO framework and objectives. It included headers such as screening status, author, year, origin, type of article, study design, aims/purpose, study population, area of study, sample size, methods, intervention type, duration, comparator, outcome measures, and key findings that relate to research questions. The structured approach enabled the systematic capture of data pertaining to the characteristics of the studies, the details of the interventions, and the outcomes.

The initial charting form was developed by two reviewers in collaboration, with items selected based on their relevance to the study objectives. The form was trialed on a sample set of studies, with each reviewer independently charting the data and discussing the results to identify areas requiring elucidation. As a result of this iterative pilot testing, minor adjustments were made to the form in order to ensure clarity and comprehensive coverage. All modifications were duly documented.

In the phase of comprehensive data charting, each study was independently charted by two reviewers using Google Sheets. This approach ensured the organized management of data and enabled the tracking of updates in real time. Any discrepancies in the charting process were resolved through consensus-based discussions. In the event of an unresolved issue, a third reviewer was consulted to provide a final decision. In instances where essential data required clarification, the study investigators were directly contacted.

The structured approach to both selection and data charting guaranteed a comprehensive and reliable dataset, which constituted a robust basis for the analysis of the role of video recordings in health education. Furthermore, it permitted an in-depth examination of the impact of these tools on the educational outcomes of health learners.

### Data Items

The following key characteristics were abstracted from the included articles: author and year of publication, origin of the study, type of article, research design, and data type. Furthermore, additional variables were included, such as the aims and purpose of the research, the characteristics of the student population (eg, year of study, study cycle, and area of study), and details of the intervention (eg, type, duration, and comparator). Additionally, data were collected on the sample size, outcome variables, and instruments used to measure these outcomes. The data items were designed to capture both qualitative and quantitative information, where applicable. For instance, outcome variables and instruments required reviewer judgment based on study reporting. The complete data chart is available in Multimedia Appendix 2, and the detailed definitions of each data item are provided in Multimedia Appendix 3.

### Synthesis of Results

To synthesize the range of evidence included in the scoping review, the studies were grouped based on the type of intervention, which included PCIs, simulated PCIs, and other forms of intervention. For each group, key characteristics were summarized, including the types of settings, populations, and study designs, along with the measures used and the broad findings. Moreover, the studies were classified according to the comparator groups, which included the following: no intervention/control group, traditional methods, simulated PCIs, PCIs, static video recordings of simulated PCIs, self-guided learning, and various other methods.

Studies were also categorized according to the research design and data type used. The research designs included quasi-experimental, observational, descriptive, randomized controlled trial, phenomenological, experimental, comparative, case study, cross-sectional, and nonexperimental. With regard to the data types, the studies were classified as follows: those using a mixed methods approach, those using quantitative methods, and those using qualitative methods.

In addition, the studies were classified according to article type, comprising original research papers and review articles, including literature reviews, scoping reviews, and systematic reviews. Moreover, the studies were classified according to the population degree. The distribution included the following categories: undergraduate, postgraduate, undergraduate and postgraduate, master’s, not applicable, not stated, undergraduate and master’s, and doctorate.

The evidence was further synthesized by categorizing it into key areas of study, including medical education, dental education, nursing education, interprofessional education in health care, occupational therapy education, speech-language therapy education, veterinary medical education, pharmacy education, nutrition and dietetics education, and physiotherapy education.

We synthesized the collated data by using descriptive statistics (frequencies and proportions). We used Microsoft Excel and SPSS software (version 26.0; IBM Corp) to analyze the data.

The results were presented in both narrative form and tabular format. The former provided an overview of the studies within each category and subcategory, while the latter highlighted the general outcome variables and instruments used. Moreover, visual representations, such as diagrams, were included to facilitate the illustration of the distribution of studies across the various categories.

The full synthesis and comprehensive categorization of all evidence are presented in tabular and visual form, facilitating a more nuanced comprehension of the trends, gaps, and overarching findings derived from the reviewed studies.

## Results

### Selection of Sources of Evidence

**Figure 1.**
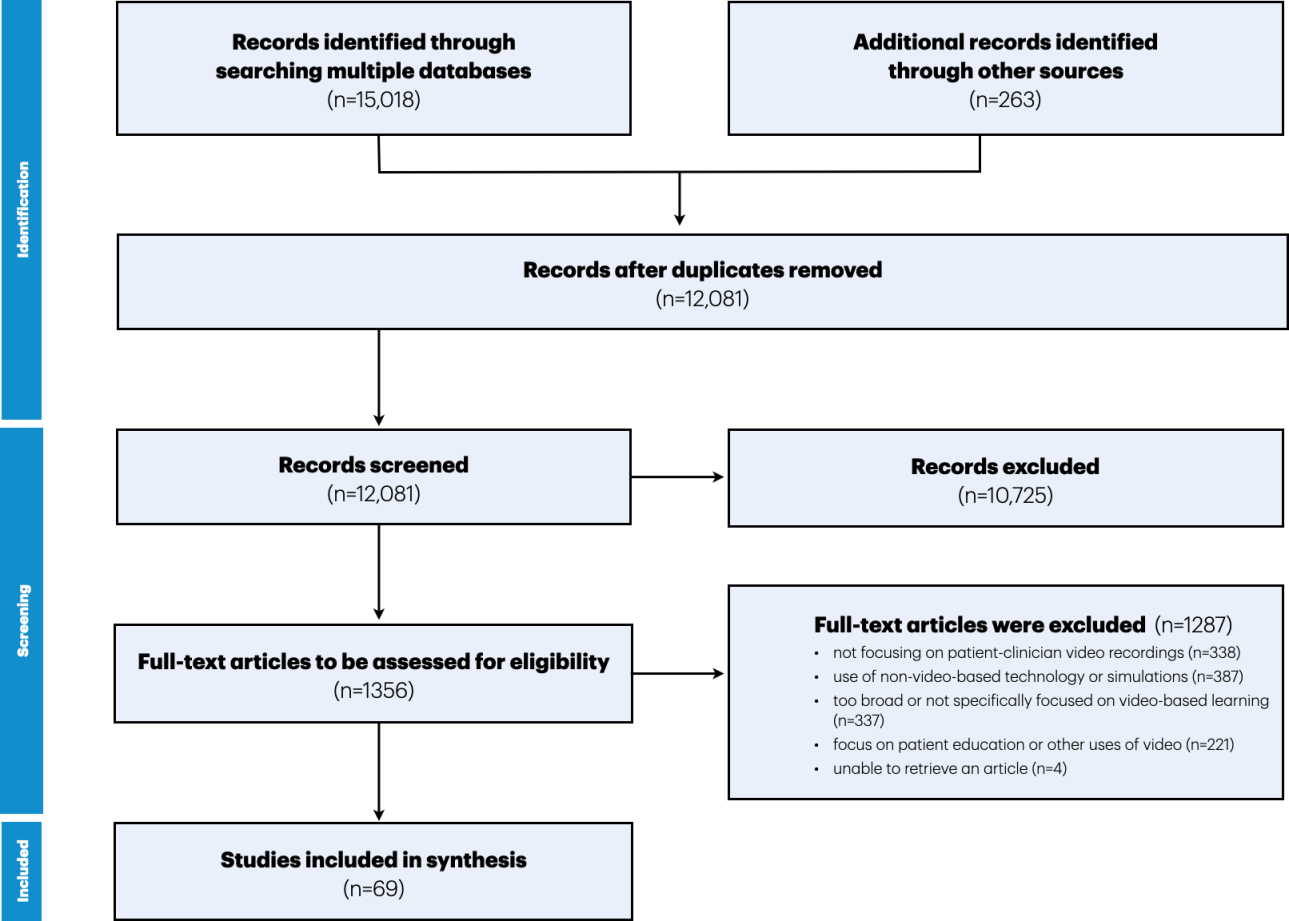
PRISMA flow diagram showing inclusion and exclusion strategy [26].

The search strategy yielded a total of 15,281 records, of which 12,081 were screened after the removal of duplicates. Following a thorough examination of the full texts, 69 studies were identified as suitable candidates for inclusion in the scoping review [15,20,28-94]. The exclusion of patient-clinician video recordings, the use of non–video-based or irrelevant technologies, and noneducational contexts were identified as the most common reasons for exclusion. The comprehensive selection process is delineated in Figure 1, which details the identification, screening, eligibility, and inclusion outcomes.

### Characteristics of Sources Evidence

Multimedia Appendix 2 summarizes each individual source of evidence’s objectives, target population, research setting, research design, data collection methods, outcome measures, and primary findings. The references for each study are provided in the list of sources alongside the relevant details.

### Synthesis of Results

#### Characteristics of the Included Documents

The 69 included studies demonstrate a clear increase in research interest in video-based learning over time, with the majority of these studies having been published between 2011 and 2020 (n=41, 59%) (Table 1). This trend underscores the growing recognition of video-based learning as a valuable tool in health education.

**Table 1.** Characteristics of the included studies by publication year, article type, research design, and data type (n=69).

Characteristics	Studies, n (%)
Publication year (n=69)	
Before 2000	3 (4)
2001‐2010	14 (20)
2011‐2020	41 (59)
Since 2021	11 (16)
Data type (n=62)	
Mixed methods	29 (47)
Quantitative	17 (27)
Qualitative	16 (26)
Type of article and research design (n=69)	
Original research article	
Quasi-experimental design	23 (33)
Observational design	15 (22)
Descriptive design	5 (7)
Randomized controlled trial	5 (7)
Phenomenological design	4 (6)
Experimental design	4 (6)
Comparative design	2 (3)
Case study design	2 (3)
Cross-sectional design	1 (1)
Nonexperimental design	1 (1)
Review article	
Literature review	4 (6)
Scoping review	2 (3)
Systematic review	1 (1)
Place of publication (n=69)	
North America	
United States	33 (48)
Canada	3 (4)
Europe	
United Kingdom	5 (7)
Sweden	5 (7)
Denmark	2 (3)
Netherlands	1 (1)
Norway	1 (1)
Spain	1 (1)
Ireland	1 (1)
Germany	1 (1)
Asia	
South Korea	3 (4)
Turkey	2 (3)
China	2 (3)
Saudi Arabia	1 (1)
Japan	1 (1)
India	1 (1)
Oceania	
Australia	4 (6)
Africa	
South Africa	2 (3)

In terms of geography, the largest proportion of studies originated from North America (n=36, 52%), with the United States contributing 33 (48%) of these. The other regions represented were Europe (n=17, 25%), Asia (n=10, 15%), Oceania (n=4, 6%), and Africa (n=2, 3%). This distribution illustrated the global adoption of this educational approach (Table 1).

A significant proportion of the included studies were original research articles (n=62, 90%). The most prevalent research designs were quasi-experimental (n=23, 33%) and observational (n=15, 22%), reflecting a focus on applied educational interventions. Methodologically, the most prevalent approach was that of mixed methods studies (29/62, 47%), followed by quantitative (17/62, 27%) and qualitative (16/62, 26%) designs. This distribution reflects increasing recognition of the importance of combining quantitative outcomes with contextual, learner-centered perspectives when evaluating video-based learning. The extensive use of original datasets, in conjunction with the emphasis on the cultivation of clinical and communication skills, rather than merely theoretical knowledge, further reinforces the applied, practice-oriented character of video-based learning research. For a comprehensive overview of the breakdowns of research designs and data types, please refer to Table 1.

#### Educational Contexts and Learner Populations

The majority of studies were conducted in the field of medical education (n=28, 41%), followed by dental education (n=15, 22%) and nursing education (n=8, 12%). Regarding the level of learners, the overwhelming focus was on undergraduate students, who were the subject of 50 studies (73%) and accounted for 4648 participants, or 83% of the total sample (n=5685). Please refer to Table 2 for details on the distribution by health field and learner level.

**Table 2.** Distribution of included studies and sample sizes by health discipline and education level.

	Studies (N=69), n (%)	Sample size (N=5685), n (%)
Health field of study		
Medical education	28 (41)	2170 (38)
Dental education	15 (22)	1298 (23)
Nursing education	8 (12)	317 (6)
Interprofessional education (IPE) in health care	4 (6)	903 (16)
Occupational therapy education	4 (6)	294 (5)
Speech-language therapy education	3 (4)	75 (1)
Veterinary medical education	3 (4)	600 (11)
Pharmacy education	2 (3)	8 (0)
Nutrition and dietetics education	1 (1)	12 (0)
Physiotherapy education	1 (1)	8 (0)
Educational level		
Undergraduate	50 (72)	4648 (82)
Postgraduate	5 (7)	97 (2)
Undergraduate and postgraduate	3 (4)	515 (9)
Master	3 (4)	114 (2)
Undergraduate and master’s	1 (1)	14 (0)
Doctorate	1 (1)	24 (0)
Not stated	3 (4)	181 (3)
Not applicable	3 (4)	92 (2)

#### Types and Characteristics of Interventions and Comparators

Intervention types were grouped into 3 main categories: PCIs, simulated PCIs, and other methods of video-based education (Table 3). One study contributed two intervention types to the total of 70 instances across 69 studies.

The most frequently used intervention across the included studies was video recordings of PCIs, either in their raw form or for review and reflection purposes. This category accounted for 39 (56%) out of the 70 recorded intervention types. The second-largest intervention category was simulated PCIs, present in 22 (31%) out of 70 intervention types.

The comparators varied widely. The most common approach was to use a control or no-intervention group, which was used in 32 (46%) out of 69 studies. Traditional teaching methods, such as lectures and textbooks, were used as comparators in 26 (38%) out of 69 studies (Table 4). One study contributed two comparator types to the total of 70 instances across 69 studies.

**Table 3.** Intervention and subintervention types of the included studies (n=69). One study reported dual interventions (n=70 across 69 studies; see Multimedia Appendix 2).

Intervention/subintervention type	Studies, n (%)
Patient-clinician interactions	
Video recording of patient-clinician interactions	24 (34)
Review of video recording of patient-clinician interactions	14 (20)
First-person video recording of patient-clinician interactions	1 (1)
Simulated patient-clinician interactions	
Video recording of simulated patient-clinician interactions	15 (21)
Review of video recording of simulated patient-clinician interactions	6 (9)
First-person video recording of simulated patient-clinician interactions	1 (1)
Other	
Video case-based learning	4 (6)
Video-based assessment	3 (4)
Various video-based education methods	2 (3)

**Table 4.** Comparator types of the included studies (n=69). One study reported dual comparators (n=70 across 69 studies; see Multimedia Appendix 2).

Comparator type	Studies, n (%)
No intervention/control group	32 (46)
Traditional methods	26 (37)
Simulated PCIs^a^	5 (7)
Patient-clinician interactions	3 (4)
Static video recordings of simulated PCIs	2 (3)
Self-guided learning	1 (1)
Various	1 (1)

a PCIs: patient-clinician interactions.

The average duration of video-based interventions across all studies was approximately 20.37 minutes. Those involving PCIs exhibited considerable variation, with an overall mean duration of 19.44 minutes. Video recordings of PCIs were the longest on average (24.53 minutes), followed by reviews of these recordings (23.29 minutes). By contrast, simulated interactions tended to be shorter, particularly reviews of simulated recordings (5.67 minutes) and first-person perspectives (10.50 minutes) (Table 5).

**Table 5.** Mean duration of different types of PCI^a^ interventions.

Intervention/subintervention type	Mean duration (min)
PCIs	
Video recording of PCIs	24.53
Review of video recording of PCIs	23.29
First-person video recording of PCIs	10.50
Simulated PCIs	
Video recording of simulated PCIs	22.22
Review of video recording of simulated PCIs	5.67
First-person video recording of simulated PCIs	10.50

a PCI: patient-clinician interaction.

#### Outcome Categories and Subcategories

**Figure 2.**
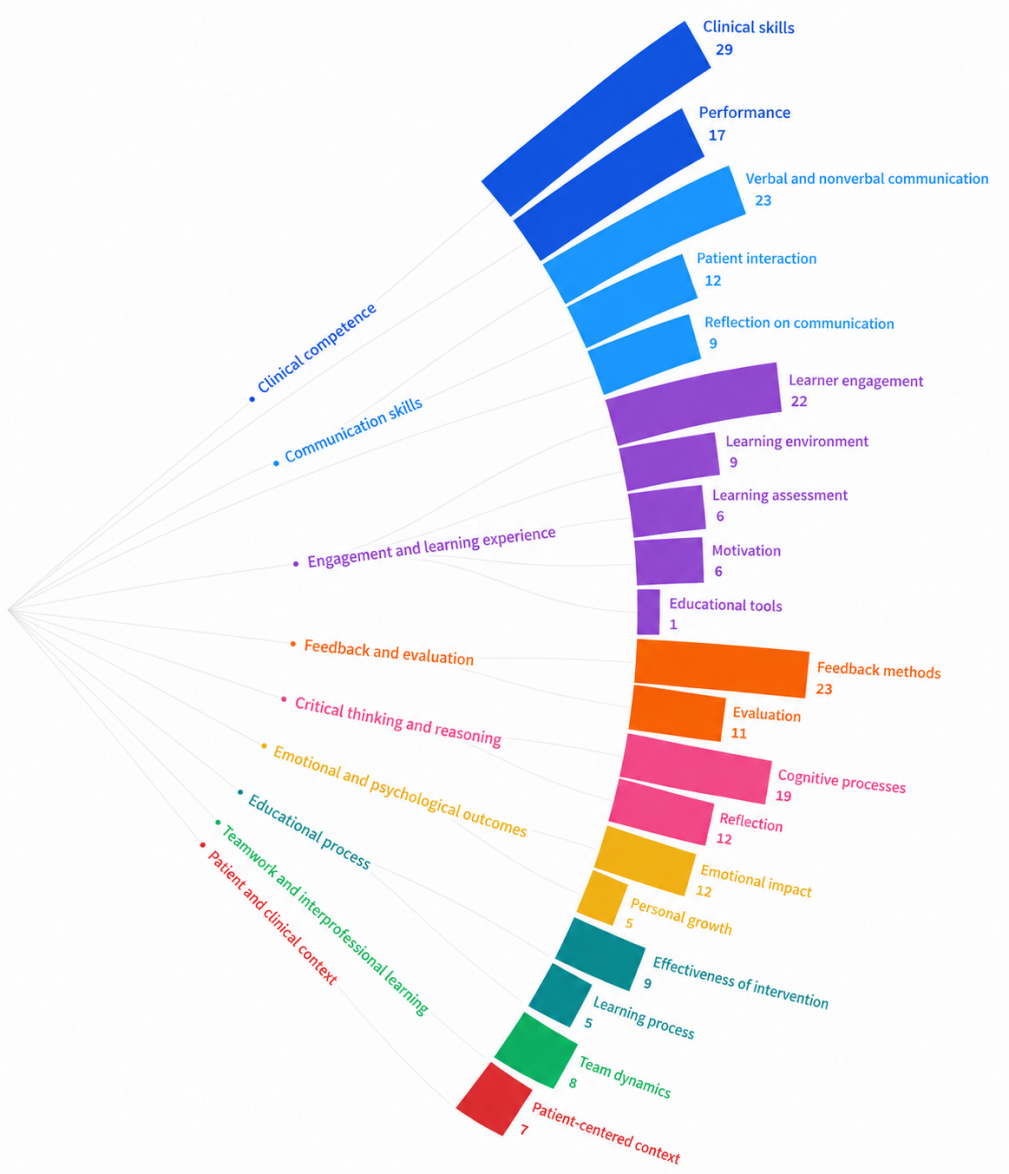
Radial hierarchical bar chart illustrating the distribution of outcome variables across educational domains. Bar length corresponds to the frequency of outcomes within each category.

A total of 226 outcome variables were extracted and categorized within a structured framework of domains, subcategories, and specific variables (Figure 2; MultimediaAppendices 4  5).

The category most frequently assessed was clinical competence, accounting for 46 (20%) out of 226 outcomes. Within this domain, clinical skills comprised the majority (29/226, 13%), with a particular focus on clinical reasoning, diagnostic accuracy, technical skills, and procedural application. A further 17 (8%) out of 226 outcomes focused on clinical performance, including metrics such as performance assessments, efficiency, and reliability.

Communication skills were the second-largest outcome domain, with 44 (19%) out of 226 outcomes. This category was subdivided into verbal and nonverbal communication (23/226, 10%), patient interaction and empathy (12/226, 5%), and communication-focused reflection (9/226, 4%). Common variables included communication behaviors, nonverbal awareness, and patient-centeredness.

The third most prominent category was engagement and the learning experience. This category comprised 44 (19%) out of 226 outcomes and was divided into 4 subcategories: learner satisfaction, perceptions, motivation, and interaction with the educational environment. Learner engagement alone was reported in 22 (10%) out of 226 outcomes, highlighting the importance of video-based learning in encouraging active participation and boosting learners’ confidence.

Further outcome domains included critical thinking and reasoning (23/226, 10%), which focused on knowledge retention, problem identification and self-reflection, and feedback and evaluation (23/226, 10%), which encompassed feedback quality, feedback from standardized patients, and self-assessment metrics.

Emotional and psychological outcomes were documented in 17 (8%) out of 226, addressing emotional responses, confidence, affective learning, and personal development. Less frequently reported categories included educational process outcomes (n=14, 6%), such as the perceived effectiveness of learning tools, as well as outcomes related to teamwork and interprofessional learning (n=8, 4%), and outcomes related to the patient-centered clinical context (n=7, 3%).

#### Assessment Instruments and Measurement Tools

A total of 101 instruments were extracted and categorized within a structured framework of domains, subcategories, and specific instruments. The detailed list of specific instruments and their classifications can be found in Multimedia Appendix 6.

The most frequently used instruments were questionnaires and surveys (34/101, 34%), predominantly general, with a smaller proportion addressing specific topics including validated tools such as the Care Plan Evaluation [31], the Compassionate Care Interactions Questionnaire [53], the Froehlich Communication Survey [31], and the Interprofessional Collaborative Competencies Attainment Survey (ICCAS) [31]. Observation and analysis tools accounted for 20 out of 101 instruments (20%), including conversation and thematic analysis, microanalysis, and structured observation checklists. Interviews and focus groups were used in 12 out of 101 instruments (12%), primarily in the form of focus group discussions and semistructured interviews. Technical and performance assessments were documented in 12 out of 101 instruments (12%), predominantly as “objective and structured assessments” (n=8, 8%), such as the Bariatric Objective Structured Assessment of Technical Skill (BOSATS) and the Objective Structured Assessment of Technical Skill (OSATS) [28,62]. Performance metrics were documented in 4 out of 101 instruments (4%), focusing on clinical documentation and diagnostic accuracy. For a comprehensive list of instruments and their characteristics, please refer to Table 6.

**Table 6.** Categorization of instruments of the included studies by category and subcategory (n=101).

Category/subcategory	Studies, n (%)
Questionnaires and surveys	
General surveys	28 (28)
Specific topic-based questionnaires	6 (6)
Observations and analysis tools	
Conversation and thematic analysis	8 (8)
Transcription and coding	6 (6)
Microanalysis and critical thinking	3 (3)
Observation tools	3 (3)
Interviews and focus groups	
Focus group discussions	6 (6)
Semistructured interviews	5 (5)
Video elicitation interviews	1 (1)
Technical and performance assessments	
Objective and structured assessments	8 (8)
Performance metrics	4 (4)
Peer and self-assessment	
Self-assessments	7 (7)
Peer and faculty assessments	2 (2)
Feedback and satisfaction tools	
Feedback mechanisms	6 (6)
Peer-led approaches	1 (1)
Grading and rubrics	
Grading tools	3 (3)
Data analytics	
Activity logs and analytics	2 (2)
Specialized instruments and scales	
Specialized questionnaires and scales	5 (5)

#### Ethical Implications and Logistical Challenges

##### Ethical Implications and Risk Mapping

Out of the 69 studies included in this scoping review, only 34 (49%) explicitly addressed ethical considerations, which were grouped into 6 primary domains, as summarized in Table 7. The detailed list of ethical considerations can be found in Multimedia Appendix 7.

**Table 7.** Categorization of ethical considerations addressed in the included studies (n=69).

Ethical considerations	Definition	Studies, n (%)
Informed consent	Process for obtaining explicit permission from patients before recording their interactions with clinicians	33 (48)
Confidentiality and privacy	Ensure the protection of patient information through secure handling and anonymization	33 (48)
Ethical use of recordings	Ensure that recorded patient-clinician interactions are used only for their intended educational or research purposes, with clear guidelines and appropriate consent for any secondary use	9 (13)
Emotional impact	Recognizing and mitigating the psychological effects that recording clinical interactions may have on patients and clinicians, such as stress, discomfort, or altered behavior	8 (12)
Regulatory compliance	Adhere to legal and institutional standards, such as privacy laws and ethical guidelines, to ensure the proper handling, storage, and use of recordings in clinical and educational settings	6 (9)

*Informed consent* and *confidentiality and privacy* were reported in 33 (48%) out of 69 studies each, reflecting a moderate emphasis on obtaining explicit permission and protecting data. The *ethical use of recordings* was discussed in 9 out of 69 studies (13%), focusing on restricting use to approved educational or research purposes. Of the 69 studies, 8 (12%) considered the emotional impact of recording on participants, and 6 (9%) mentioned compliance with regulations and institutional policies.

**Figure 3.**
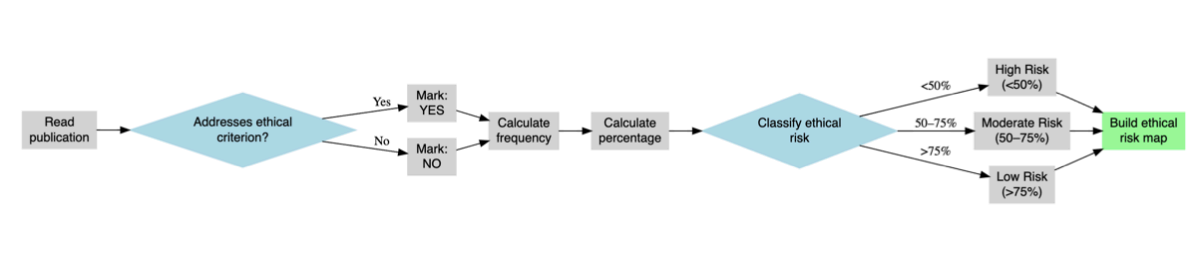
Flowchart of risk mapping for ethical considerations in the included studies.

Ethical considerations were categorized into 3 risk levels based on prevalence: low risk (addressed in more than 75% of relevant studies), moderate risk (addressed in 50%‐75% of studies), and high risk (addressed in less than 50% of studies), as illustrated in Figure 3. Informed consent and confidentiality and privacy were classified as low risk relative to studies that addressed ethical issues but as high risk in absolute terms, indicating that these considerations were omitted in over half of the total sample (Table 8). Other considerations, including emotional impact, ethical use of recordings, and regulatory compliance, were classified as high risk under both relative and absolute criteria.

**Table 8.** Risk map of ethical considerations in the included studies (n=69).

Ethical considerations	Risk level (relative)	Relative %	Risk level (absolute)	Absolute %
Informed consent	Low risk	100	High risk	48
Confidentiality and privacy	Low risk	100	High risk	48
Emotional impact	High risk	24	High risk	12
Ethical use of recordings	High risk	27	High risk	13
Regulatory compliance	High risk	18	High risk	9

##### Logistical Challenges

A total of 69 studies were included in this scoping review, of which 33 (48%) addressed logistical challenges. These challenges were categorized into 5 core domains, as detailed in Table 9. A comprehensive list of these challenges can be found in Multimedia Appendix 8.

As shown in Table 9, the most frequently reported challenges across studies were technical setup (33/69, 48%) and scheduling coordination (30/69, 43%). Additionally, less frequently reported challenges included resource allocation (14/69, 20%), training for participants (8/69, 12%), and feedback mechanisms (6/69, 9%). These findings underscore the need to address these key areas to optimize implementation processes, as technical setup and scheduling coordination are critical factors that impact study outcomes.

**Table 9.** Categorization of logistical considerations addressed in the included studies (n=69).

Logistical consideration	Definition	Studies, n (%)
Technical setup	Ensure that recording equipment is properly set up and functioning, including video/audio recording technology and connectivity.	33 (48)
Scheduling coordination	Coordinate patient, clinician, and technical staff schedules to ensure recording sessions are timely and uninterrupted.	30 (43)
Resource allocation	Manage the allocation of technical resources, including equipment, time, and teacher support for recording sessions.	14 (20)
Training for participants	Provide appropriate training for staff, clinicians, and students in the use of recording technology and protocols.	8 (12)
Feedback mechanisms	Organize feedback sessions and ensure appropriate processes are in place to provide reflective feedback to participants.	6 (9)

## Discussion

This scoping review systematically mapped the use of video recordings of PCIs in health education, with a focus on the following areas: types of recordings, educational purposes, comparative effectiveness, impact on learner outcomes, and key ethical and logistical considerations.

### Principal Findings

This scoping review identified 69 studies examining the use of video recordings of PCIs for health education purposes. The majority of these studies focused on medical education (28/69, 41%), followed by dental education (15/69, 22%) and nursing education (8/69, 12%). This highlights the widespread emphasis on clinical skill development across health disciplines. Of the 69 included studies, 41 (59%) were published between 2011 and 2020, reflecting a growing interest in video-based pedagogical tools. The analyzed interventions included real and simulated patient-clinician encounters, with real clinical interactions predominating.

Video-based learning was generally associated with improvements in key educational outcomes, particularly in the domains of clinical competence, communication skills, and learner engagement. Where available, comparisons with traditional instructional methods suggested that video recordings may provide advantages or complementary benefits for experiential and reflective learning. However, the heterogeneity of study designs, outcome measurements, and assessment tools limited the comparability of the findings, preventing definitive conclusions from being drawn regarding their overall efficacy of video-based learning.

Ethical considerations, particularly informed consent and confidentiality, were addressed inconsistently across studies, mapping these as high-risk areas. Logistical challenges, including technological limitations and scheduling constraints, were also commonly reported. Despite these limitations, video recordings of PCIs were consistently perceived by educators and learners alike as valuable, versatile, and pedagogically effective tools in the context of contemporary health education.

### Comparison to Prior Work

The findings of this scoping review indicate that video recordings of PCIs are increasingly incorporated into health professions education and are widely regarded as valuable pedagogical tools for supporting the development of core clinical competencies. The reviewed literature highlights the use of PCI recordings across a variety of educational objectives, including enabling learners to observe complex, real-world clinical interactions; fostering reflective practice; and providing structured opportunities for formative feedback [95,96]. These uses are indicative of a more general pedagogical shift toward learner-centered, active educational strategies, which are grounded in well-established theoretical frameworks such as experiential learning, social learning, and constructivism [3,97,98].

PCI recordings facilitate the experiential learning theory model by enabling learners to engage with authentic clinical scenarios in a safe and repeatable format, promoting both observation and reflection prior to active clinical engagement [99,100]. In a similar vein, Bandura’s social learning theory emphasizes the significance of modeling and observational learning in the acquisition of behavior [98]. PCI recordings facilitate the observation of expert communication strategies, diagnostic reasoning, and interpersonal conduct in action, thereby providing a reference point for the learner’s own professional practice [20].

The extant literature also positions PCI recordings as an effective bridge between theoretical instruction and clinical application. This function is underpinned by constructivist learning theory, which posits that learners build knowledge through contextual engagement and integration of new information into existing cognitive frameworks [101,102]. PCI recordings situate learning within the complexity of real clinical contexts, offering nuanced representations of uncertainty, interpersonal dynamics, and decision-making. Furthermore, the principles of cognitive load theory provide additional support for the use of video as a tool to scaffold complex learning [100,102]. The provision of multimodal content, encompassing visual, auditory, and contextual elements, is a salient feature of PCI recordings. This multimodal approach has been demonstrated to facilitate the management of both intrinsic and extraneous cognitive load, a phenomenon that is particularly pronounced when these recordings are used in conjunction with instructional scaffolding or expert narration [12,103,104].

It is important to note that PCI recordings also support the development of metacognitive skills and professional identity formation. The observation of actual clinicians engaged in complex patient interactions serves to encourage learners to engage in reflection on their future roles, values, and behaviors as health professionals [105,106]. This approach is in alignment with the emerging educational goals that place a premium not only on competence, but also on ethical reasoning, self-awareness, and identity development [107-110]. Consequently, the use of PCI recordings transcends the delivery of content, thereby supporting the comprehensive professional development of learners.

### Strengths and Limitations

This scoping review is strengthened by its comprehensive, methodologically rigorous design following Joanna Briggs Institute guidelines. A comprehensive search strategy encompassing multiple databases (ie, MEDLINE, Embase, and ERIC) and gray literature was used to enhance the scope of the study and mitigate publication bias. The implementation of dual independent screening and data extraction processes served to ensure the consistency of the review and to minimize the impact of reviewer bias.

This study focuses specifically on video recordings of authentic or simulated PCIs, rather than generic video tools. This approach allows for a detailed examination of unique pedagogical, ethical, and implementation issues. The incorporation of studies from diverse health professions has been demonstrated to enhance the generalizability of research findings.

It is important to note several limitations of the study. First, substantial heterogeneity in study design, interventions, and outcomes limited the comparability of the studies, and thus prevented meta-analysis. The paucity of standardized, validated outcome measures, coupled with the dearth of direct comparisons with traditional methods, serves to impede the ability to draw definitive conclusions regarding the effectiveness of the latter. Second, the majority of studies assessed short-term outcomes exclusively, thereby leaving the question of long-term impacts on clinical performance and patient care unanswered. Third, ethical and logistical considerations were inconsistently reported, reflecting variable adherence to institutional and regulatory frameworks (eg, HIPAA), which complicates establishing best practices. Fourth, practical barriers such as limited access to recording, privacy concerns, faculty training gaps, and administrative burdens were frequently mentioned but insufficiently detailed. Finally, the restriction of the review to English-language publications may have resulted in the exclusion of pertinent non-English studies, and some unpublished or nonindexed evidence may have been overlooked.

### Future Directions

It is recommended that future research efforts concentrate on the development and widespread adoption of standardized, validated outcome measures. The objective of this recommendation is to enable rigorous evaluation and meta-analytic synthesis of PCI-based educational interventions. The necessity for well-designed longitudinal and controlled studies is evident in order to elucidate the sustained effects of PCI recordings on clinical competence, behavioral change, and patient-centered outcomes.

There is a need for improvement in the consistency of reporting and adherence to ethical standards, including informed consent and data protection protocols. The establishment of formal institutional policies is necessary to ensure regulatory compliance and to safeguard the rights of participants. In order to achieve this, a coordinated effort is required among educators, clinicians, institutional review boards, and legal authorities to develop ethical frameworks that are scalable and practical. These frameworks must balance educational efficacy with legal and moral obligations. Empirical evaluation of these frameworks’ impact on feasibility, learner outcomes, and patient perceptions is warranted to inform implementation best practices.

In order to address logistical barriers, there is a necessity for institutional commitment to resource allocation, the development of scalable technological solutions, comprehensive faculty development, and streamlined administrative processes. The identification and dissemination of effective implementation strategies will be vital for the successful integration of PCI recordings into health professions curricula.

Furthermore, the expansion of the research scope to encompass a range of health professions, educational settings, and non-English language publications is expected to enhance the external validity and global applicability of findings.

Collectively, these directions are essential to advance the evidence base and optimize the integration of PCI recordings within health professions education.

### Conclusions

This scoping review systematically maps the current landscape of video recordings of PCIs in health education. A synthesis of 69 studies shows that video-based methodologies are being adopted more widely in medical, dental, and nursing curricula. There is robust evidence that these methodologies are more effective than traditional methods in improving clinical skills, communication, and learner engagement.

However, despite these educational benefits, the review identifies critical and persistent gaps in ethical and logistical domains. Informed consent and confidentiality were addressed inconsistently, highlighting the need for standardized ethical protocols and rigorous safeguarding of patient data. Logistical barriers, including technical setup, scheduling, and resource allocation, remain significant impediments to widespread implementation.

A notable finding is the marked heterogeneity in outcome measures and assessment instruments, which limits comparability and synthesis across studies. This emphasizes the urgent need for the development and adoption of standardized, validated outcome sets and measurement tools in future research.

Taken together, these findings emphasize the transformative potential and complex challenges of integrating video recordings into health education. Responsible and effective use of this technology depends on establishing robust ethical frameworks, standardized methodologies, and institutional commitment to best practices. Prioritizing these issues will be crucial for maximizing the educational impact and ensuring the sustainability of video-based learning in health professions education.

## Supplementary material

10.2196/70324 Multimedia Appendix 1 Search strategy.

10.2196/70324 Multimedia Appendix 2 Summary of extracted data.

10.2196/70324 Multimedia Appendix 3 Definitions of data items.

10.2196/70324 Multimedia Appendix 4 Categorization of outcome variables of the included studies by author and year, category, subcategory, general outcome variable, and specific outcome variable.

10.2196/70324 Multimedia Appendix 5 Categorization of outcome variables of the included studies by category, subcategory, and general outcome variable.

10.2196/70324 Multimedia Appendix 6 Categorization of specific instruments of the included studies by author and year, category, and subcategory.

10.2196/70324 Multimedia Appendix 7 Categorization of ethical considerations addressed in the included studies.

10.2196/70324 Multimedia Appendix 8 Categorization of logistical considerations addressed in the included studies.

10.2196/70324 Checklist 1 PRISMA-ScR checklist.

10.2196/70324 Checklist 2 CADTH Grey Matters checklist.

## Abbreviations

BOSATS: Bariatric Objective Structured Assessment of Technical Skill
CADTH: Canadian Agency for Drugs and Technologies in Health
ERIC: Education Resources Information Center
HIPAA: Health Insurance Portability and Accountability Act
ICCAS: Interprofessional Collaborative Competencies Attainment Survey
OSATS: Objective Structured Assessment of Technical Skill
PCI: patient-clinician interaction
PICO: population, intervention, comparison, and outcomes
PRISMA-ScR: Preferred Reporting Items for Systematic Reviews and Meta-Analyses Extension for Scoping Reviews
