# Supplementary material for: Video Recording of Patient-Clinician Interactions in Health Education: Scoping Review
Source: JMIR Med Educ. 2026 Jul 13;12:e70324. doi: 10.2196/70324 (PMC13361625; doi:10.2196/70324)
Supplement: Multimedia Appendix 1 [file mededu-v12-e70324-s001.docx]

## Search Strategy

#

# This document delineates the exhaustive search strategy deployed in search databases to identify pertinent literature on video recordings of patient-clinician interactions in health education. The strategy incorporates particular search terms, Boolean operators and filters, which are employed in order to refine the search results.

Date Range: Inception to 30 October 2024

**Interface: PubMed**

Controlled Vocabulary: MeSH (Medical Subject Headings)

| **Search #** | **Search String** | **Rationale** |
| --- | --- | --- |
| #1 | video recording*[tiab] OR video record*[tiab] OR video record*[MeSH Terms] | Core concept: Video recording presence |
| #2 | patient* clinician* interac*[tiab] OR patient-clinician interaction*[MeSH] | Core concept: Patient-clinician interactions |
| #3 | (medical education[MeSH] OR nursing education[MeSH] OR dental education[MeSH] OR health professions education[MeSH]) AND (video* OR recording*) | Context: Health professions education |
| #4 | (clinical skill* OR communication skill* OR technical skill*)[tiab] AND (video* OR recording*) | Outcome: Clinical skills via video |
| #5 | (simulated patient* OR standardized patient*)[tiab] AND (video* OR recording*) | Intervention type: Simulated scenarios |
| #6 FINAL | (#1 OR #2) AND (#3 OR #4 OR #5) AND (english[la]) NOT (conference paper[pt] OR editorial[pt]) | Combined with limits |

#

#

# Execution Details

#

# Number of Results Retrieved: 6.977 results

#

# Filters Applied

#

# None

# Additional Notes

# This search strategy was developed by a researcher and peer-reviewed by a more experienced member of the research team to ensure thoroughness and accuracy. Parallel search structures were subsequently applied to additional databases, including EMBASE and ERIC, to enhance the comprehensiveness of the literature review. The final search results were exported into Zotero, where duplicates were systematically removed by a researcher. For further details or queries regarding the search strategy, please contact the corresponding author.

Date Range: Inception to 30 October 2024

Date Range: Inception to 30 October 2024

**Interface: Elsevier EMBASE**

Controlled Vocabulary: Emtree (EMBASE Thesaurus)

| **Search #** | **Search String** | **Rationale** |
| --- | --- | --- |
| #1 | 'video recording':de OR 'video recording':ab,ti | Core concept: Video recording (Emtree descriptor) |
| #2 | 'patient interaction':de OR 'clinician patient communication':de | Core concept: Patient-clinician interaction |
| #3 | ('medical education':de OR 'nursing education':de OR 'dental education':de OR 'health professional education':de) AND ('video':ab,ti OR 'recording':ab,ti) | Context: Health education |
| #4 | ('clinical competence':de OR 'communication skill':de OR 'technical skill':de) AND ('video':ab,ti OR 'recording':ab,ti) | Outcome: Clinical outcomes |
| #5 | ('simulated patient':de OR 'standardized patient':de) AND ('video':ab,ti OR 'recording':ab,ti) | Intervention type |
| #6 FINAL | (#1 OR #2) AND (#3 OR #4 OR #5) AND [english]/lim NOT ([conference paper]/pt OR [editorial]/pt) | Combined with limits |

# Execution Details

# Number of Results Retrieved: 7.778 results

# Filters Applied

Publication Type: Exclude editorials, conference papers

Date: No restriction

# Additional Notes

None.

Date Range: Inception to 30 October 2024

**Interface: ERIC (Education Resources Information Center)**

Controlled Vocabulary: ERIC Thesaurus Descriptors

| **Search #** | **Search String** | **Rationale** |
| --- | --- | --- |
| #1 | DE="Video Recording" OR TI="video recording*" OR AB="video recording*" | Core concept: Video recording (ERIC descriptor) |
| #2 | DE="Clinical Experience" OR DE="Patient Care" OR TI="patient* clinician*" OR AB="patient-clinician interaction*" | Core concept: Clinical interactions |
| #3 | (DE="Health Education" OR DE="Medical Education" OR DE="Nursing Education" OR DE="Dental Education") AND (TI="video*" OR AB="video*" OR TI="recording*" OR AB="recording*") | Context: Health education + video |
| #4 | (DE="Clinical Skills" OR DE="Communication Skills" OR TI="clinical competence*" OR AB="clinical competence*") AND (TI="video*" OR AB="video*") | Outcome: Clinical skills via video |
| #5 | (DE="Simulation" OR TI="simulated patient*" OR AB="simulated patient*") AND (TI="video*" OR AB="video*") | Intervention type |
| #6 FINAL | (#1 OR #2) AND (#3 OR #4 OR #5) NOT (Publication Type="Conference Paper" OR Publication Type="Report - Non-available from ED/ERIC") | Combined with limits |

# Execution Details

# Number of Results Retrieved: 526 results

# Filters Applied

Publication Type: Exclude non-peer-reviewed conference papers

Date: No restriction

# Additional Notes

None.
