# Supplementary material for: Video Recording of Patient-Clinician Interactions in Health Education: Scoping Review
Source: JMIR Med Educ. 2026 Jul 13;12:e70324. doi: 10.2196/70324 (PMC13361625; doi:10.2196/70324)
Supplement: Multimedia Appendix 2 [file mededu-v12-e70324-s002.docx]

| **(Author, Year)** | **Origin** | **Type of Article** | **Research Design** | **Data Type** | **Aims/Purpose** | **Student Population** | **Year of Study** | **Study Cycle** | **Area of Study** | **Sample Size** | **Intervention Type** | **Duration** | **Comparator** | **Outcome Variables** | **Instruments** | **Main Findings** |
| --- | --- | --- | --- | --- | --- | --- | --- | --- | --- | --- | --- | --- | --- | --- | --- | --- |
| (Batteson, 2023) [31] | USA | Original Research Paper | Quasi-experimental design | Mixed methods | To develop and assess a video simulation tool ("Meeting the Sodos") for teaching interprofessional education (IPE) competencies and social determinants of health (SDH) across two universities. | Health professional students | First-year | Undergraduate | Interprofessional Education (IPE) in healthcare | 754 participants | Video simulation of patient-clinician encounter | Not specified | No intervention | Interprofessional competencies (teamwork, communication, roles and responsibilities, values and ethics)  Learning Assessment  Identification of Social Determinants of Health | IPEC Competency Complexity Questionnaire  Interprofessional Collaborative Competencies Attainment Survey (ICCAS)  Froehlich Communication Survey  Care Plan Evaluation | The study found significant improvements in students' understanding of interprofessional education competencies, effective use of simulated patient encounters to enhance awareness of social determinants of health, improved collaborative and communication skills in interprofessional groups, a heightened ability to identify and address SDH issues, and the broad applicability of the "Meeting the Sodos" educational tool across different institutions. |
| (Henry, 2020) [20] | USA | Original Research Paper | Observational design | Mixed methods | To provide practical advice for conducting interdisciplinary research involving video-recorded clinical encounters and to emphasize the importance of integrating diverse perspectives in research | Health professional students | Not stated | Not stated | Interprofessional Education (IPE) in healthcare | 86 participants | Video recordings of patient-clinician interactions | Not specified | Audio recordings of patient-clinician interactions | Communication behaviors  Patient and clinician experiences Educational relevance | Questionnaires  Transcription protocols  Video elicitation interviews  Conversation analysis  Microanalysis of dialogue | This study highlights the value of interdisciplinary collaboration in video-based clinical research, using video recordings to capture both verbal and non-verbal communication for a nuanced analysis of patient-clinician interactions; it addresses challenges like ethical and regulatory considerations, underscores the potential for secondary analyses, and provides practical recommendations for video-based studies that contribute to advancing patient-centered care in complex areas like chronic pain management. |
| (Nissen, 2024) [68] | Australia | Original Research Paper | Phenomenological design | Qualitative | To explore the student experience of using an authentic patient video in IPE. | Health professional students | Not stated | Undergraduate; Master | Interprofessional Education (IPE) in healthcare | 14 participants | Video recordings of patient-clinician interactions | 6 minutes | Written case study | Attitudes Toward Person-Centered Care  Understanding of Interprofessional Roles  Teamwork and Collaboration  Engagement and Learning Experience  Self-Reflection and Critical Thinking | Focus Group Discussions  Interview Guide  Thematic Analysis | The study found that exposure to an authentic patient video significantly shifted students' attitudes from disease-centered to person-centered care, enhanced their understanding of interprofessional roles, fostered greater appreciation for teamwork, and led to positive student engagement and immediate improvements in learning experiences in interprofessional education settings. |
| (Ahmet, 2018) [28] | Turkey | Systematic Review | Not applicable | Not applicable | To explore the influence of video in surgical education | Medical students and residents | Not stated | Undergraduate; Postgraduate | Medical Education | 507 participants | Various video-based education methods | Various | Traditional methods | Overall performance  Technical Skills  Effectiveness of the learning process  Knowledge retention and understanding  Long-term retention of Skills  Trainee Satisfaction and Confort | Global Assessment Scale  Objective Structured Assessment of Technical Skills (OSATS)  Self-Evaluation Test and Observer’s Checklist  Quiz results  Multiple Choice Test  Retention Tests  Expert-Based Assessments | Video-based education improves knowledge, skills, and satisfaction in surgical training compared to traditional methods, though more robust studies are needed to confirm its long-term effectiveness. |
| (Balslev, 2005) [30] | Denmark | Original Research Paper | Quasi-experimental design | Mixed methods | To investigate the impact of video cases on cognitive and metacognitive processes | Medical residents | Not stated | Postgraduate | Medical Education | 16 participants | Video recordings of patient-clinician interactions | 2.5 minutes | Text-based case presentation | Frequency of different types of clauses generated by the participants during their verbal interactions (Data exploration, Theory building, Theory evaluation, Metareasoning) | The group discussions were recorded on videotape, which was subsequently transcribed verbatim for analysis | Video cases enhance cognitive engagement, theory-building, and understanding of clinical presentations among pediatric residents compared to text, though they may reduce metareasoning, highlighting their value as a training supplement for complex topics. |
| (Botelho, 2016) [34] | China | Original Research Paper | Quasi-experimental design | Mixed methods | To create a library of video-recorded consultations to facilitate communal learning among students. | Medical students | Not stated | Undergraduate | Medical Education | 55 participants | Video recordings of patient-clinician interactions | 5 - 14 minutes | Not applicable | Self-Reflection and Peer Feedback  Technical, Practical, and Logistical Feasibility  Engagement with Consultation Videos  Student Feedback | Questionnaires  Transcripts of tutorials  Usage analytics | Mobile-recorded clinical videos were feasible for students to create, enhancing self-reflection, peer discussion, and identification of key learning moments, while providing access to diverse cases and receiving positive feedback, with students requesting more of these resources for future learning. |
| (Chan, 2010) [36] | China | Original Research Paper | Experimental design | Quantitative | To assess preference for video triggers over paper cases in PBL tutorials | Medical students | First-year; Second-year | Undergraduate | Medical Education | 257 participants | Video recording of patient-clinician interactions; Video simulation of patient-clinician interactions | ~3 minutes | Paper case-based learning | Engagement and interest  Clinical reasoning and observational skills  Integration of Learning  Self-Directed Learning | Questionnaire | Video triggers in PBL tutorials enhance engagement, clinical reasoning, skill integration, and self-directed learning by simulating realistic patient interactions, though they cannot fully replace the depth of real patient encounters. |
| (Courteille, 2014) [38] | Sweden | Original Research Paper | Observational design | Mixed methods | To investigate the dynamics and congruence of interpersonal behaviors and socioemotional interaction during a virtual clinical encounter (VCE) and evaluate interaction design characteristics contributing to engagement. | Medical students | Third-year | Undergraduate | Medical education | 30 participants | Video recording of simulated patient-clinician interactions | Not specified | Not applicable | Behavioral Analysis  Log Activity Data  Self-Reported Measures  Affective Learning Outcomes | Video Observational Data  Log Activity Data  Questionnaires  Semi-structured interviews | The virtual clinical environment (VCE) fostered effective clinical management, socioemotional engagement, collaborative learning, and improved communication skills, with students responding positively to the realistic, interactive, and emotionally engaging design elements, though gender-related differences in communication style were observed. |
| (Farnan, 2013) [41] | USA | Original Research Paper | Quasi-experimental design | Mixed methods | To address unprofessional behaviors in inpatient care using video-based education | Medical residents | Not stated | Postgraduate; Not applicable | Medical Education | 244 participants | Review of video recordings of patient-clinician interactions | 4 - 7 minutes | Not specified | Participant engagement  Perception of Usefulness  Intent to change behavior  Perception of video realism  Effectiveness of the interactive materials | Survey  Checklists | The video-based workshop effectively engaged faculty and residents, fostering reflection on unprofessional behaviors, encouraging behavior change—especially among those who found the videos realistic—and positively influencing the learning environment. |
| (Hammoud, 2012) [49] | USA | Literature Review | Not applicable | Not applicable | To determine the effectiveness of video review of student performance during patient encounters as a learning tool in medical education. | Medical students | Pre clinical; Clinical | Undergraduate | Medical Education | Not specified | Review of video recordings of patient-clinician interactions | Not specified | Traditional methods | Communication skills  Effectiveness of expert feedback  Self-Assessment challenges  Student satisfaction | Self-Assessment questionnaires and checklists  Faculty assessment  Peer assessment  Satisfaction surverys  Performance metrics | Video recording of student performance during patient encounters, especially when paired with expert feedback, enhances communication and examination skills, satisfaction, and feedback quality, though improvements in self-assessment accuracy are limited, underscoring the need for structured feedback, faculty development, and further research for optimal impact. |
| (Henry, 2012) [50] | USA | Original Research Paper | Observational design | Mixed methods | To describe and provide guidance on video elicitation interviews in primary care | Physicians | Graduated | Not applicable | Medical Education | 36 participants | Video recordings of patient-clinician interactions | Not specified | Not specified | Participants' Thoughts, Beliefs, and Emotions  Participants' physiological or emotional responses | Interview protocols | Video elicitation interviews provide a nuanced understanding of physician-patient interactions by enhancing recall and reflection, benefiting both research and education in complex communication, though they require careful planning and attention to privacy, logistical challenges, and potential biases. |
| (Ju, 2017) [52] | South Korea | Original Research Paper | Experimental design | Mixed methods | To examine the effect of recorded video monitoring on students’ self-reflection after completing their clinical performance examination. | Medical students | Fifth-year | Undergraduate | Medical education | 57 participants | Review of video recordings of patient-clinician interactions | Not specified | Not specified | Students' performance self-evaluation  Standardized patient evaluation  Usefulness of video monitoring | Self-Assessment Scale  Standardized Patient Evaluation Scores  Qualitative open-ended feedback | Self-monitoring through recorded video feedback led to more objective self-assessments, improved reflective learning, and enhanced patient-physician interaction skills, with students acknowledging its usefulness in identifying areas for improvement and aligning their evaluations with those of standardized patients. |
| (Kalish, 2011) [53] | USA | Original Research Paper | Quasi-experimental design | Mixed methods | To integrate recognition and assessment of student compassionate care into a clinical skills exercise | Medical students | Third-year; Fourth-year | Undergraduate | Medical education | 11 participants | Video recordings of patient-clinician interactions | Not specified | None | Student Self-Assessment  Feedback (preceptor, fourth-year students and patient)  Effectiveness of the intervention | Compassionate Care Interactions Questionnaire  Patient-Partner Questionnaire  Focus Group Discussions | Videotaping patient interactions helped students recognize missed opportunities for compassionate care, improve self-assessment, and align their skills with patient and preceptor feedback, supporting the integration of compassionate care into clinical training. |
| (Kamin, 2003) [55] | USA | Original Research Paper | Experimental design | Quantitative | To determine whether critical thinking in problem-based learning (PBL) group discourse differed according to case modality (text, video, virtual). | Medical students | Third-year | Undergraduate | Medical Education | 128 participants | Video case-based learning | Not specified | Text-based case presentation | Critical-thinking (problem identification, problem description, problem exploration, applicability and integration) | Transcription of discussions  Content analysis coding  Critical-thinking ratios | Video cases in virtual problem-based learning groups enhanced critical thinking and individual accountability, although they showed fewer group dynamics compared to face-to-face groups, highlighting the benefits of visual learning, especially in clinical education. |
| (Lee, 2013) [56] | South Korea | Original Research Paper | Quasi-experimental design | Quantitative | To analyze the effect of interview skill education through videotapes of students’ interviews with real patients in outpatient settings. | Medical students | Not stated | Undergraduate | Medical education | 33 participants | Review of video recordings of patient-clinician interactions | Not specified | No intervention | Specific students' interview skills (Type of question, timeline, positive verbal reinforcement, therapeutic sequence, facilitative behavior, pace of interview, summary and verification, avoidance of jargon, maintaining a respectful tone, addressing another concern)  Overal students' interview performance  Effectiveness of the intervention | Interview skill checklist, modified from the Arizona Clinical Interview Rating Scales (ACIRS) | Videotaping student interviews with real patients, coupled with immediate feedback, significantly improved interview skills, particularly in question types, timelines, and verbal reinforcement, with positive impacts on communication skills, although further research with larger sample sizes is needed to assess long-term effects. |
| (Leeds, 2020) [57] | USA | Original Research Paper | Quasi-experimental design | Quantitative | To evaluate the impact of a narrative-driven video on knowledge and attitudes toward fibromyalgia | Medical student | Third-year | Undergraduate | Medical Education | 54 participants | Video recordings of patient-clinician interactions | 13 minutes | None | Knowledge (students' understanding)  Attitudes (empathy, willingness to treat and perception) | Survey | The patient-centered video on fibromyalgia significantly improved students' knowledge and attitudes towards the condition, with increased empathy and understanding, although long-term retention needs further investigation. |
| (Leng, 2007) [58] | Netherlands | Original Research Paper | Descriptive design | Qualitative | To examine PBL students’ views on the added value of video cases compared to text-based cases in the pre-clinical phase of undergraduate medical education and the conditions for productive use of video in tutorial discussions. | Medical students | Second-year | Undergraduate | Medical education | 30 participants | Video recordings of patient-clinician interactions | 3 - 20 minutes | Text-based case presentation | Perceived Added Value of Video Cases  Conditions for Productive Use of Video Cases | Focus Group Interviews  Audio Recording and Transcription  Thematic Analysis | The study found that video cases enhance learning by providing realistic, engaging, and memorable clinical scenarios, with optimal use requiring structured viewing, appropriate content difficulty, and varied, concise design to foster exploration, motivation, and improved retention. |
| (Leone, 2006) [59] | USA | Literature Review | Not applicable | Not applicable | To explore the use of video as an educational tool in medical education. | Medical trainees and clinical teams | Not stated | Not applicable | Medical Education | Not specified | Video-based assessment | Not specified | Traditional methods | Adherence to guidelines  Clinical competence  Team Function and Communication  Error identification and reduction  Educational reinforcement | Quality Assurance tool  Debriefing sessions  Miller's Pyramid of Competence  Feedback mechanisms | The study highlights the effectiveness of video recordings in neonatal resuscitation for assessing clinical performance, identifying deviations from guidelines, improving competence through regular feedback, enhancing team dynamics, and providing constructive, non-punitive feedback, while also addressing legal and ethical considerations in video use. |
| (Malon, 2014) [61] | Denmark | Original Research Paper | Observational design | Quantitative | To evaluate the effect of a video case teaching program in Pediatrics on student performance in assessing pediatric patients presented as video cases. | Medical students | Not stated | Not stated | Medical Education | 95 participants | Video-based assessment | 0.5 - 1.5 minutes | Standard education without video cases | Overall performance  Single domain performance  Intraobserver and interobserver reliability | Structured assessment tool  Interclass correlation coefficients | The introduction of a video case teaching program led to a significant improvement in students' pediatric assessment skills, as evidenced by increased Rubric scores across all assessment domains, high reliability in scoring, and positive student feedback, although further research is needed to confirm the transferability of these skills to clinical practice. |
| (McQueen, 2019) [62] | Canada | Scoping Review | Not applicable | Not applicable | To explore video-based assessment in surgical training and its utility. | Medical residents | Not stated | Postgraduate | Medical education | Not specified | Video-based assessment | Not specified | Not applicable | Technical skills  Non-technical skills | Objective Structured Assessment of Technical Skills (OSATS)  Global Rating Index for Technical Skills (GRITS)  Global Operative Assessment of Laparoscopic Skills (GOALS)  Bariatric Objective Structured Assessment of Technical Skill (BOSATS)  Non-Technical Skills for Surgeons (NOTSS) | The review found that video-based assessment in surgical education offers enhanced objectivity, time efficiency, and unobtrusive performance capture, supporting its value for formative assessment, feedback, and skill differentiation across varying levels of surgical expertise. |
| (Muench, 2013) [65] | USA | Original Research Paper | Observational design | Qualitative | To outline the infrastructure and processes for implementing video review in medical education. | Medical residents | Graduated | Postgraduate | Medical Education | 36 participants | Review of video recordings of patient-clinician interactions | Not specified | Not specified | Communication Skills  Visit Efficiency  Shared Decision Making  Overall Medical Care  Residents' Satisfaction  Anxiety levels | Checklist based on the Medical Interview Skills Competency Evaluation (MISCE)  Feedback forms  Surveys  Qualitative observations | Video-based assessments improve the evaluation of patient-clinician interactions by offering an objective view of communication, decision-making, and adherence to clinical guidelines, though challenges such as video quality, editing biases, and rater training need to be addressed, with further research recommended to standardize the practice. |
| (Nilsen, 2005) [67] | Norway | Original Research Paper | Phenomenological design | Qualitative | To explore students' experiences of receiving feedback on videotaped consultations. | Medical students | Not stated | Undergraduate | Medical Education | 19 participants | Video recordings of patient-clinician interactions | Not specified | None | Emotional distress and apprehension  Self-esteem and confidence  Perception of the feedback process (constructiveness, supportiveness)  Need for reassurance regarding consultation skills  Overall acceptance of the intervention method | Focus Group Interviews  Audio Recording and Transcription  Phenomenological Qualitative Analysis | Key findings indicate that, although students initially experienced significant anxiety about video feedback sessions, they ultimately found the process constructive and supportive, leading to increased self-confidence and reassurance in their clinical abilities. The study also highlighted the importance of a safe group dynamic in facilitating open, balanced feedback, and recommended introducing video-based feedback earlier in medical training to reduce apprehension and improve students' comfort with feedback. |
| (Noverati, 2020) [69] | USA | Scoping Review | Not applicable | Not applicable | To summarize how video and virtual patients are used in PBL and their effects | Medical students | Not stated | Undergraduate | Medical education | Not specified | Various video-based education methods | Not specified | Paper case-based learning; Standardized patients | Knowledge Acquisition and Retention  Critical Thinking and Clinical Reasoning  Cognitive Load  Authenticity and Memorability  Student Preferences | Surveys and Questionnaires  Standardized tests or assessments  Critical thinking assessments such as the California Critical Thinking Skills Test (CCTST)  Cognitive Load Scales  Qualitative Interviews or Focus Groups | The key findings highlight that videos and virtual patients (VPs) in problem-based learning (PBL) enhance clinical encounter preparation, authenticity, and engagement, although their impact on knowledge and critical thinking is mixed; they increase cognitive load, preferred more by advanced students, support non-linear decision-making, and are best utilized in a blended approach. |
| (Roberts, 2023) [78] | USA | Original Research Paper | Quasi-experimental design | Mixed methods | To assess the feasibility and effectiveness of virtual interactive patient (VIP) encounters in teaching and assessing clinical skills in first-year medical students, particularly in the context of the dissolution of the Step 2 Clinical Skills exam. | Medical students | First-year | Undergraduate | Medical Education | 124 participants | Video recordings of patient-clinician interactions | Not specified | Not specified | Clinical documentation task performance  Student feedback  Completion rates and times | Student feedback surveys  Grading rubric  Clinical documentation tasks | The study found that Virtual Interactive Patient (VIP) encounters successfully integrated clinical skills training into the first-year medical curriculum, engaging students and leading to high proficiency in clinical documentation tasks. Students appreciated the alignment with coursework and the practical, low-stakes learning environment, though some noted that sessions were time-intensive. Overall, VIP encounters were deemed both feasible and effective for early medical training, with potential for further enhancement. |
| (Roland, 2012) [15] | UK | Literature Review | Not applicable | Not applicable | To evaluate the effectiveness of Patient Video Cases (PVCs) as educational interventions | Medical students, healthcare professionals | Not stated | Not stated | Medical education | Not specified | Video recordings of patient-clinician interactions | Not specified | Various | Learner satisfaction  Knowledge gain  Learner behavior  Organizational change | Questionnaires  Knowledge Tests  Clinical Skills Assessments | Despite the concerns regarding validity, nearly all studies reported positive outcomes regarding the use of Patient Video Cases (PVCs) in enhancing educational experiences. Medical students showed improved critical thinking and diagnostic reasoning when exposed to PVCs. |
| (Roland, 2015) [80] | United Kingdom / Denmark | Literature Review | Not applicable | Not applicable | To describe the use of patient video cases (PVCs) in developing observational skills and clinical reasoning in medical education. | Medical students and postgraduates | Not stated | Undergraduate; Postgraduate | Medical education | Not specified | Video recordings of patient-clinician interactions | Not specified | None | Observational skills  Clinical reasoning  Diagnostic accuracy  Collaborative learning  Confidence in clinical skills | Facilitator Observations  Group Discussions  Diagnostic Accuracy Exercises  Feedback Mechanisms  Peer-led Approaches | Patient Video Cases (PVCs) are effective educational tools that enhance clinical reasoning and observational skills by providing real-world scenarios, supporting apprenticeship learning, and fostering collaborative discussions, with recommendations for their focused and ethical use in medical education. |
| (Roy, 2012) [81] | USA | Original Research Paper | Randomised controlled trial | Mixed methods | To investigate preferences for video- or text-based cases and their effects on deep thinking in PBL. | Medical students | Second-year | Undergraduate | Medical education | 172 participants | Video recordings of patient-clinician interactions | 20 - 25 minutes | Text-based case presentation | Depth of thinking (deep vs superficial)  Critical thinking  Student and tutor preferences | Coding Scheme  Generalized Estimating Equation (GEE) Model  Critical Thinking Ratios (CTRs)  Survey | While video-based cases in problem-based learning (PBL) were preferred by most students and tutors for their stimulating and efficient format, the study found they were associated with reduced deep cognitive engagement and critical thinking compared to text-based cases, potentially due to increased cognitive load from the video format. |
| (Scheidt, 1986) [84] | USA | Original Research Paper | Randomised controlled trial | Quantitative | To evaluate the effectiveness of feedback on videotaped performances in teaching interview and examination skills | Medical students | Third-year | Undergraduate | Medical Education | 105 participants | Review of video recordings of patient-clinician interactions | Not specified | Self-guided critique, no feedback | Overall performance  Interview Skills  Examination Skills | Performance Criteria Checklist  Videotaped Encounters  Rating Form  Composite Score Calculation | The study found that preceptor feedback on videotaped patient encounters significantly improved students' interview and examination skills compared to self-critique or no feedback, with notable gains in handling sensitive topics and using hypothesis-driven questioning, underscoring the value of preceptor-led video review in medical education. |
| (Terasaki, 1984) [87] | USA | Original Research Paper | Observational design | Mixed methods | To evaluate the impact of a cancer medicine block on student interactions with cancer patients | Medical students | Second-year | Undergraduate | Medical Education | 32 participants | Review of video recordings of patient-clinician interactions | 5 - 10 minutes | Pre-course patient-clinician interactions | Verbal interactions | Reciprocal Category Analysis (RCA) | The study demonstrated that the use of video recordings of interviews with cancer patients, followed by feedback sessions, significantly improved medical students' communication skills by reducing factual questions and increasing emotional discussions earlier in the interviews. |
| (Tully, 2015) [89] | USA | Original Research Paper | Observational design | Mixed methods | To investigate the utility of Google Glass for recording standardized patient encounters and assess its impact on medical students' learning experiences. | Medical students | Second-year | Undergraduate | Medical Education | 30 participants | First-person video recordings of patient-clinician interactions | 9 - 12 minutes | Static video recordings of simulated patient-clinician interactions | Self-Evaluation of Experience  Perceived Utility of the Intervention  Follow-Up Responses  Comparison of Video Perspectives | Survey | The study found that Google Glass successfully recorded encounters, offering a first-person perspective that traditional cameras couldn't, with the majority of students reporting a positive experience despite 77% finding it distracting at times; 70% of students perceived the technology as helpful for identifying behaviors not noted in traditional video, and 70% supported its inclusion in clinical training, particularly for its unique perspective on nonverbal communication during emotionally challenging interactions. |
| (Yoon, 2016) [92] | South Korea | Original Research Paper | Cross-sectional design | Quantitative | To investigate preclinical students’ experiences with standardized patients (SPs) compared to video cases in problem-based learning (PBL). | Medical students | Second-year | Undergraduate | Medical Education | 99 participants | Video case-based learning | Not specified | Standardised patients | Problem Identification  Hypothesis Generation  Motivation  Collaborative Learning  Reflective Thinking  Authenticity  Patient-Doctor Communication  Attitude Toward Patients | Questionnaire | The study found that standardized patients (SPs) provided a more effective and authentic learning experience than video cases, significantly improving areas such as patient-doctor communication, motivation, reflective thinking, collaborative learning, and students' attitudes toward patients, though challenges were noted in problem identification and hypothesis generation. |
| (Alsalamah, 2023) [29] | Saudi Arabia | Original Research Paper | Phenomenological design | Qualitative | To evaluate perceptions of nursing students regarding video-based simulation. | Nursing students | Not stated | Undergraduate | Nursing Education | 32 participants | Video simulation of patient-clinician encounter | 30 minutes | Face-to-face simulation | Learning and Satisfaction  Communication Skills  Hands-On Experience  Comfort and Anxiety  Technical Barriers | Semi-structured interviews | The study found that video-based simulations enhanced students' communication skills and understanding of patient-clinician interactions, though concerns about the lack of hands-on experience and technical barriers were noted. |
| (Fero, 2010) [42] | USA | Original Research Paper | Quasi-experimental design | Quantitative | To examine the relationship between metrics of critical thinking skills and performance in simulated clinical scenarios. | Nursing students | Not stated | Undergraduate | Nursing education | 36 participants | Video simulation of patient-clinician encounter | 10 minutes | High-fidelity human simulation | Performance rating  Critical thinking skills | California Critical Thinking Disposition Inventory (CCTDI)  California Critical Thinking Skills Test (CCTST)  Videotaped Vignette/High-Fidelity Human Simulation Assessment Tool | The study found that most nursing students did not meet performance expectations in both videotaped vignettes and high-fidelity human simulation, with no significant difference between the two methods, though students performed better in initiating nursing interventions with HFHS, and a strong critical thinking disposition was linked to better HFHS performance, highlighting the need for improved teaching strategies to enhance clinical application of critical thinking skills. |
| (Forbes, 2016) [44] | Australia | Original Research Paper | Observational design | Quantitative | To investigate the feasibility of head-mounted video camera recordings to augment feedback following acute patient deterioration simulations. | Nursing students | Third-year | Undergraduate | Nursing education | 10 participants | Review of video recordings of patient-clinician interactions | Not specified | Verbal feedback | Participant performance (observed and perceived)  Perceived confidence and competence  Perceptions of feedback methods  Feasibility and capability of head-mounted video camera recordings for detailed audio-visual feedback | Observational Data Collection Tool (adaptation of the Gaba's Clinical Simulation Tool)  Evaluation Tool for Feedback Sessions | The study found mixed perceptions of video feedback, with some participants finding it beneficial for learning, while others felt it was ineffective, and technical limitations of the head-mounted cameras, such as misalignment with the participants' field of vision, impacted the usefulness of the video recordings. |
| (Minardi, 1999) [64] | UK | Original Research Paper | Comparative design | Qualitative | To evaluate the efficacy of video recording in enhancing interpersonal skills in nursing education. | Nursing students | Second-year; Third-year; Fourth-year | Undergraduate | Nursing education | 77 participants | Video recordings of patient-clinician interactions | Not specified | None | Categorisation of statements | Questionnaire  Categorisation framework | The study found that a majority of participants viewed video recording as a beneficial tool for enhancing interpersonal skills during clinical supervision, with statistical analysis revealing significant differences in perceptions across groups, despite some concerns about anxiety, and emphasized the need for further research on the long-term effects of video-based learning. |
| (Nunohara, 2020) [70] | Japan | Original Research Paper | Quasi-experimental design | Qualitative | To explore the influence of video and paper case modalities on clinical decision-making processes of midwifery students | Midwifery students | Not stated | Postgraduate | Nursing Education | 45 participants | Video case-based learning | Not specified | Paper case-based learning | Clinical decision-making  Clinical procedures  Psychosocial vs. Biomedical focus  Empathy and Patient-Centeredness | Three-Stage Model of Clinical Decision-Making  Frequency Analysis  Qualitative Content Analysis | The study found that video case learning promoted a more woman- and family-centered approach, emphasizing psychosocial care and empathy, while paper case learning fostered a healthcare provider-centered, biomedical perspective, with students in the video group prioritizing patient comfort over invasive procedures and using more empathetic language. |
| (Nyström, 2014) [71] | Sweden | Original Research Paper | Descriptive design | Qualitative | To describe bachelor nursing students’ experiences of being video-recorded during an examination with a simulated patient in emergency care. | Nursing students | Not stated | Undergraduate | Nursing education | 44 participants | Video recording of simulated patient-clinician interactions | Not specified | Traditional methods | Nervousness and Apprehension  Dialogue and Acknowledgement  Self-Knowledge and Professional Growth  Effectiveness of the intervention | Open-ended questions | The study found that video-recording during clinical exams initially made nursing students nervous, but ultimately helped them engage in valuable feedback discussions, leading to greater self-awareness, improved clinical skills, and confidence in patient-clinician interactions. |
| (Thomas, 2023) [88] | USA | Original Research Paper | Quasi-experimental design | Mixed methods | To expose prelicensure nursing students to virtual patient encounters via telenursing. | Nursing students | Not stated | Undergraduate | Nursing Education | 47 participants | Video simulation of patient-clinician encounter | Not specified | No intervention | Examination Scores  Student feedback (communication and patient education skills) | Content-Related Examination Questions  Debriefing Feedback | The simulated telenursing intervention led to significantly higher examination scores for participating students, who also reported increased confidence in patient interviewing, health history taking, and patient education skills; faculty and standardized patient feedback further supported student engagement, highlighting the potential of telenursing as a viable solution to clinical placement shortages and underscoring the need for continued research into its long-term educational benefits. |
| (Vessey, 2002) [90] | USA | Original Research Paper | Quasi-experimental design | Mixed methods | To evaluate the effectiveness of standardized patient encounters for clinical assessment | Nursing students | Not stated | Master | Nursing Education | 26 participants | Video simulation of patient-clinician encounter | 30 minutes | Not specified | Correctness of Differential Diagnosis  Thoroughness of Clinical Examination  Performance | Checklist | The study found that while simulated clinical encounters (SCEs) provided valuable learning experiences for nurse practitioner students, their performance in SCEs did not align with other clinical evaluations, highlighting discrepancies and suggesting that SCEs should be used as formative assessments rather than sole evaluative measures due to concerns about reliability and student anxiety. |
| (Parlak Özer, 2024) [74] | Turkey | Original Research Paper | Quasi-experimental design | Qualitative | To describe the professional development process of student dietitians through video-mediated communication skills training, with a focus on patient counseling. | Dietetic students | Not stated | Undergraduate | Nutrition and Dietetics Education | 12 participants | Video simulation of patient-clinician encounter | Not specified | Traditional methods | Listenership Skills  Interactional competence  Patient counseling competence  Feedback from patients | Conversation Analysis  Interviews  Written reflections | The study demonstrated that video-mediated communication training, supported by structured feedback and reflective practices, significantly improved a student dietitian's counseling skills, with enhancements in listenership and interactional techniques that effectively transferred to real patient sessions, receiving positive feedback from patients and instructors. |
| (Giles, 2014) [45] | USA | Original Research Paper | Quasi-experimental design | Mixed methods | To assess occupational therapy students’ preparedness for Level II fieldwork using simulated patients and reflective video analysis. | Occupational therapy students | Second-year | Master | Occupational Therapy Education | 88 participants | Review of video recordings of patient-clinician interactions | Not specified | None | Student Performance on the Comprehensive Practical Exam (CPE)  Student Perceptions of the CPE  Self-Improvement Plans  Feedback | Comprehensive Practical Exam (CPE) Score Sheet  Reflective Video Analysis Worksheet  Post-CPE Feedback Surveys  Thematic Content Analysis | The study found the Comprehensive Practical Exam (CPE), which included simulated patient encounters and reflective video analysis, to be an effective tool for assessing students' preparedness for Level II fieldwork. Students reported that it enhanced clinical preparation, confidence, and self-assessment abilities. They appreciated the realism of the simulated encounters, valued feedback from evaluators, and suggested improvements such as reducing anxiety, enhancing video quality, and increasing simulation opportunities throughout the curriculum. |
| (Murphy, 2018) [66] | USA | Original Research Paper | Quasi-experimental design | Quantitative | To examine the effect of video cases on the development of clinical reasoning skills in occupational therapy students. | Occupational therapy students | First-year | Undergraduate | Occupational Therapy Education | 61 participants | Video recordings of patient-clinician interactions | Not specified | Text-based case presentation | Clinical reasoning | Health Science Reasoning Test (HSRT) | The study found that students who engaged with online video cases combined with clinical reasoning activities showed significant improvements in clinical reasoning skills, particularly in inductive reasoning; while the text-based case study group also improved, their gains were not statistically significant, highlighting the potential benefits of video case studies for enhancing clinical reasoning in educational curricula. |
| (Rodríguez-Bailón, 2021) [79] | Spain | Original Research Paper | Randomised controlled trial | Mixed methods | To compare the effect of video cases vs. paper cases on motivation and clinical reasoning | Occupational therapy students | Not stated | Undergraduate | Occupational Therapy education | 120 participants | Video recording of simulated patient-clinician interactions | 12 minutes | Paper case-based learning | Motivation for learning  Clinical reasoning | Instructional Material Motivation Survey (IMMS)  Clinical Reasoning Case Exercise | Students who engaged with video cases involving simulated patient interactions reported higher motivation for learning, emphasizing the realism, empathy, and engagement fostered by the format, while also identifying challenges in extracting specific details compared to paper cases, suggesting that video cases provide unique benefits for understanding patient contexts and professional roles. |
| (Temple, 2022) [86] | USA | Original Research Paper | Quasi-experimental design | Mixed methods | To assess the practical value of video cases as an educational tool for OT students before Level II fieldwork. | Occupational Therapy Students | Not stated | Doctorate | Occupational Therapy Education | 25 participants | Video case-based learning | Not specified | Not specified | Preparedness for Fieldwork  Learning tool preferences | Survey  Interviews | Simulated patient interactions and video case studies were effective in preparing occupational therapy students for Level II fieldwork, with most students reporting adequate preparedness, though qualitative feedback highlighted the need for more hands-on practice, particularly in toileting skills, to address gaps in readiness. |
| (Bethea, 2019) [33] | USA | Original Research Paper | Quasi-experimental design | Quantitative | To examine the impact of video-based and live standardized patient scenarios on attitudes toward and readiness for interprofessional education (IPE) in OT and PT students. | Occupational therapy students; Physiotherapy students | Not stated | Undergraduate | Interprofessional Education (IPE) in healthcare | 49 participants | Video recording of simulated patient-clinician interactions | Not specified | Live standartized patient scenarios (SPSE) | Teamwork  Professional identity | Readiness for Inter-Professional Learning Scale (RIPLS) | The study found that live standardized patient scenarios significantly improved attitudes and readiness for interprofessional education among occupational therapy and physical therapy students, with notable statistical improvements in the Teamwork and Professional Identity subscales of the Readiness for Inter-Professional Learning Scale, highlighting the value of live, interactive scenarios over video-based ones in fostering interprofessional teamwork skills essential for healthcare delivery. |
| (Bessette, 2021) [32] | Canada | Original Research Paper | Observational design | Qualitative | To develop an educational activity to enhance self-awareness in consultation skills and assess learner perceptions. | Pharmacy students and pharmacy residents | Fourth-year; Graduated | Undergraduate; Postgraduate | Pharmacy Education | 8 participants | Video recordings of patient-clinician interactions | 60 minutes | Not specified | Self-Awareness of Competencies  Identification of Areas for Improvement  Perceived Benefit of the Learning Activity | Self-Assessment Forms, based on the Comprehensive Medication Management (CMM)  Online Feedback Survey  Assessment Forms Completed by Preceptors | The study demonstrated that using video recordings for patient consultation training significantly enhanced pharmacy learners' self-awareness of their competencies and skills, with learners identifying improvements in areas such as questioning, language clarity, time management, and non-verbal communication, while fostering alignment between self-assessments and preceptor evaluations. |
| (Flood, 2019) [43] | Ireland | Original Research Paper | Case study design | Quantitative | To develop video-based case studies for authentic assessments in pharmacy education | Pharmacy students | Graduated | Master | Pharmacy education | Not specified | Video recording of simulated patient-clinician interactions | 5 minutes | Not applicable | Perceived effectiveness in demonstrating practical application of knowledge and skills  Realism of the case presentations  Ability to identify characters' emotions | Survey | The study found that video recordings of simulated patient-clinician interactions were effective in postgraduate pharmacy education, with students reporting that the cases facilitated practical application of knowledge, were presented realistically, and supported the identification of characters' emotions, highlighting their value as an engaging and authentic assessment tool. |
| (Raja, 2008) [76] | India | Original Research Paper | Randomised controlled trial | Mixed methods | To evaluate the effectiveness of video-based teaching over conventional bedside teaching in undergraduate students. | Physiotherapy students | Not stated | Undergraduate | Physiotherapy education | 200 participants | Video recordings of patient-clinician interactions | 15 - 18 minutes | Live patient-clinician interactions | Learning outcomes  Test scores  Learning styles | Objective Type Questions  Follow-Up Evaluation  Learning Styles Questionnaire | The study founf that video-based teaching significantly improved learning outcomes, retention, and understanding of complex concepts compared to conventional bedside teaching, with students expressing higher satisfaction and better retention of knowledge over time, especially for challenging topics like cerebral palsy. |
| (Hammarström, 2021) [48] | Sweden | Original Research Paper | Observational design | Qualitative | To investigate whether video recordings of interactions involving persons with aphasia and speech-language pathology students enhance students’ competence as conversation partners and explore experiences from both perspectives. | Speech Pathology students | Not stated | Undergraduate | Speech-language therapy education | 34 participants | Video recordings of patient-clinician interactions | 10 minutes | None | Student Competence as Conversation Partners  Knowledge Acquisition  Experiences and Perspectives of PWAs  Interactional Strategies  Student Self-Reflection | Analysis of video recordings  Post-interaction questionnaire  Semi-structured focus-group interview | The study found that video-recorded interactions with persons with aphasia (PWAs) significantly improved students' communication competence, enhanced their understanding of aphasia's impact, highlighted effective and less effective interactional strategies, and demonstrated the educational and empowering value of involving PWAs as experts by experience. |
| (Lewis, 2015) [60] | Australia | Original Research Paper | Descriptive design | Mixed methods | To evaluate student responses to a peer review activity where students used video recordings of their interactions with clients to engage in reflective practice and peer feedback. | Speech Pathology students | Third-year | Undergraduate | Speech-language therapy education | 19 participants | Review of video recordings of patient-clinician interactions | < 2 minutes | Not applicable | Feasibility  Usefulness for learning  Emotional responses  Overall satisfaction | Questionnaires  Open-ended questions  Thematic analysis | The study found that the peer review activity using video recordings was generally feasible and beneficial for learning, with students appreciating clear instructions, peer feedback, and the opportunity to reflect on clinical skills; however, connections between theory and practice were less evident to participants, emotional responses were largely neutral with low stress or anxiety reported, and while satisfaction was high, only half of the students expressed a desire to repeat the activity, suggesting the need for deeper guidance on reflection and the incorporation of constructive criticism. |
| (Oosthuizen, 2019) [73] | South Africa | Original Research Paper | Phenomenological design | Qualitative | To describe the perceptions of undergraduate students regarding the inclusion of authentic video cases in a theoretical module on developmental communication disorders. | Speech Therapy students; Hearing therapy students | Second-year | Undergraduate | Speech-language therapy education | 22 participants | Video recordings of patient-clinician interactions | 8 - 34 minutes | Not applicable | Understanding and Engagement  Motivation and Relevance  Cognitive Load  Awareness of Clinical Populations  Reflection on Skills Needed | Focus-Group Interviews  Adaptive Interview Schedule  Contextualized Content Analysis | The key findings of the study revealed that authentic video cases enhanced students' understanding and engagement with theoretical content, increased their motivation by showcasing real-life communication challenges, and improved their awareness of clinical populations; however, some students experienced cognitive overload, highlighting the need for careful management of multimedia content, while participants also reflected on the interpersonal skills required for effective therapy practice. |
| (Hafen, 2015) [47] | USA | Original Research Paper | Descriptive design | Mixed methods | To evaluate third-year veterinary students' perceptions of a communication lab protocol using video clips of authentic client interactions. | Veterinary medical students | Third-year | Undergraduate | Veterinary Medical Education | 108 participants | Video recordings of patient-clinician interactions | Not specified | None | Levels of interest in communication skills  Knowledge acquisition  Perceived future helpfulnes of communication skills  Feedback on teaching interventions  Engagement | Survey  Open-ended feedback questions | The key findings from the study indicate that the communication lab, particularly the use of authentic client interaction videos, significantly increased third-year veterinary students' interest in communication skills, enhanced their knowledge, and improved their perceptions of the relevance of these skills for their future careers, with particularly strong engagement from initially disinterested students. |
| (Hafen, 2013) [46] | USA | Original Research Paper | Observational design | Quantitative | To evaluate the effectiveness of communication skills training using video feedback. | Veterinary medical students | Fourth-year | Undergraduate | Veterinary Medical Education | 415 participants | Video recordings of patient-clinician interactions | 45 minutes | None | Communication skills  Client feedback  Self-assessment of skills | Questionnaire  Researcher-developed rating form | The key findings from the study are as follows: Communication skills improved significantly across several areas, with notable increases in discussing findings, nonverbal communication, and asking questions; baseline scores in specific skills predicted overall performance; attention to communication goals during feedback sessions predicted final scores, explaining a significant portion of the variance; the most recent cohort showed the highest scores, suggesting improvements in the training protocol over time; students perceived the training as valuable for their future careers; and the study suggests long-term benefits in communication proficiency. |
| (Hafen, 2009) [94] | USA | Original Research Paper | Observational design | Qualitative | To present a communication training protocol utilizing filmed student-client interactions. | Veterinary medical students | Not stated | Undergraduate | Veterinary Medical Education | 77 participants | Review of video recordings of patient-clinician interactions | 45 minutes | None | Communication skills  Self-awareness and personal growth  Feedback | Video review form  Student rating  Written feedback | The key findings of the study include positive student perception of the communication training, increased self-awareness about communication styles, the effectiveness of private feedback sessions, identification of common areas for improvement (e.g., eye contact, empathy), and the value of filmed interactions as a tool for enhancing communication skills, with a recommendation for further research to evaluate its broader application in veterinary education. |
| (Bowles, 2020) [35] | USA | Original Research Paper | Observational design | Qualitative | To analyze dental student conversations about patient treatment plans with native English and English as Second Language (ESL) patients. | Dental students | Fourth-year | Undergraduate | Dental Education | 8 participants | Video recording of patient-clinician interactions | 30 minutes | None | Communication errors  Conversational Dynamics  Completion of the Three-Part Medical Discourse Set (TPMDS) | Transcription of discussions  Questionnaires  Linguistic and Sociolinguistic Factors | Dental students made three common errors with ESL patients: assumption of comprehension, use of technical jargon, and lack of multimodal communication strategies. |
| (Chi, 2014) [37] | USA | Original Research Paper | Observational design | Mixed methods | To compare learning outcomes associated with video cases versus paper cases in an introductory public health dentistry course | Dental students | Not stated | Undergraduate | Dental Education | 247 participants | Video recording of simulated patient-clinician interactions | Not specified | Paper case-based learning | Cognitive learning outcomes (knowledge and understanding)  Affective learning outcomes (Engagement, empathy, and critical thinking)  Overall learning outcomes | Survey  Open-ended questions | Students using video cases reported significantly better cognitive and affective learning outcomes compared to those using paper cases, with higher mean scores across all measures (e.g., empathy, problem-solving, critical thinking). Video cases enhanced engagement and relatability to the material. |
| (Davies, 2017) [39] | UK | Original Research Paper | Descriptive design | Qualitative | To explore the use of video vignettes to enhance reflective learning among dental undergraduates. | Dental students | Fifth-year | Undergraduate | Dental Education | 20 participants | Review of video recordings of patient-clinician interactions | < 3 minutes | None | Reflection on Video  Reinforcement of Learning  Sharing Clinical Experiences  Enhancement of Total Patient Care Learning  Quality of Reflection | Questionnaire  Conversational analysis | Dental students made three common errors with ESL patients: assumption of comprehension, use of technical jargon, and lack of multimodal communication strategies. |
| (Edrees, 2014) [40] | Sweden | Original Research Paper | Observational design | Quantitative | To assess the perceived benefits of video-mediated demonstrations in learning endodontics. | Dental students | Third-year | Undergraduate | Dental Education | 75 participants | Video-mediated demonstration of patient-clinician interactions | 120 minutes | None | Perceived Benefits in Communication Skills  Perceived Benefits in Diagnosis:  Perceived Benefits in Treatment Procedures | Questionnaire | The study found that video-mediated demonstrations significantly enhanced the perceived benefits of learning endodontic treatment procedures among third-year dental students, with a notable difference in perceived value between the first and second video sessions, particularly in communication and treatment procedures. |
| (Janda, 2004) [51] | Sweden | Original Research Paper | Randomised controlled trial | Mixed methods | To evaluate the design, usability, and learning effect of a virtual patient in improving history-taking skills among dental students. | Dental students | Second-year | Undergraduate | Dental Education | 39 participants | Video recording of simulated patient-clinician interactions | 20 minutes | Traditional methods | Time spent  Critical questions  Professional Behavior | Usability Test  Evaluation Criteria | Students who practiced with the virtual patient asked more relevant questions, spent more time on patient issues, and displayed greater empathy compared to those who only received standard instruction. The virtual patient training significantly improved the students' history-taking skills. |
| (Kalwitzki, 2005) [54] | Germany | Original Research Paper | Quasi-experimental design | Quantitative | To evaluate the aspects of clinical behaviour that changed after video-based teaching, particularly focusing on practitioner-patient interaction. | Dental students | Fifth-year | Undergraduate | Dental Education | 113 participants | Review of video recordings of patient-clinician interactions | 10 minutes | None | Verbal and non-verbal communication  Confidence in dealing with patients Handling of patients with fear or pain  Ergonomics | Questionnaire | Significant self-reported changes in clinical behaviour were observed, particularly in communication skills and confidence levels. A high percentage of students attributed these changes to the video sequences, indicating the effectiveness of video in dental education. |
| (Miller, 2015) [63] | USA | Original Research Paper | Quasi-experimental design | Mixed methods | To evaluate the effectiveness of clinical scenario videos in improving dental students' perceptions of basic sciences and their ability to apply content knowledge. | Dental students | First-year | Undergraduate | Dental Education | 120 participants | Review of video recordings of simulated patient-clinician interactions | 13 - 15 minutes | Traditional methods | Student performance  Perceptions | Questionnaire | The use of clinical scenario videos significantly improved students' ability to apply physiological concepts to clinical situations, increased exam scores on clinically related questions by 6.2%, and enhanced students' perceptions of the relevance of physiology to their future dental careers. |
| (Omar, 2021) [72] | USA | Original Research Paper | Non-experimental design | Mixed methods | To ensure that learning objectives related to standardized patient encounters were achieved during the COVID-19 pandemic | Dental students | First-year; Second-year | Undergraduate | Dental Education | 101 participants | Review of video recordings of simulated patient-clinician interactions | Not specified | Traditional methods | Student performance  Feedback from SPs  Self-evaluation  Faculty evaluation | Feedback sessions  Questionnaire | The web-based platform allowed successful completion of SP encounters, provided timely feedback, and was positively received by students despite some technical issues. It enhanced communication skills in preclinical years during the pandemic. |
| (Quinn, 2015) [75] | UK | Original Research Paper | Case study design | Qualitative | To examine dentists’ views of a novel video review technique to improve communication skills in complex clinical situations. | Dentists | Graduated | Not applicable | Dental Education | 3 participants | Review of video recordings of patient-clinician interactions | 35 minutes | Traditional methods | Reflection on their communication strategies  Awareness of non-verbal communication Perceived benefits of intervention | Checklist  Thematic analysis | The video sessions were perceived positively by dentists, who reported increased awareness of their communication strategies and the importance of non-verbal cues. The approach was seen as beneficial for training and improving interactions with patients, particularly those with complex communication needs |
| (Reher, 2020) [77] | Australia | Original Research Paper | Quasi-experimental design | Mixed methods | To evaluate the impact of video recording and self-reflection on the communication skills training of first-year dental students | Dental students | First-year | Undergraduate | Dental Education | 312 participants | Review of video recordings of simulated patient-clinician interactions | Not specified | Traditional methods | Student engagement  Communication skills development  Satisfaction with assessments  Knowledge | Student Evaluation of Courses (SEC) Data  Test | The introduction of video recording and self-reflection significantly improved student engagement, satisfaction with assessments, and final practical exam grades. |
| (Sanders, 2008) [82] | USA | Original Research Paper | Quasi-experimental design | Quantitative | To evaluate the effectiveness of an interactive, virtual-patient module in improving dental students' comfort and knowledge regarding care for patients with developmental disabilities | Dental students | Third-year | Undergraduate | Dental Education | 44 participants | Review of video recordings of simulated patient-clinician interactions | Not specified | None | Confort levels  Knowledge | Disability Situations Inventory (DSI)  Knowledge test | Significant improvements in students' perceived comfort and knowledge levels after completing the module, with participants expressing overall satisfaction with the learning experience. |
| (Sanderson, 2016) [83] | USA | Original Research Paper | Experimental design | Quantitative | To determine if the use of a video-recorded clinical session affects the accuracy of dental hygiene student self-assessment and dental hygiene instructor feedback | Dental hygiene students | First-year | Undergraduate | Dental Education | 32 participants | Review of video recordings of simulated patient-clinician interactions | Not specified | Traditional methods | Student self-assessment  Instructor assessment  Expert assessment | Assessment rubric for clinical performance | The study found that while students' self-assessment scores increased after video review, instructor scores tended to decrease, indicating a potential for improved calibration and assessment accuracy through video feedback. No significant differences were found between overall scores, but discrepancies were noted in grading criteria compared to expert assessments. |
| (Zahl, 2016) [93] | USA | Original Research Paper | Comparative design | Mixed methods | To evaluate student perceptions of their ability to self and peer assess interpersonal communication skills and clinical procedures during standardized patient interactions recorded by Google Glass compared to a static camera | Dental students | Third-year | Undergraduate | Dental Education | 30 participants | First-person video recording of simulated patient-clinician interactions | Not specified | Static video recordings of simulated patient-clinician interactions | Interpersonal Communication Skills  Clinical Procedures | Video Review Assessment Effectiveness Scale (VRAES) | Students perceived first-person recording as more effective for assessing verbal and paraverbal communication skills, while static recordings were deemed more effective for assessing nonverbal communication. Emergent themes included the advantages of first-person perspective and audiovisual quality, alongside challenges related to the limited viewing area and head movements during recording. |
| (Schwartz, 2012) [85] | Canada | Original Research Paper | Quasi-experimental design | Mixed methods | To examine the impact of integrating patient videos into dental education on students' professionalism and empathy. | Dental students | Second-year; Third-year | Undergraduate | Dental Education | 87 participants | Video recordings of patient-clinician interactions | Not specified | Traditional methods | Empathy  Professionalism  Effectiveness of intervention | Surveys (including the Jefferson Scale of Empathy)  Reflective journals | The integration of patient videos significantly enhanced students' educational experiences, increased their commitment to professionalism, and improved their empathy levels, particularly among second-year students. The majority of students found the videos memorable and beneficial for learning patient-centered care. |
| (White, 2008) [91] | South Africa | Original Research Paper | Quasi-experimental design | Mixed methods | To develop and implement a course in communication skills for third-year dental students. | Dental students | Third-year | Undergraduate | Dental Education | 67 participants | Review of video recordings of simulated patient-clinician interactions | Not specified | Traditional methods | Communication skills | Assessment rubric  Questionnaire | The study found significant improvement in students' communication skills from the first training cycle to the second. Students emphasized the importance of trust, empathy, and active listening in establishing meaningful relationships with patients. |
