# Supplementary material for: Video Recording of Patient-Clinician Interactions in Health Education: Scoping Review
Source: JMIR Med Educ. 2026 Jul 13;12:e70324. doi: 10.2196/70324 (PMC13361625; doi:10.2196/70324)
Supplement: Multimedia Appendix 3 [file mededu-v12-e70324-s003.docx]

| **Term** | **Definition** |
| --- | --- |
| Author and Year | The name of the primary author and the year of publication. |
| Origin of the Study | The geographical location at which the study was conducted and the location from which the data were collected. |
| Type of Article | The classification of an article based on its purpose and content. |
| Research Design | The methodology employed in the study. |
| Data Type | The specific data types that were collected or analysed in the study. |
| Aims/Purpose | The specific objectives or goals of the study, as set forth by the authors. |
| Student Population | The characteristics of the student participants in the study. |
| Sample Size | The total number of participants included in the study. |
| Intervention Type | The specific type of intervention that is the subject of the study. |
| Duration | The length of time over which the intervention occurred. |
| Comparator | The control or comparison condition used in the study, if applicable. |
| Outcome Variables | The key variables measured in order evaluate the outcomes of the intervention or study. |
| Instruments | The instruments or techniques employed to gather information pertaining to the variables of interest. |
| Patient-Clinician Interactions | The term refers to the utilisation of authentic interactions between patients and healthcare professionals, as well as allied health practitioners, that occur within the context of clinical or healthcare settings. In essence, it represents the transfer of information and the delivery of care between a patient and a healthcare professional.  *Synonyms: Live patient encounters, in-person patient consultations, direct patient-provider interactions, face-to-face patient encounters, real-time patient engagements* |
| Simulated Patient-Clinician Interactions | The utilisation of structured, controlled scenarios that replicate authentic patient-clinician interactions. Such scenarios may utilise the services of standardised patients (trained actors) or virtual simulations.  *Synonyms: Standardized patient encounters, simulated clinical scenarios* |
