# Supplementary material for: Video Recording of Patient-Clinician Interactions in Health Education: Scoping Review
Source: JMIR Med Educ. 2026 Jul 13;12:e70324. doi: 10.2196/70324 (PMC13361625; doi:10.2196/70324)
Supplement: Multimedia Appendix 4 [file mededu-v12-e70324-s004.docx]

| **(Author, Year)** | **Category** | **Subcategory** | **General Outcome Variable** | **Specific Outcome Variable** |
| --- | --- | --- | --- | --- |
| (Ahmet, 2018) [28] | Clinical Competence | Clinical Skills | Technical and non-technical skills | Technical Skills |
|  | Clinical Competence | Performance | Overall performance | Overall performance |
|  | Critical Thinking and Reasoning | Cognitive Processes | Knowledge retention | Knowledge retention and understanding |
|  | Critical Thinking and Reasoning | Cognitive Processes | Long-term retention | Long-term retention of Skills |
|  | Educational Process | Learning Process | Effectiveness of the learning process | Effectiveness of the learning process |
|  | Engagement and Learning Experience | Learner Engagement | Learner satisfaction | Trainee Satisfaction and Comfort |
| (Alsalamah, 2023) [29] | Communication Skills | Verbal and Non-Verbal Communication | Communication skills | Communication Skills |
|  | Emotional and Psychological Outcomes | Emotional Impact | Emotional responses | Comfort and Anxiety |
|  | Engagement and Learning Experience | Learner Engagement | Learning and satisfaction | Learning and Satisfaction |
|  | Engagement and Learning Experience | Motivation | Barriers to learning | Technical Barriers |
|  | Learning Outcomes and Retention | Learning Assessment | Hands-on experience | Hands-On Experience |
| (Balslev, 2005) [30] | Critical Thinking and Reasoning | Cognitive Processes | Critical thinking | Frequency of different types of clauses generated by participants during verbal interactions (Data exploration, Theory building, Theory evaluation, Metareasoning) |
| (Batteson, 2023) [31] | Communication Skills | Patient Interaction | Awareness of clinical populations | Identification of Social Determinants of Health |
|  | Feedback and Assessment | Evaluation | Assessment of learning outcomes | Learning Assessment |
|  | Teamwork and Interprofessional Learning | Team Dynamics | Interprofessional roles and competencies | Interprofessional competencies (teamwork, communication, roles and responsibilities, values and ethics) |
| (Bessette, 2021) [32] | Critical Thinking and Reasoning | Reflection | Self-awareness | Self-Awareness of Competencies |
|  | Critical Thinking and Reasoning | Reflection | Self-reflection and improvement | Identification of Areas for Improvement |
|  | Patient and Clinical Context | Patient-Centered Care | Perceived relevance | Perceived Benefit of the Learning Activity |
| (Bethea, 2019) [33] | Emotional and Psychological Outcomes | Personal Growth | Professional identity | Professional identity |
|  | Teamwork and Interprofessional Learning | Team Dynamics | Teamwork and collaboration | Teamwork |
| (Botelho, 2016) [34] | Communication Skills | Reflection on Communication | Self-reflection | Self-Reflection and Peer Feedback |
|  | Engagement and Learning Experience | Learner Engagement | Engagement | Engagement with Consultation Videos |
|  | Engagement and Learning Experience | Learning Environment | Feasibility | Technical, Practical, and Logistical Feasibility |
|  | Feedback and Assessment | Feedback Methods | Feedback effectiveness | Student Feedback |
| (Bowles, 2020) [35] | Communication Skills | Verbal and Non-Verbal Communication | Communication skills | Completion of the Three-Part Medical Discourse Set (TPMDS) |
|  | Communication Skills | Verbal and Non-Verbal Communication | Communication challenges | Communication errors |
|  | Communication Skills | Verbal and Non-Verbal Communication | Communication skills | Conversational Dynamics |
| (Chan, 2010) [36] | Clinical Competence | Clinical Skills | Application of learned skills | Integration of Learning |
|  | Clinical Competence | Clinical Skills | Clinical reasoning | Clinical reasoning and observational skills |
|  | Emotional and Psychological Outcomes | Personal Growth | Self-directed learning | Self-Directed Learning |
|  | Engagement and Learning Experience | Learner Engagement | Student engagement | Engagement and interest |
| (Chi, 2014) [37] | Emotional and Psychological Outcomes | Emotional Impact | Affective outcomes | Affective learning outcomes (Engagement, empathy, and critical thinking) |
|  | Learning Outcomes and Retention | Learning Assessment | Learning outcomes | Cognitive learning outcomes (knowledge and understanding) |
|  | Learning Outcomes and Retention | Learning Assessment | Learning outcomes | Overall learning outcomes |
| (Courteille, 2014) [38] | Emotional and Psychological Outcomes | Emotional Impact | Affective learning outcomes | Affective Learning Outcomes |
|  | Emotional and Psychological Outcomes | Personal Growth | Self-reported measures | Self-Reported Measures |
|  | Learning Outcomes and Retention | Learning Assessment | Log Activity Data | Log Activity Data |
|  | Teamwork and Interprofessional Learning | Team Dynamics | Behavioral Analysis | Behavioral Analysis |
| (Davies, 2017) [39] | Clinical Competence | Clinical Skills | Clinical experience sharing | Sharing Clinical Experiences |
|  | Communication Skills | Patient Interaction | Patient care learning | Enhancement of Total Patient Care Learning |
|  | Communication Skills | Reflection on Communication | Self-reflection | Reflection on Video |
|  | Communication Skills | Reflection on Communication | Self-reflection | Quality of Reflection |
|  | Learning Outcomes and Retention | Learning Assessment | Learning reinforcement | Reinforcement of Learning |
| (Edrees, 2014) [40] | Communication Skills | Verbal and Non-Verbal Communication | Perceived benefits of communication skills | Perceived Benefits in Communication Skills |
|  | Patient and Clinical Context | Patient-Centered Care | Perceived benefits of role understanding | Perceived Benefits in Role Understanding |
| (Farnan, 2013) [41] | Educational Process | Learning Process | Effectiveness of the interactive materials | Effectiveness of the interactive materials |
|  | Engagement and Learning Experience | Learner Engagement | Student engagement | Participant engagement |
|  | Engagement and Learning Experience | Motivation | Intent to change behavior | Intent to change behavior |
|  | Patient and Clinical Context | Patient-Centered Care | Perceived relevance | Perception of Usefulness |
|  | Practical Application of Knowledge | Educational Tools | Perception of video realism | Perception of video realism |
| (Fero, 2010) [42] | Clinical Competence | Performance | Performance assessment | Performance rating |
|  | Critical Thinking and Reasoning | Cognitive Processes | Critical thinking | Critical thinking skills |
| (Flood, 2019) [43] | Clinical Competence | Clinical Skills | Practical application of knowledge | Perceived effectiveness in demonstrating practical application of knowledge and skills |
|  | Engagement and Learning Experience | Learning Environment | Authenticity and realism | Realism of the case presentations |
|  | Engagement and Learning Experience | Learning Environment | Emotional intelligence | Ability to identify characters' emotions |
| (Forbes, 2016) [44] | Clinical Competence | Performance | Confidence and competence | Perceived confidence and competence |
|  | Clinical Competence | Performance | Performance assessment | Participant performance (observed and perceived) |
|  | Engagement and Learning Experience | Learning Environment | Feasibility | Feasibility and capability of head-mounted video camera recordings for detailed audio-visual feedback |
|  | Feedback and Assessment | Feedback Methods | Feedback effectiveness | Perceptions of feedback methods |
| (Giles, 2014) [45] | Clinical Competence | Performance | Performance assessment | Student Performance on the Comprehensive Practical Exam (CPE) |
|  | Critical Thinking and Reasoning | Reflection | Self-reflection and improvement | Self-Improvement Plans |
|  | Engagement and Learning Experience | Learner Engagement | Learner perceptions | Student Perceptions of the CPE |
|  | Feedback and Assessment | Feedback Methods | Feedback effectiveness | Feedback |
| (Hafen, 2013) [46] | Communication Skills | Verbal and Non-Verbal Communication | Communication skills | Communication skills |
|  | Feedback and Assessment | Evaluation | Self-assessment | Self-assessment of skills |
|  | Feedback and Assessment | Feedback Methods | Feedback effectiveness | Client feedback |
| (Hafen, 2015) [47] | Communication Skills | Verbal and Non-Verbal Communication | Perceived relevance of communication skills | Perceived future helpfulness of communication skills |
|  | Critical Thinking and Reasoning | Cognitive Processes | Knowledge acquisition | Knowledge acquisition |
|  | Engagement and Learning Experience | Learner Engagement | Engagement | Engagement |
|  | Engagement and Learning Experience | Learner Engagement | Engagement | Levels of interest in communication skills |
|  | Feedback and Assessment | Feedback Methods | Feedback effectiveness | Feedback on teaching interventions |
| (Hammarström, 2021) [48] | Communication Skills | Patient Interaction | Patient interaction and care | Experiences and Perspectives of PWAs |
|  | Communication Skills | Reflection on Communication | Self-reflection | Student Self-Reflection |
|  | Communication Skills | Verbal and Non-Verbal Communication | Communication skills | Student Competence as Conversation Partners |
|  | Communication Skills | Verbal and Non-Verbal Communication | Communication strategies | Interactional Strategies |
|  | Critical Thinking and Reasoning | Cognitive Processes | Knowledge acquisition | Knowledge Acquisition |
| (Hammoud, 2012) [49] | Communication Skills | Verbal and Non-Verbal Communication | Communication behaviors | Communication skills |
|  | Engagement and Learning Experience | Learner Engagement | Learner satisfaction | Student satisfaction |
|  | Feedback and Assessment | Evaluation | Self-assessment | Self-Assessment challenges |
|  | Feedback and Assessment | Feedback Methods | Feedback effectiveness | Effectiveness of expert feedback |
| (Henry, 2012) [50] | Emotional and Psychological Outcomes | Emotional Impact | Emotional responses | Participants' Thoughts, Beliefs, and Emotions |
|  | Emotional and Psychological Outcomes | Emotional Impact | Emotional responses | Participants' physiological or emotional responses |
| (Henry, 2020) [20] | Communication Skills | Patient Interaction | Patient-centeredness | Patient and clinician experiences |
|  | Communication Skills | Verbal and Non-Verbal Communication | Communication behaviors | Communication behaviors |
|  | Patient and Clinical Context | Patient-Centered Care | Perceived relevance | Educational relevance |
| (Janda, 2004) [51] | Critical Thinking and Reasoning | Cognitive Processes | Critical thinking | Critical questions |
|  | Emotional and Psychological Outcomes | Personal Growth | Professionalism | Professional behavior |
|  | Engagement and Learning Experience | Learner Engagement | Learning time commitment | Time spent |
| (Ju, 2017) [52] | Educational Process | Effectiveness of Intervention | Effectiveness of learning tools | Usefulness of video monitoring |
|  | Feedback and Assessment | Evaluation | Self-assessment | Students' performance self-evaluation |
|  | Feedback and Assessment | Feedback Methods | Feedback from standardized patients (SP) | Standardized patient evaluation |
| (Kalish, 2011) [53] | Educational Process | Effectiveness of Intervention | Effectiveness of the intervention | Effectiveness of the intervention |
|  | Feedback and Assessment | Evaluation | Self-assessment | Student Self-Assessment |
|  | Feedback and Assessment | Feedback Methods | Feedback effectiveness | Feedback (preceptor, fourth-year students, and patient) |
| (Kalwitzki, 2005) [54] | Clinical Competence | Clinical Skills | Clinical competence | Confidence in dealing with patients |
|  | Communication Skills | Patient Interaction | Patient interaction and care | Handling of patients with fear or pain |
|  | Communication Skills | Verbal and Non-Verbal Communication | Communication skills | Verbal and non-verbal communication |
|  | Engagement and Learning Experience | Learning Environment | Ergonomics | Ergonomics |
| (Kamin, 2003) [55] | Critical Thinking and Reasoning | Cognitive Processes | Critical thinking | Critical-thinking (problem identification, problem description, problem exploration, applicability and integration) |
| (Lee, 2013) [56] | Clinical Competence | Clinical Skills | Interview Skills | Specific students' interview skills (Type of question, timeline, positive verbal reinforcement, therapeutic sequence, facilitative behavior, pace of interview, summary and verification, avoidance of jargon, maintaining a respectful tone, addressing another concern) |
|  | Clinical Competence | Clinical Skills | Interview Skills | Overall students' interview performance |
|  | Educational Process | Effectiveness of Intervention | Effectiveness of the intervention | Effectiveness of the intervention |
| (Leeds, 2020) [57] | Communication Skills | Patient Interaction | Patient-centeredness | Attitudes (empathy, willingness to treat, and perception) |
|  | Critical Thinking and Reasoning | Cognitive Processes | Knowledge acquisition | Knowledge (students' understanding) |
| (Leng, 2007) [58] | Educational Process | Effectiveness of Intervention | Effectiveness of learning tools | Conditions for Productive Use of Video Cases |
|  | Patient and Clinical Context | Patient-Centered Care | Perceived relevance | Perceived Added Value of Video Cases |
| (Leone, 2006) [59] | Clinical Competence | Clinical Skills | Clinical competence | Adherence to guidelines |
|  | Clinical Competence | Clinical Skills | Clinical competence | Clinical competence |
|  | Clinical Competence | Clinical Skills | Error identification and reduction | Error identification and reduction |
|  | Educational Process | Learning Process | Effectiveness of the learning process | Educational reinforcement |
|  | Teamwork and Interprofessional Learning | Team Dynamics | Team function | Team Function and Communication |
| (Lewis, 2015) [60] | Educational Process | Effectiveness of Intervention | Effectiveness of learning tools | Usefulness for learning |
|  | Emotional and Psychological Outcomes | Emotional Impact | Emotional responses | Emotional responses |
|  | Engagement and Learning Experience | Learner Engagement | Learner satisfaction | Overall satisfaction |
|  | Engagement and Learning Experience | Learning Environment | Feasibility | Feasibility |
| (Malon, 2014) [61] | Clinical Competence | Performance | Overall performance | Overall performance |
|  | Clinical Competence | Performance | Reliability | Intraobserver and interobserver reliability |
|  | Clinical Competence | Performance | Skill proficiency | Single domain performance |
| (McQueen, 2019) [62] | Clinical Competence | Clinical Skills | Technical and non-technical skills | Technical skills |
|  | Clinical Competence | Clinical Skills | Technical and non-technical skills | Non-technical skills |
| (Miller, 2015) [63] | Clinical Competence | Performance | Performance assessment | Student performance |
|  | Engagement and Learning Experience | Learner Engagement | Learner perceptions | Perceptions |
| (Minardi, 1999) [64] | Clinical Competence | Performance | Analytical skills | Categorisation of statements |
| (Muench, 2013) [65] | Clinical Competence | Performance | Efficiency | Visit Efficiency |
|  | Clinical Competence | Performance | Overall performance | Overall Medical Care |
|  | Communication Skills | Patient Interaction | Shared decision-making | Shared Decision Making |
|  | Communication Skills | Verbal and Non-Verbal Communication | Communication behaviors | Communication Skills |
|  | Emotional and Psychological Outcomes | Emotional Impact | Emotional responses | Anxiety levels |
|  | Engagement and Learning Experience | Learner Engagement | Learner satisfaction | Residents' Satisfaction |
| (Murphy, 2018) [66] | Clinical Competence | Clinical Skills | Clinical reasoning | Clinical reasoning |
| (Nilsen, 2005) [67] | Emotional and Psychological Outcomes | Emotional Impact | Confidence | Self-esteem and confidence |
|  | Emotional and Psychological Outcomes | Emotional Impact | Emotional responses | Emotional distress and apprehension |
|  | Engagement and Learning Experience | Motivation | Acceptance of the intervention | Overall acceptance of the intervention method |
|  | Engagement and Learning Experience | Motivation | Need for reassurance | Need for reassurance regarding consultation skills |
|  | Feedback and Assessment | Feedback Methods | Feedback effectiveness | Perception of the feedback process (constructiveness, supportiveness) |
| (Nissen, 2024) [68] | Communication Skills | Patient Interaction | Patient-centeredness | Attitudes Toward Person-Centered Care |
|  | Communication Skills | Reflection on Communication | Reflective thinking | Self-Reflection and Critical Thinking |
|  | Engagement and Learning Experience | Learner Engagement | Student engagement | Engagement and Learning Experience |
|  | Teamwork and Interprofessional Learning | Team Dynamics | Interprofessional roles and competencies | Understanding of Interprofessional Roles |
|  | Teamwork and Interprofessional Learning | Team Dynamics | Teamwork and Collaboration | Teamwork and Collaboration |
| (Noverati, 2020) [69] | Critical Thinking and Reasoning | Cognitive Processes | Cognitive load | Cognitive Load |
|  | Critical Thinking and Reasoning | Cognitive Processes | Critical thinking | Critical Thinking and Clinical Reasoning |
|  | Critical Thinking and Reasoning | Cognitive Processes | Knowledge retention | Knowledge Acquisition and Retention |
|  | Engagement and Learning Experience | Learner Engagement | Learner preferences | Student Preferences |
|  | Engagement and Learning Experience | Learning Environment | Authenticity | Authenticity and Memorability |
| (Nunohara, 2020) [70] | Clinical Competence | Clinical Skills | Clinical competence | Clinical procedures |
|  | Clinical Competence | Clinical Skills | Clinical decision-making | Clinical decision-making |
|  | Clinical Competence | Clinical Skills | Clinical focus | Psychosocial vs. Biomedical focus |
|  | Communication Skills | Patient Interaction | Patient-centeredness | Empathy and Patient-Centeredness |
| (Nyström, 2014) [71] | Communication Skills | Verbal and Non-Verbal Communication | Communication skills | Dialogue and Acknowledgement |
|  | Critical Thinking and Reasoning | Reflection | Self-reflection and professional growth | Self-Knowledge and Professional Growth |
|  | Educational Process | Effectiveness of Intervention | Effectiveness of the intervention | Effectiveness of the intervention |
|  | Emotional and Psychological Outcomes | Emotional Impact | Emotional responses | Nervousness and Apprehension |
| (Omar, 2021) [72] | Clinical Competence | Performance | Performance assessment | Faculty evaluation |
|  | Clinical Competence | Performance | Performance assessment | Student performance |
|  | Feedback and Assessment | Evaluation | Self-assessment | Self-evaluation |
|  | Feedback and Assessment | Feedback Methods | Feedback effectiveness | Feedback from SPs (Standardized Patients) |
| (Oosthuizen, 2019) [73] | Clinical Competence | Clinical Skills | Clinical awareness | Awareness of Clinical Populations |
|  | Communication Skills | Reflection on Communication | Self-reflection and skills development | Reflection on Skills Needed |
|  | Critical Thinking and Reasoning | Cognitive Processes | Cognitive load | Cognitive Load |
|  | Engagement and Learning Experience | Learner Engagement | Engagement | Understanding and Engagement |
|  | Engagement and Learning Experience | Motivation | Motivation | Motivation and Relevance |
| (Parlak Özer, 2024) [74] | Communication Skills | Patient Interaction | Patient counseling skills | Patient counseling competence |
|  | Communication Skills | Verbal and Non-Verbal Communication | Communication and interpersonal skills | Interactional competence |
|  | Communication Skills | Verbal and Non-Verbal Communication | Communication skills | Listenership Skills |
|  | Feedback and Assessment | Feedback Methods | Feedback effectiveness | Feedback from patients |
| (Quinn, 2015) [75] | Communication Skills | Reflection on Communication | Self-reflection on communication | Reflection on their communication strategies |
|  | Communication Skills | Verbal and Non-Verbal Communication | Non-verbal communication awareness | Awareness of non-verbal communication |
|  | Educational Process | Effectiveness of Intervention | Perceived intervention effectiveness | Perceived benefits of intervention |
| (Raja, 2008) [76] | Engagement and Learning Experience | Learner Engagement | Learning preferences | Learning styles |
|  | Feedback and Assessment | Evaluation | Assessment of knowledge | Test scores |
|  | Learning Outcomes and Retention | Learning Assessment | Learning outcomes | Learning outcomes |
| (Reher, 2020) [77] | Communication Skills | Verbal and Non-Verbal Communication | Communication skills | Communication skills development |
|  | Critical Thinking and Reasoning | Cognitive Processes | Knowledge acquisition | Knowledge |
|  | Engagement and Learning Experience | Learner Engagement | Engagement | Student engagement |
|  | Engagement and Learning Experience | Learner Engagement | Learner satisfaction | Satisfaction with assessments |
| (Roberts, 2023) [78] | Clinical Competence | Clinical Skills | Clinical documentation | Clinical documentation task performance |
|  | Clinical Competence | Performance | Efficiency | Completion rates and times |
|  | Feedback and Assessment | Feedback Methods | Feedback effectiveness | Student feedback |
| (Rodríguez-Bailón, 2021) [79] | Clinical Competence | Clinical Skills | Clinical reasoning | Clinical reasoning |
|  | Engagement and Learning Experience | Motivation | Motivation | Motivation for learning |
| (Roland, 2012) [15] | Critical Thinking and Reasoning | Cognitive Processes | Knowledge acquisition | Knowledge gain |
|  | Engagement and Learning Experience | Learner Engagement | Learner behavior | Learner behavior |
|  | Engagement and Learning Experience | Learner Engagement | Learner satisfaction | Learner satisfaction |
|  | Engagement and Learning Experience | Learning Environment | Organizational change | Organizational change |
| (Roland, 2015) [80] | Clinical Competence | Clinical Skills | Clinical reasoning | Clinical reasoning |
|  | Clinical Competence | Clinical Skills | Diagnostic accuracy | Diagnostic accuracy |
|  | Clinical Competence | Clinical Skills | Observational skills | Observational skills |
|  | Emotional and Psychological Outcomes | Emotional Impact | Confidence | Confidence in clinical skills |
|  | Teamwork and Interprofessional Learning | Team Dynamics | Collaborative learning | Collaborative learning |
| (Roy, 2012) [81] | Critical Thinking and Reasoning | Cognitive Processes | Critical thinking | Depth of thinking (deep vs superficial) |
|  | Critical Thinking and Reasoning | Cognitive Processes | Critical thinking | Critical thinking |
|  | Engagement and Learning Experience | Learner Engagement | Learner preferences | Student and tutor preferences |
| (Sanders, 2008) [82] | Critical Thinking and Reasoning | Cognitive Processes | Knowledge acquisition | Knowledge |
|  | Emotional and Psychological Outcomes | Emotional Impact | Emotional responses | Comfort levels |
| (Sanderson, 2016) [83] | Feedback and Assessment | Evaluation | Expert assessment | Expert assessment |
|  | Feedback and Assessment | Evaluation | Instructor assessment | Instructor assessment |
|  | Feedback and Assessment | Evaluation | Self-assessment | Student self-assessment |
| (Scheidt, 1986) [84] | Clinical Competence | Clinical Skills | Examination Skills | Examination Skills |
|  | Clinical Competence | Clinical Skills | Interview Skills | Interview Skills |
|  | Clinical Competence | Performance | Overall performance | Overall performance |
| (Schwartz, 2012) [85] | Communication Skills | Patient Interaction | Empathy | Empathy |
|  | Educational Process | Effectiveness of Intervention | Effectiveness of the intervention | Effectiveness of intervention |
|  | Emotional and Psychological Outcomes | Personal Growth | Professionalism | Professionalism |
| (Temple, 2022) [86] | Educational Process | Learning Process | Preparedness | Preparedness for Fieldwork |
|  | Patient and Clinical Context | Patient-Centered Care | Preferences for learning tools | Learning tool preferences |
| (Terasaki, 1984) [87] | Communication Skills | Verbal and Non-Verbal Communication | Communication behaviors | Verbal interactions |
| (Thomas, 2023) [88] | Communication Skills | Verbal and Non-Verbal Communication | Communication skills | Student feedback (communication and patient education skills) |
|  | Feedback and Assessment | Evaluation | Assessment of knowledge | Examination Scores |
| (Tully, 2015) [89] | Communication Skills | Reflection on Communication | Self-reflection | Self-Evaluation of Experience |
|  | Educational Process | Effectiveness of Intervention | Effectiveness of learning tools | Comparison of Video Perspectives |
|  | Educational Process | Learning Process | Follow-up responses | Follow-Up Responses |
|  | Patient and Clinical Context | Patient-Centered Care | Perceived relevance | Perceived Utility of the Intervention |
| (Vessey, 2002) [90] | Clinical Competence | Clinical Skills | Clinical examination skills | Thoroughness of Clinical Examination |
|  | Clinical Competence | Clinical Skills | Clinical reasoning | Correctness of Differential Diagnosis |
|  | Clinical Competence | Performance | Performance assessment | Performance |
| (White, 2008) [91] | Communication Skills | Verbal and Non-Verbal Communication | Communication skills | Communication skills |
| (Yoon, 2016) [92] | Clinical Competence | Clinical Skills | Hypothesis generation | Hypothesis Generation |
|  | Communication Skills | Patient Interaction | Patient-centeredness | Attitude Toward Patients |
|  | Communication Skills | Reflection on Communication | Reflective thinking | Reflective Thinking |
|  | Communication Skills | Verbal and Non-Verbal Communication | Patient-doctor communication | Patient-Doctor Communication |
|  | Critical Thinking and Reasoning | Cognitive Processes | Problem identification | Problem Identification |
|  | Engagement and Learning Experience | Learning Environment | Authenticity | Authenticity |
|  | Engagement and Learning Experience | Motivation | Motivation | Motivation |
|  | Teamwork and Interprofessional Learning | Team Dynamics | Collaborative learning | Collaborative Learning |
| (Zahl, 2016) [93] | Clinical Competence | Clinical Skills | Clinical competence | Clinical Procedures |
|  | Communication Skills | Verbal and Non-Verbal Communication | Communication skills | Interpersonal Communication Skills |
