# Supplementary material for: Video Recording of Patient-Clinician Interactions in Health Education: Scoping Review
Source: JMIR Med Educ. 2026 Jul 13;12:e70324. doi: 10.2196/70324 (PMC13361625; doi:10.2196/70324)
Supplement: Multimedia Appendix 5 [file mededu-v12-e70324-s005.docx]

| **Category** | **Subcategory** | **General Outcome Variable** | **Studies (n)** | **Percentage (%)** |
| --- | --- | --- | --- | --- |
| **Clinical Competence** | Clinical Skills | Clinical competence | 5 | 2.21% |
|  |  | Clinical reasoning | 5 | 2.21% |
|  |  | Interview Skills | 3 | 1.33% |
|  |  | Technical and non-technical skills | 3 | 1.33% |
|  |  | Application of learned skills | 1 | 0.44% |
|  |  | Clinical awareness | 1 | 0.44% |
|  |  | Clinical decision-making | 1 | 0.44% |
|  |  | Clinical documentation | 1 | 0.44% |
|  |  | Clinical examination skills | 1 | 0.44% |
|  |  | Clinical experience sharing | 1 | 0.44% |
|  |  | Clinical focus | 1 | 0.44% |
|  |  | Diagnostic accuracy | 1 | 0.44% |
|  |  | Error identification and reduction | 1 | 0.44% |
|  |  | Examination Skills | 1 | 0.44% |
|  |  | Hypothesis generation | 1 | 0.44% |
|  |  | Observational skills | 1 | 0.44% |
|  |  | Practical application of knowledge | 1 | 0.44% |
|  | Performance | Performance assessment | 7 | 3.10% |
|  |  | Overall performance | 4 | 1.77% |
|  |  | Efficiency | 2 | 0.88% |
|  |  | Analytical skills | 1 | 0.44% |
|  |  | Confidence and competence | 1 | 0.44% |
|  |  | Reliability | 1 | 0.44% |
|  |  | Skill proficiency | 1 | 0.44% |
| **Communication Skills** | Verbal and Non-Verbal Communication | Communication skills | 12 | 5.31% |
|  |  | Communication behaviors | 4 | 1.77% |
|  |  | Communication and interpersonal skills | 1 | 0.44% |
|  |  | Communication challenges | 1 | 0.44% |
|  |  | Communication strategies | 1 | 0.44% |
|  |  | Non-verbal communication awareness | 1 | 0.44% |
|  |  | Patient-doctor communication | 1 | 0.44% |
|  |  | Perceived benefits of communication skills | 1 | 0.44% |
|  |  | Perceived relevance of communication skills | 1 | 0.44% |
|  | Patient Interaction | Patient-centeredness | 5 | 2.21% |
|  |  | Patient interaction and care | 2 | 0.88% |
|  |  | Awareness of clinical populations | 1 | 0.44% |
|  |  | Empathy | 1 | 0.44% |
|  |  | Patient care learning | 1 | 0.44% |
|  |  | Patient counseling skills | 1 | 0.44% |
|  |  | Shared decision-making | 1 | 0.44% |
|  | Reflection on Communication | Self-reflection | 5 | 2.21% |
|  |  | Reflective thinking | 2 | 0.88% |
|  |  | Self-reflection and skills development | 1 | 0.44% |
|  |  | Self-reflection on communication | 1 | 0.44% |
| **Engagement and Learning Experience** | Learner Engagement | Learner satisfaction | 6 | 2.65% |
|  |  | Engagement | 5 | 2.21% |
|  |  | Student engagement | 3 | 1.33% |
|  |  | Learner perceptions | 2 | 0.88% |
|  |  | Learner preferences | 2 | 0.88% |
|  |  | Learning and satisfaction | 1 | 0.44% |
|  |  | Learning preferences | 1 | 0.44% |
|  |  | Learning time commitment | 1 | 0.44% |
|  |  | Learner behavior | 1 | 0.44% |
|  | Learning Environment | Feasibility | 3 | 1.33% |
|  |  | Authenticity | 2 | 0.88% |
|  |  | Authenticity and realism | 1 | 0.44% |
|  |  | Emotional intelligence | 1 | 0.44% |
|  |  | Ergonomics | 1 | 0.44% |
|  |  | Organizational change | 1 | 0.44% |
|  | Learning Assessment | Learning outcomes | 3 | 1.33% |
|  |  | Hands-on experience | 1 | 0.44% |
|  |  | Learning reinforcement | 1 | 0.44% |
|  |  | Log Activity Data | 1 | 0.44% |
|  | Motivation | Motivation | 3 | 1.33% |
|  |  | Barriers to learning | 1 | 0.44% |
|  |  | Intent to change behavior | 1 | 0.44% |
|  |  | Need for reassurance | 1 | 0.44% |
|  | Educational Tools | Perception of video realism | 1 | 0.44% |
| **Critical Thinking and Reasoning** | Cognitive Processes | Critical thinking | 7 | 3.10% |
|  |  | Knowledge acquisition | 6 | 2.65% |
|  |  | Knowledge retention | 2 | 0.88% |
|  |  | Long-term retention | 1 | 0.44% |
|  |  | Problem identification | 1 | 0.44% |
|  | Reflection | Self-reflection and improvement | 2 | 0.88% |
|  |  | Cognitive load | 2 | 0.88% |
|  |  | Self-awareness | 1 | 0.44% |
|  |  | Self-reflection and professional growth | 1 | 0.44% |
| **Feedback and Evaluation** | Feedback Methods | Feedback effectiveness | 11 | 4.87% |
|  |  | Feedback from standardized patients (SP) | 1 | 0.44% |
|  | Evaluation | Self-assessment | 6 | 2.65% |
|  |  | Assessment of knowledge | 2 | 0.88% |
|  |  | Assessment of learning outcomes | 1 | 0.44% |
|  |  | Expert assessment | 1 | 0.44% |
|  |  | Instructor assessment | 1 | 0.44% |
| **Emotional and Psychological Outcomes** | Emotional Impact | Emotional responses | 8 | 3.54% |
|  |  | Confidence | 2 | 0.88% |
|  |  | Affective learning outcomes | 1 | 0.44% |
|  |  | Affective outcomes | 1 | 0.44% |
|  | Personal Growth | Professionalism | 2 | 0.88% |
|  |  | Professional identity | 1 | 0.44% |
|  |  | Self-directed learning | 1 | 0.44% |
|  |  | Self-reported measures | 1 | 0.44% |
| **Educational Process** | Effectiveness of Intervention | Effectiveness of learning tools | 4 | 1.77% |
|  |  | Effectiveness of the intervention | 4 | 1.77% |
|  |  | Perceived intervention effectiveness | 1 | 0.44% |
|  | Learning Process | Effectiveness of the learning process | 2 | 0.88% |
|  |  | Effectiveness of the interactive materials | 1 | 0.44% |
|  |  | Follow-up responses | 1 | 0.44% |
|  |  | Preparedness | 1 | 0.44% |
| **Teamwork and Interprofessional Learning** | Team Dynamics | Collaborative learning | 2 | 0.88% |
|  |  | Interprofessional roles and competencies | 2 | 0.88% |
|  |  | Teamwork and Collaboration | 2 | 0.88% |
|  |  | Behavioral Analysis | 1 | 0.44% |
|  |  | Team function | 1 | 0.44% |
| **Patient and Clinical Context** | Patient-Centered Care | Perceived relevance | 5 | 2.21% |
|  |  | Perceived benefits of role understanding | 1 | 0.44% |
|  |  | Preferences for learning tools | 1 | 0.44% |
