# Supplementary material for: Video Recording of Patient-Clinician Interactions in Health Education: Scoping Review
Source: JMIR Med Educ. 2026 Jul 13;12:e70324. doi: 10.2196/70324 (PMC13361625; doi:10.2196/70324)
Supplement: Multimedia Appendix 6 [file mededu-v12-e70324-s006.docx]

| **(Author, Year)** | **Category** | **Subcategory** | **General Outcome Variable** |
| --- | --- | --- | --- |
| (Ahmet, 2018) [28] | Technical & Performance Assessments | Objective & Structured Assessments | Global Assessment Scale |
|  | Technical & Performance Assessments | Objective & Structured Assessments | Objective Structured Assessment of Technical Skills (OSATS) |
|  | Peer & Self-Assessment | Self-Assessments | Self-Evaluation Test |
|  | Observations & Analysis Tools | Observation Tools | Observer’s Checklist |
|  | Peer & Self-Assessment | Self-Assessments | Quiz results |
|  | Peer & Self-Assessment | Self-Assessments | Multiple Choice Test |
|  | Peer & Self-Assessment | Self-Assessments | Retention Tests |
|  | Peer & Self-Assessment | Self-Assessments | Expert-Based Assessments |
| (Alsalamah, 2023) [29] | Interviews & Focus Groups | Semi-structured Interviews | Semi-structured interviews |
| (Balslev, 2005) [30] | Interviews & Focus Groups | Recorded & Transcribed Data | Recorded group discussions transcribed verbatim for analysis |
| (Batteson, 2023) [31] | Questionnaires & Surveys | Specific Topic-Based Questionnaires | IPEC Competency Complexity Questionnaire |
|  | Questionnaires & Surveys | Specific Topic-Based Questionnaires | Interprofessional Collaborative Competencies Attainment Survey (ICCAS) |
|  | Questionnaires & Surveys | Specific Topic-Based Questionnaires | Froehlich Communication Survey |
|  | Questionnaires & Surveys | Specific Topic-Based Questionnaires | Care Plan Evaluation |
| (Botelho, 2016) [34] | Questionnaires & Surveys | General Surveys | Questionnaire |
|  | Observations & Analysis Tools | Transcription & Coding | Transcripts of tutorials |
|  | Data & Analytics | Activity Logs & Analytics | Usage analytics |
| (Bowles, 2020) [35] | Questionnaires & Surveys | General Surveys | Survey |
|  | Observations & Analysis Tools | Transcription & Coding | Transcription of discussions |
| (Chan, 2010) [36] | Questionnaires & Surveys | General Surveys | Questionnaire |
| (Chi, 2014) [37] | Questionnaires & Surveys | General Surveys | Questionnaire |
| (Courteille, 2014) [38] | Questionnaires & Surveys | General Surveys | Questionnaire |
|  | Observations & Analysis Tools | Observation Tools | Video Observational Data |
|  | Data & Analytics | Activity Logs & Analytics | Log Activity Data |
|  | Interviews & Focus Groups | Semi-structured Interviews | Semi-structured interviews |
| (Davies, 2017) [39] | Observations & Analysis Tools | Conversation & Thematic Analysis | Conversation analysis |
| (Edrees, 2014) [40] | Questionnaires & Surveys | General Surveys | Questionnaire |
| (Farnan, 2013) [41] | Questionnaires & Surveys | General Surveys | Survey |
| (Flood, 2019) [43] | Questionnaires & Surveys | General Surveys | Survey |
| (Forbes, 2016) [44] | Questionnaires & Surveys | General Surveys | Survey |
| (Hafen, 2015) [47] | Questionnaires & Surveys | General Surveys | Survey |
|  | Questionnaires & Surveys | General Surveys | Survey |
| (Hammarström, 2021) [48] | Interviews & Focus Groups | Semi-structured Interviews | Semi-structured interviews |
| (Hammoud, 2012) [49] | Peer & Self-Assessment | Self-Assessments | Self-Assessment questionnaires |
|  | Peer & Self-Assessment | Peer & Faculty Assessments | Faculty assessment |
|  | Peer & Self-Assessment | Peer & Faculty Assessments | Peer assessment |
|  | Questionnaires & Surveys | General Surveys | Satisfaction surveys |
|  | Technical & Performance Assessments | Performance Metrics | Performance metrics |
| (Henry, 2020) [20] | Questionnaires & Surveys | General Surveys | Questionnaire |
|  | Observations & Analysis Tools | Transcription & Coding | Transcription protocols |
|  | Interviews & Focus Groups | Video Elicitation Interviews | Video elicitation interviews |
|  | Observations & Analysis Tools | Conversation & Thematic Analysis | Conversation analysis |
|  | Observations & Analysis Tools | Microanalysis & Critical Thinking | Microanalysis of dialogue |
| (Henry, 2012) [50] | Interviews & Focus Groups | Semi-structured Interviews | Interview protocols |
| (Ju, 2017) [52] | Questionnaires & Surveys | General Surveys | Questionnaire |
|  | Peer & Self-Assessment | Self-Assessments | Self-Assessment Scale |
|  | Specialized Instruments & Scales | Specialized Questionnaires & Scales | Standardized Patient Evaluation Scores |
|  | Questionnaires & Surveys | Specific Topic-Based Questionnaires | Qualitative open-ended feedback |
| (Kalish, 2011) [53] | Interviews & Focus Groups | Focus Group Discussions | Focus Group Discussions |
|  | Questionnaires & Surveys | Specific Topic-Based Questionnaires | Compassionate Care Interactions Questionnaire |
|  | Questionnaires & Surveys | Specific Topic-Based Questionnaires | Patient-Partner Questionnaire |
| (Kalwitzki, 2005) [54] | Questionnaires & Surveys | General Surveys | Questionnaire |
| (Kamin, 2003) [55] | Observations & Analysis Tools | Transcription & Coding | Transcription of discussions |
|  | Observations & Analysis Tools | Transcription & Coding | Content analysis coding |
|  | Observations & Analysis Tools | Microanalysis & Critical Thinking | Critical-thinking ratios |
| (Lee, 2013) [56] | Grading & Rubrics | Grading Tools | Interview skill checklist modified from ACIRS |
| (Leeds, 2020) [57] | Questionnaires & Surveys | General Surveys | Questionnaire |
|  | Questionnaires & Surveys | General Surveys | Survey |
| (Leng, 2007) [58] | Interviews & Focus Groups | Focus Group Discussions | Focus Group Discussions |
|  | Observations & Analysis Tools | Conversation & Thematic Analysis | Thematic Analysis |
| (Leone, 2006) [59] | Feedback & Satisfaction Tools | Feedback Mechanisms | Feedback mechanisms |
| (Lewis, 2015) [60] | Questionnaires & Surveys | General Surveys | Questionnaire |
|  | Observations & Analysis Tools | Conversation & Thematic Analysis | Thematic Analysis |
| (Malon, 2014) [61] | Technical & Performance Assessments | Objective & Structured Assessments | Structured assessment tool |
|  | Technical & Performance Assessments | Performance Metrics | Interclass correlation coefficients |
| (McQueen, 2019) [62] | Technical & Performance Assessments | Objective & Structured Assessments | Objective Structured Assessment of Technical Skills (OSATS) |
|  | Technical & Performance Assessments | Objective & Structured Assessments | Global Rating Index for Technical Skills (GRITS) |
|  | Technical & Performance Assessments | Objective & Structured Assessments | Global Operative Assessment of Laparoscopic Skills (GOALS) |
|  | Technical & Performance Assessments | Objective & Structured Assessments | Bariatric Objective Structured Assessment of Technical Skill (BOSATS) |
|  | Technical & Performance Assessments | Objective & Structured Assessments | Non-Technical Skills for Surgeons (NOTSS) |
| (Miller, 2015) [63] | Questionnaires & Surveys | General Surveys | Questionnaire |
| (Muench, 2013) [65] | Questionnaires & Surveys | General Surveys | Survey |
|  | Grading & Rubrics | Grading Tools | Checklist based on Medical Interview Skills Competency Evaluation (MISCE) |
|  | Feedback & Satisfaction Tools | Feedback Mechanisms | Feedback forms |
|  | Questionnaires & Surveys | General Surveys | Surveys |
| (Nilsen, 2005) [67] | Interviews & Focus Groups | Focus Group Discussions | Focus Group Discussions |
|  | Observations & Analysis Tools | Conversation & Thematic Analysis | Phenomenological Qualitative Analysis |
| (Nissen, 2024) [68] | Interviews & Focus Groups | Focus Group Discussions | Focus Group Discussions |
|  | Interviews & Focus Groups | Semi-structured Interviews | Interview Guide |
|  | Observations & Analysis Tools | Conversation & Thematic Analysis | Thematic Analysis |
| (Omar, 2021) [72] | Questionnaires & Surveys | General Surveys | Questionnaire |
| (Oosthuizen, 2019) [73] | Interviews & Focus Groups | Focus Group Discussions | Focus Group Discussions |
| (Parlak Özer, 2024) [74] | Observations & Analysis Tools | Conversation & Thematic Analysis | Conversation analysis |
| (Quinn, 2015) [75] | Observations & Analysis Tools | Conversation & Thematic Analysis | Thematic Analysis |
| (Raja, 2008) [76] | Questionnaires & Surveys | General Surveys | Survey |
| (Reher, 2020) [77] | Questionnaires & Surveys | General Surveys | Survey |
| (Roberts, 2023) [78] | Feedback & Satisfaction Tools | Feedback Mechanisms | Student feedback surveys |
|  | Grading & Rubrics | Grading Tools | Grading rubric |
|  | Technical & Performance Assessments | Performance Metrics | Clinical documentation tasks |
| (Rodriguez-Bailón, 2021) [79] | Questionnaires & Surveys | General Surveys | Survey |
|  | Questionnaires & Surveys | General Surveys | Survey |
| (Roland, 2015) [80] | Feedback & Satisfaction Tools | Feedback Mechanisms | Feedback mechanisms |
|  | Observations & Analysis Tools | Observation Tools | Facilitator Observations |
|  | Interviews & Focus Groups | Focus Group Discussions | Group Discussions |
|  | Technical & Performance Assessments | Performance Metrics | Diagnostic Accuracy Exercises |
|  | Feedback & Satisfaction Tools | Peer-led Approaches | Peer-led Approaches |
| (Roy, 2012) [81] | Observations & Analysis Tools | Microanalysis & Critical Thinking | Critical-thinking ratios |
| (Schwartz, 2012) [85] | Questionnaires & Surveys | General Surveys | Questionnaire |
| (Temple, 2022) [86] | Questionnaires & Surveys | General Surveys | Survey |
| (Terasaki, 1984) [87] | Specialized Instruments & Scales | Specialized Questionnaires & Scales | Reciprocal Category Analysis (RCA) |
| (White, 2008) [91] | Questionnaires & Surveys | General Surveys | Questionnaire |
