# Supplementary material for: Video Recording of Patient-Clinician Interactions in Health Education: Scoping Review
Source: JMIR Med Educ. 2026 Jul 13;12:e70324. doi: 10.2196/70324 (PMC13361625; doi:10.2196/70324)
Supplement: Multimedia Appendix 7 [file mededu-v12-e70324-s007.docx]

| **(Author, Year)** | **Informed Consent** | **Confidentiality and Privacy** | **Emotional Impact** | **Ethical Use of Recordings** | **Regulatory Compliance** | **Additional Ethical Challenges** |
| --- | --- | --- | --- | --- | --- | --- |
| (Alsalamah, 2023) [29] | ✓ | ✓ | ✓ | ✓ |  | Addressing participant discomfort, feedback mechanisms |
| (Batteson, 2023) [31] | ✓ | ✓ |  | ✓ |  | Balancing patient comfort with educational value |
| (Bessette, 2021) [32] | ✓ | ✓ |  | ✓ |  | Anonymizing data |
| (Botelho, 2016) [34] | ✓ | ✓ |  |  |  | Avoiding identifiable data, balancing ethical concerns |
| (Chan, 2010) [36] | ✓ | ✓ | ✓ |  |  |  |
| (Courteille, 2014) [38] | ✓ | ✓ |  | ✓ |  |  |
| (Farnan, 2013) [41] | ✓ | ✓ |  | ✓ |  | Ethical training for clinicians and students |
| (Forbes, 2016) [44] | ✓ | ✓ |  | ✓ |  |  |
| (Giles, 2014) [45] | ✓ | ✓ |  |  |  | Ethical feedback practices |
| (Hammoud, 2012) [49] | ✓ | ✓ |  |  | ✓ | Balancing recording benefits with patient impact |
| (Hammarström, 2021) [48] | ✓ | ✓ | ✓ |  |  | Participant comfort, addressing anxiety |
| (Henry, 2020) [20] | ✓ | ✓ |  |  | ✓ | Challenges with IRB approvals |
| (Ju, 2017) [52] | ✓ | ✓ |  |  |  | Anonymization of recordings |
| (Kalish, 2011) [53] | ✓ | ✓ |  | ✓ |  |  |
| (Leeds, 2020) [57] | ✓ | ✓ | ✓ |  |  | Bias in representation |
| (Leone, 2006) [59] | ✓ | ✓ |  |  |  | Ethical concerns during emergencies |
| (Malon, 2014) [61] | ✓ | ✓ |  |  | ✓ | Balancing ethical concerns with educational outcomes |
| (McQueen, 2019) [62] | ✓ | ✓ |  |  |  | Legal and ethical implications |
| (Minardi, 1999) [64] | ✓ | ✓ |  |  |  | Addressing emotional concerns |
| (Muench, 2013) [65] | ✓ | ✓ | ✓ |  |  |  |
| (Murphy, 2018) [66] | ✓ | ✓ |  |  |  | Ethical justification of recording use |
| (Nunohara, 2020) [70] | ✓ | ✓ |  |  | ✓ | Patient and clinician impact |
| (Nissen, 2024) [68] | ✓ | ✓ | ✓ | ✓ |  |  |
| (Nyström, 2014) [71] | ✓ | ✓ | ✓ |  |  | Balancing benefits with ethical concerns |
| (Parlak Özer, 2024) [74] | ✓ | ✓ |  |  | ✓ | Minimizing impact on participants |
| (Raja, 2008) [76] | ✓ | ✓ |  |  |  | Impact on patient comfort |
| (Roberts, 2023) [78] | ✓ | ✓ |  |  |  | Balancing educational value with patient care quality |
| (Rodríguez-Bailón, 2021) [79] | ✓ | ✓ |  |  |  | Ethical training for participants |
| (Roland, 2015) [80] | ✓ | ✓ |  | ✓ |  |  |
| (Roy, 2012) [81] | ✓ | ✓ |  |  | ✓ | Balancing cognitive load |
| (Terasaki, 1984) [87] | ✓ | ✓ | ✓ |  |  | Ethical use of simulated scenarios |
| (Tully, 2015) [89] | ✓ | ✓ |  |  |  | Addressing potential distraction by new technologies |
| (Vessey, 2002) [90] | ✓ | ✓ |  |  |  | Ethical treatment of simulated patients |

**Table Key**: ✓ = Ethical consideration addressed in the study
