# Supplementary material for: Video Recording of Patient-Clinician Interactions in Health Education: Scoping Review
Source: JMIR Med Educ. 2026 Jul 13;12:e70324. doi: 10.2196/70324 (PMC13361625; doi:10.2196/70324)
Supplement: Multimedia Appendix 8 [file mededu-v12-e70324-s008.docx]

| **(Author, Year)** | **Technical Setup** | **Training for Participants** | **Scheduling Coordination** | **Resource Allocation** | **Feedback Mechanisms** |
| --- | --- | --- | --- | --- | --- |
| (Alsalamah, 2023) [29] | ✓ | ✓ | ✓ | ✓ | ✓ |
| (Batteson, 2023) [31] | ✓ | ✓ | ✓ |  |  |
| (Bessette, 2021) [32] | ✓ |  | ✓ | ✓ |  |
| (Botelho, 2016) [34] | ✓ |  |  | ✓ |  |
| (Chan, 2010) [36] | ✓ |  | ✓ |  |  |
| (Courteille, 2014) [38] | ✓ |  | ✓ |  |  |
| (Farnan, 2013) [41] | ✓ |  | ✓ | ✓ |  |
| (Forbes, 2016) [44] | ✓ |  | ✓ |  | ✓ |
| (Giles, 2014) [45] | ✓ | ✓ | ✓ |  |  |
| (Hammoud, 2012) [49] | ✓ | ✓ | ✓ |  |  |
| (Hammarström, 2021) [48] | ✓ |  | ✓ |  | ✓ |
| (Henry, 2020) [20] | ✓ |  | ✓ |  |  |
| (Ju, 2017) [52] | ✓ | ✓ | ✓ |  |  |
| (Kalish, 2011) [53] | ✓ | ✓ | ✓ |  |  |
| (Leeds, 2020) [57] | ✓ |  | ✓ | ✓ |  |
| (Leone, 2006) [59] | ✓ |  | ✓ |  |  |
| (Malon, 2014) [61] | ✓ |  | ✓ |  |  |
| (McQueen, 2019) [62] | ✓ |  | ✓ | ✓ |  |
| (Minardi, 1999) [64] | ✓ |  | ✓ |  | ✓ |
| (Muench, 2013) [65] | ✓ |  | ✓ | ✓ |  |
| (Murphy, 2018) [66] | ✓ |  |  | ✓ |  |
| (Nunohara, 2020) [70] | ✓ |  | ✓ |  | ✓ |
| (Nissen, 2024) [68] | ✓ |  | ✓ |  |  |
| (Nyström, 2014) [71] | ✓ |  | ✓ |  |  |
| (Parlak Özer, 2024) [74] | ✓ |  | ✓ | ✓ |  |
| (Raja, 2008) [76] | ✓ |  | ✓ | ✓ |  |
| (Roberts, 2023) [78] | ✓ |  | ✓ | ✓ |  |
| (Rodríguez-Bailón, 2021) [79] | ✓ |  | ✓ |  | ✓ |
| (Roland, 2015) [80] | ✓ | ✓ | ✓ |  |  |
| (Roy, 2012) [81] | ✓ |  | ✓ | ✓ |  |
| (Terasaki, 1984) [87] | ✓ |  | ✓ | ✓ |  |
| (Tully, 2015) [89] | ✓ | ✓ |  | ✓ |  |
| (Vessey, 2002) [90] | ✓ |  | ✓ |  |  |

**Table Key**: ✓ = Logistical consideration addressed in the study
