# Supplementary material for: Video Recording of Patient-Clinician Interactions in Health Education: Scoping Review
Source: JMIR Med Educ. 2026 Jul 13;12:e70324. doi: 10.2196/70324 (PMC13361625; doi:10.2196/70324)
Supplement: Checklist 2 [file mededu-v12-e70324-s010.doc]

Grey Matters


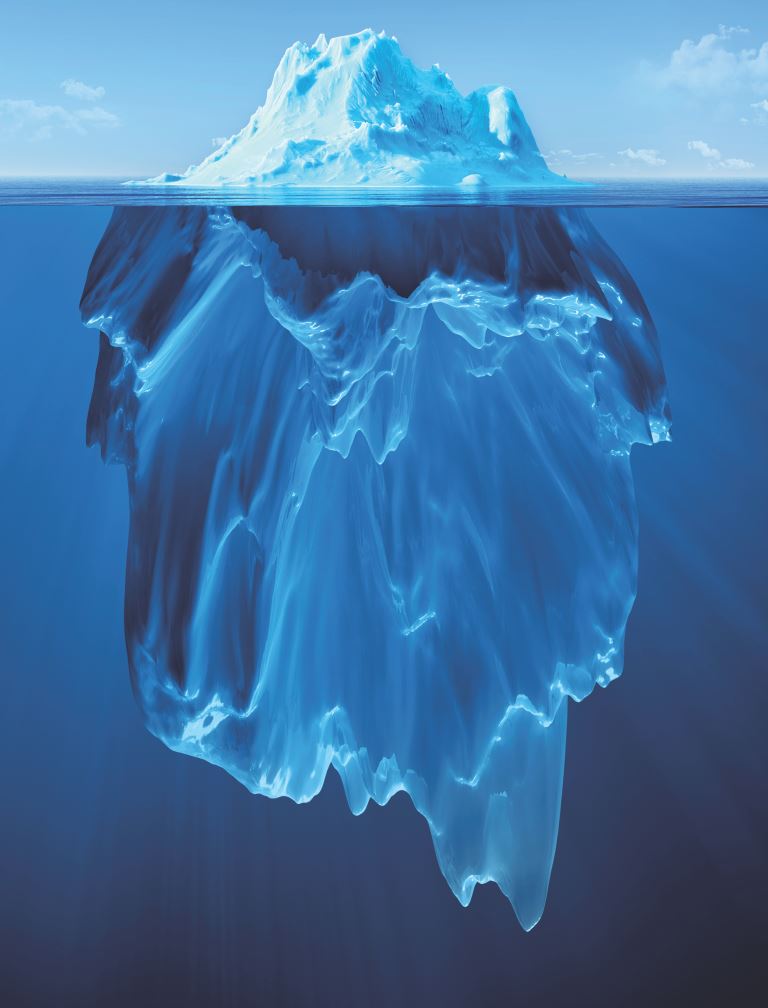


**CADTH Research Information Services**

**Updated: April 2019**

*Grey Matters is an open access tool and may be copied and used for non-commercial purposes, provided that attribution is given to CADTH*

**Cite as:** Grey matters: a practical tool for searching health-related grey literature [Internet]. Ottawa: CADTH; 2018. [cited yyyy mmm dd]. Available from: <https://www.cadth.ca/resources/finding-evidence>.

**Disclaimer:** The information in this document is intended to help Canadian health care decision-makers, health care professionals, health systems leaders, and policy-makers make well-informed decisions and thereby improve the quality of health care services. While patients and others may access this document, the document is made available for informational purposes only and no representations or warranties are made with respect to its fitness for any particular purpose. The information in this document should not be used as a substitute for professional medical advice or as a substitute for the application of clinical judgment in respect of the care of a particular patient or other professional judgment in any decision-making process. The Canadian Agency for Drugs and Technologies in Health (CADTH) does not endorse any information, drugs, therapies, treatments, products, processes, or services.

While care has been taken to ensure that the information prepared by CADTH in this document is accurate, complete, and up-to-date as at the applicable date the material was first published by CADTH, CADTH does not make any guarantees to that effect. CADTH does not guarantee and is not responsible for the quality, currency, propriety, accuracy, or reasonableness of any statements, information, or conclusions contained in any third-party materials used in preparing this document. The views and opinions of third parties published in this document do not necessarily state or reflect those of CADTH.

CADTH is not responsible for any errors, omissions, injury, loss, or damage arising from or relating to the use (or misuse) of any information, statements, or conclusions contained in or implied by the contents of this document or any of the source materials.

This document may contain links to third-party websites. CADTH does not have control over the content of such sites. Use of third-party sites is governed by the third-party website owners’ own terms and conditions set out for such sites. CADTH does not make any guarantee with respect to any information contained on such third-party sites and CADTH is not responsible for any injury, loss, or damage suffered as a result of using such third-party sites. CADTH has no responsibility for the collection, use, and disclosure of personal information by third-party sites.

Subject to the aforementioned limitations, the views expressed herein are those of CADTH and do not necessarily represent the views of Canada’s federal, provincial, or territorial governments or any third party supplier of information.

This document is prepared and intended for use in the context of the Canadian health care system. The use of this document outside of Canada is done so at the user’s own risk.

This disclaimer and any questions or matters of any nature arising from or relating to the content or use (or misuse) of this document will be governed by and interpreted in accordance with the laws of the Province of Ontario and the laws of Canada applicable therein, and all proceedings shall be subject to the exclusive jurisdiction of the courts of the Province of Ontario, Canada.

The copyright and other intellectual property rights in this document are owned by CADTH and its licensors. These rights are protected by the Canadian *Copyright Act* and other national and international laws and agreements. Users are permitted to make copies of this document for non-commercial purposes only, provided it is not modified when reproduced and appropriate credit is given to CADTH and its licensors.

**About CADTH:** CADTH is an independent, not-for-profit organization responsible for providing Canada’s health care decision-makers with objective evidence to help make informed decisions about the optimal use of drugs, medical devices, diagnostics, and procedures in our health care system.

**Funding:** CADTH receives funding from Canada’s federal, provincial, and territorial governments, with the exception of Quebec.

**Table of Contents**

[Introduction 1](#__RefHeading___Toc520972079)

[Background 1](#__RefHeading___Toc520972080)

[Audience 1](#__RefHeading___Toc520972081)

[Scope 1](#__RefHeading___Toc520972082)

[How To Use This Checklist 1](#__RefHeading___Toc520972083)

[CADTH Cadth Searching Tools And Resources 2](#__RefHeading___Toc520972084)

[Grey Literature Checklist 3](#__RefHeading___Toc520972085)

[Health Technology Assessment (HTA) Agencies 3](#__RefHeading___Toc520972086)

[Canada 3](#__RefHeading___Toc520972087)

[International 6](#__RefHeading___Toc520972088)

[Australia 7](#__RefHeading___Toc520972089)

[Austria 8](#__RefHeading___Toc520972090)

[Belgium 8](#__RefHeading___Toc520972091)

[Denmark 9](#__RefHeading___Toc520972092)

[France 9](#__RefHeading___Toc520972093)

[Germany 9](#__RefHeading___Toc520972094)

[Ireland 10](#__RefHeading___Toc520972095)

[The Netherlands 10](#__RefHeading___Toc520972096)

[Norway 10](#__RefHeading___Toc520972097)

[Spain 11](#__RefHeading___Toc520972098)

[Sweden 11](#__RefHeading___Toc520972099)

[UK 12](#__RefHeading___Toc520972100)

[US 14](#__RefHeading___Toc520972101)

[Health Economics 15](#__RefHeading___Toc520972102)

[Canada 15](#__RefHeading___Toc520972103)

[International 16](#__RefHeading___Toc520972104)

[Clinical Practice Guidelines 18](#__RefHeading___Toc520972105)

[Canada 18](#__RefHeading___Toc520972106)

[International 20](#__RefHeading___Toc520972107)

[Drug And Device Regulatory Approvals 23](#__RefHeading___Toc520972108)

[Canada 23](#__RefHeading___Toc520972109)

[International 24](#__RefHeading___Toc520972110)

[Advisories And Warnings 26](#__RefHeading___Toc520972111)

[Canada 26](#__RefHeading___Toc520972112)

[International 27](#__RefHeading___Toc520972113)

[Drug Class Reviews 30](#__RefHeading___Toc520972114)

[Clinical Trials Registries 30](#__RefHeading___Toc520972115)

[Canadian Drug Formularies 32](#__RefHeading___Toc520972116)

[Canadian Physician Fees Schedules 32](#__RefHeading___Toc520972117)

[Databases (Free) 34](#__RefHeading___Toc520972118)

[Databases (Subscription-Based) 36](#__RefHeading___Toc520972119)

[Health Statistics 37](#__RefHeading___Toc520972120)

[Canada 37](#__RefHeading___Toc520972121)

[US 39](#__RefHeading___Toc520972122)

[International 39](#__RefHeading___Toc520972123)

[Internet Search 40](#__RefHeading___Toc520972124)

[Search Engines 40](#__RefHeading___Toc520972125)

[Open Access Journals 41](#__RefHeading___Toc520972126)

[Miscellaneous 41](#__RefHeading___Toc520972127)

[Behavioural Change 41](#__RefHeading___Toc520972128)

[Natural Medicine And Environmental Health 41](#__RefHeading___Toc520972129)

[Dentistry 42](#__RefHeading___Toc520972131)

[Diagnostic Tests 42](#__RefHeading___Toc520972132)

[Mental Health 43](#__RefHeading___Toc520972133)

[Nursing 43](#__RefHeading___Toc520972134)

[Physiotherapy/Rehabilitation 43](#__RefHeading___Toc520972135)

# Introduction

## Background

CADTH — the Canadian Agency for Drugs and Technologies in Health — provides Canada’s federal, provincial, and territorial health care decision-makers with objective evidence to help make informed decisions about the optimal use of drugs and medical devices in our health care system. To achieve its mandate, CADTH produces a variety of publications that range from comprehensive systematic reviews and economic evaluations to more concise bulletins and updates.

In order to search for and retrieve the evidence base required to produce CADTH reports, CADTH’s Research Information Services team has developed and made available a grey literature checklist. Grey literature includes government information and reports that are not published commercially and that may be inaccessible via bibliographic databases. This checklist is used to:

- ensure the retrieval of all relevant health technology assessment (HTA), government, and evidence-based agency reports that may not be indexed in bibliographic databases such as MEDLINE
- document the grey literature search process, thereby increasing transparency and the potential for reproducibility
- ensure that grey literature searching is done in a comprehensive way, according to international standards.^1-2^

## Audience

This checklist is intended for librarians; information specialists; and researchers who are producing systematic reviews, HTAs, drug assessments, or economic evaluations.

## Scope

The CADTH grey literature checklist is not exhaustive, but it does strive to be comprehensive regarding Canadian and international HTA agencies. To compensate for the focused characteristics of the checklist, supplemental keyword searches on search engines such as Google are recommended.

The checklist is updated every two years to ensure the website links and search tips are accurate, and to incorporate new sources. However, because of the continuously evolving nature of the Internet, CADTH cannot be held responsible for occasional inaccuracies due to website changes.

## How To Use This Checklist

Organized by topic, the checklist includes tips on navigating the websites. Although the websites are usually assigned to only one category, in many cases an individual website may have relevancy in additional categories. The checklist does not supply background information on the agencies. If available, however, the name of the website is hyperlinked to a brief description of the agency.

This checklist is meant to be used to document all aspects of the search process. This includes the documentation of keywords used in the search, and information about the availability of each website, as well as the use of a drop-down box beside each website to indicate the success or relevancy of the retrieval.

Selective or targeted searching by category may be required, and time spent will vary according to the needs of a particular project. A recent study highlights the average time spent searching grey literature for systematic reviews as being roughly a quarter of overall searching time.^3^  It is not mandatory to search every website on this checklist in order to conduct a comprehensive grey literature search.

## CADTH Searching Tools and Resources

A concise shortlist of the *Grey Matters* checklist has also been produced, entitled *Grey Matters Lite*. *Grey Matters*, *Grey Matters Lite*, and a variety of other literature searching tools and resources provided by CADTH Research Information Services are available at [Finding the Evidence: Literature Searching Tools in Support of Systematic Reviews](https://www.cadth.ca/resources/finding-evidence).

**References**

1. Higgins JPT, Green S, eds.  [Cochrane handbook for systematic reviews of interventions](http://handbook.cochrane.org/). Version 5.1.0. [Updated March 2011].  The Cochrane Collaboration; 2011.
2. Higgins JPT, Lasserson T, Chandler J, Tovey D, Churchill R. [Standards for the conduct of new Cochrane Intervention Reviews](http://community.cochrane.org/mecir-manual/standards-conduct-new-cochrane-intervention-reviews-c1-75). In: Higgins JPT, Lasserson T, Chandler J, Tovey D, Churchill R[. Methodological Expectations of Cochrane Intervention Reviews](http://community.cochrane.org/mecir-manual). Cochrane: London, 2016. See [Searching for studies](http://community.cochrane.org/mecir-manual/standards-conduct-new-cochrane-intervention-reviews-c1-c75/performing-review-c24-75/searching-studies-c24-38)
3. Saleh A, Ratajeski M, Bertolet M. [Grey literature searching for health sciences systematic reviews: a prospective study of time spent and resources utilized](https://journals.library.ualberta.ca/eblip/index.php/EBLIP/article/view/20629/17128). Evidence Based Library and Information Practice 2014;9(3):28-50.

# Grey Literature Checklist

Use the drop-down boxes that appear beside each web site in the checklist to indicate

one of the following:

1. **searched; nothing found**
2. **not searched; not relevant**
3. **searched; results found**
4. **results may be of peripheral interest.**

*Include the search terms that were used in your search:*

#

# Health Technology Assessment (HTA) Agencies

## Canada

[The Alberta College of Family Physicians](http://www.acfp.ca/WhoWeAre.aspx) (ACFP). Tools for Practice

<http://www.acfp.ca/WhatWeDo/ToolsforPractice.aspx>

*Filter by "Year" or "Category" using options in the left-hand menu.*

[Language: English]

[Alberta Health and Wellness](http://www.health.alberta.ca/about-us.html). Decision Process provincial reviews – ongoing and complete
<http://www.health.alberta.ca/initiatives/AHTDP-reviews.html>
*Scroll down to view list of completed reports or use the keyword search box in top right corner.*

[Language: English]

[Canadian Agency for Drugs and Technologies in Health](https://www.cadth.ca/about-cadth) (CADTH).

<https://www.cadth.ca/search?keywords>

*Can limit by “Product Line,” “Result Type,” “Publication Date,” etc. in the bar at the left side of the page. Includes projects in progress as well as completed projects.*

[Languages: English, French]

[Drug Safety and Effectiveness Network](http://www.cihr-irsc.gc.ca/e/39389.html)(DSEN).

<http://www.cihr-irsc.gc.ca/e/49006.html>

*For drug topics only. Use Ctrl-F to search keywords on this page. Projects in progress are listed under "Active queries" tab.*

[Languages: English]

[Health Quality Council of Alberta](http://www.hqca.ca/about) (HQCA). Completed Reviews

<http://hqca.ca/studies-and-reviews/completed-reviews/>

[Language: English]

[Health Quality Ontario](http://www.hqontario.ca/about-us) (HQO). Health Technology Assessment

<http://www.hqontario.ca/Evidence-to-Improve-Care/Health-Technology-Assessment>

*Search all publications by entering keywords in the top right-hand search box or browse each of the different report types in the left-hand menu ("Reviews and Recommendations," "Journal: Ontario Health Technology Assessment Series," "Other Publications," etc.) separately. Under Reviews and Recommendations” can browse by health topic or search by entering keywords in the search box.*

[Language: English, French]

[The Hospital for Sick Children (SickKids)](http://www.sickkids.ca/AboutSickKids/who-we-are/index.html). Technology Assessment at SickKids (TASK)

<http://lab.research.sickkids.ca/task/reports-theses/>

[Language: English]

[Institut national d’excellence en santé et en services sociaux](http://www.inesss.qc.ca/en/about-us/about-the-institut.html) (INESSS) [formerly AETMIS]. Publications

<http://www.inesss.qc.ca/en/publications/publications.html>

*If not browsing, may wish to also consider French keywords especially for indications. To search with keywords enter them into search box located under the "Keywords" heading.*

[Languages: French, English]

[Institute of Health Economics](http://www.ihe.ca/about/about-ihe) (IHE). Publications

<http://www.ihe.ca/index.php?/publications>

*For keyword search, use search box in the middle of the page or browse by “Publication Type,” Year of Publication” or “Topics” in left hand menu.*

[Language: English]

[Manitoba Centre for Health Policy](http://www.umanitoba.ca/faculties/medicine/units/community_health_sciences/departmental_units/mchp/about.html) (MCHP). Deliverables

<http://mchp-appserv.cpe.umanitoba.ca/deliverablesList.html>

[Language: English]

[McGill University Health Centre](https://muhc.ca/homepage/page/about-muhc) (MUHC). Technology Assessment Unit Reports

<https://muhc.ca/tau/page/tau-reports>

*Search by date or using search box. See “Work in Progress” tab in left-hand menu for ongoing reports.*

[Language: English]

[NLCAHR : Newfoundland and Labrador Centre for Applied Health Research. Contextualized Health Research Synthesis Program](http://www.nlcahr.mun.ca/CHRSP/EIC.php) (CHRSP) Completed CHRSP projects

<http://www.nlcahr.mun.ca/CHRSP/CompletedCHRSP.php>

*For ongoing projects, click on "Current CHRSP Projects” link in the left menu.*
[Language: English]

[Ottawa Hospital Research Institute](http://www.ohri.ca/ksgroup/) (OHRI). Knowledge Synthesis Group

<http://www.ohri.ca/ksgroup/publications.asp>

*Formerly known as Chalmers Research Group, no longer being updated.*

[Language: English]

[Programs for Assessment of Technology in Health (Canada)](https://www.path-hta.ca/). Reports (PATH)

<https://www.path-hta.ca/research>

[Language: English]

[Therapeutics Initiative](http://www.ti.ubc.ca/About). Therapeutics Letter

<http://www.ti.ubc.ca/TherapeuticsLetter>

*For more evidence-based drug information, click on the "Drug Assessments" tab in the left-hand side menu (drug assessments section very infrequently updated).*[Language: English]

searched; nothing found

[University of British Columbia](http://chspr.ubc.ca/about/chspr). Centre for Health Services and Policy Research

<http://chspr.ubc.ca/publications/>

*This site has a health policy orientation. Can browse by “Topic,” “Type,” “Author,” and “Year”.*

[Language: English]

## International

[INAHTA Secretariat](http://www.inahta.org/about-inahta/). International Network of Agencies for Health Technology Assessment (INAHTA)

<http://www.inahta.org/publications/>

*Search by keyword or browse by product type or authoring agency.*

[Language: English]

[World Health Organization Regional Office for Europe](http://www.euro.who.int/en/data-and-evidence/evidence-informed-policy-making/health-evidence-network-hen). Health Evidence Network (WHO HEN)

<http://www.euro.who.int/en/what-we-do/data-and-evidence/health-evidence-network-hen/publications/by-keyword>

*In site search box, phrases may be searched using quotes " ". A list of evidence reports can be found at:* [*http://www.euro.who.int/en/data-and-evidence/evidence-informed-policy-making/publications/evidence-reports/evidence-reports*](http://www.euro.who.int/en/data-and-evidence/evidence-informed-policy-making/publications/evidence-reports/evidence-reports)

[Languages: English, French, German, Russian]

## Australia

[Australian Government. Department of Health and Ageing](http://www.horizonscanning.gov.au/internet/horizon/publishing.nsf/Content/home-2). Australia and New Zealand Horizon Scanning Network (ANZHSN)

<http://www.horizonscanning.gov.au/internet/horizon/publishing.nsf/Content/technologies-assessed-lp-2>

*Search by keyword or browse by report type (i.e. Horizon Scanning Reports, Prioritising Summaries, or Emerging Technology Bulletins). Contains reports up to 2010.*

[Language: English]

[Australian Government Department of Health and Ageing. Medical Services Advisory Committee](http://www.msac.gov.au/internet/msac/publishing.nsf/Content/about-us-lp-1) (MSAC). MSAC Applications

<http://www.msac.gov.au/internet/msac/publishing.nsf/Content/completed-assessments>

*Search by keyword or application number or browse reports.*

[Language: English]

[Joanna Briggs Institute](http://joannabriggs.org/about.html) (JBI). The Joanna Briggs Institute EBP Database

<http://connect.jbiconnectplus.org/Search.aspx>

*Subscription required for full-text database, available through both JBI COnNECT and OVID interfaces. Able to limit by subject area and publication type.*

[Language: English]

[Monash Health. Centre for Clinical Effectiveness](http://www.monashhealth.org/page/About_Us/) (CCE). Centre for Clinical Effectiveness - Publications

<http://monashhealth.org/health-professionals/cce/cce-publications/>

*Browse "Evidence Reviews" by year on the left-hand side.*

[Language: English]

[National Prescribing Service](http://www.nps.org.au/about_us). NPS RADAR

<https://www.nps.org.au/radar>

*For drug searches only.*

[Language: English]

[Queensland Government (Australia). Health Technology Reference Group](https://www.coaghealthcouncil.gov.au/). Health Technologies Evaluated-Reports and Briefs (COAG Health Council)

<https://www.coaghealthcouncil.gov.au/Health-Technology-Reference-Group/Reports-and-Briefs>

*To search keywords, use the search box in the top right-hand side, or browse by “Health Technology Reports,” “Technology Briefs,” or “Workshop Reports”. Technologies assessed prior to 2011 can be viewed through Horizon Scanning website.* [*http://www.horizonscanning.gov.au/internet/horizon/publishing.nsf/Content/technologies-assessed-lp-2*](http://www.horizonscanning.gov.au/internet/horizon/publishing.nsf/Content/technologies-assessed-lp-2)

[Language: English]

## Austria

[Institute of Technology Assessment](http://www.oeaw.ac.at/ita/en/about-us) (ITA). Projects

<http://www.oeaw.ac.at/ita/en/projects>

[Languages: German, English]

[Ludwig Boltzmann Institut für Health Technology Assessment](http://hta.lbg.ac.at/page/about-us) (LBI). Ludwig Boltzmann Institute of Health Technology Assessment

<http://eprints.hta.lbg.ac.at/>

*Most articles in German with English summaries. Can also browse by “Type” or “Subject “using the left-hand menu.*

[Languages: German, English]

## Belgium

[Kenniscentrum voor de Gezondheidszorg / Le Centre d'expertise des soins de santé](https://kce.fgov.be/en/about-us). Belgian Health Care Knowledge Centre (KCE)

<https://kce.fgov.be/en/all-reports>

*This site contains many English reports (as well as Dutch and French). Can browse by “Publication year,” “General domain,” or “Specific domain”* *using the left-hand menu.*

[Languages: English, French, Dutch]

## Denmark

[Sundhedsstyrelsen. Danish Health and Medicines Authority](http://sundhedsstyrelsen.dk/en/about-us) (DHMA). Publications

<http://sundhedsstyrelsen.dk/en/publications>

*Reports in Danish, but include English summaries.*

[Languages: Danish, English summaries]

## France

[Comité d'Evaluation de Diffusion des Innovations Technologiques](http://cedit.aphp.fr/cedit-hta-agency/) (CEDIT). CEDIT Recommendations and Reports

<http://cedit.aphp.fr/cedit-hospital-based-hta-agency/recommendations-reports/>

*Most reports in French only but recommendations are translated into English.*

[Languages: French, English]

[Haute Autorité de santé/ French National Authority for Health](http://www.has-sante.fr/portail/display.jsp?id=c_5443&pcid=c_5443) (HAS). Haute Autorité de santé

<http://www.has-sante.fr/portail/jcms/c_946986/en/english-toutes-nos-publications-ligne-principale?portal=r_1457306>

*This site is also listed in the "Clinical Practice Guidelines" section of this checklist. Formerly Agence Nationale d'Accréditation et d' Évaluation en Santé. Many reports are translated to English.*

[Languages: French, English]

## Germany

[Deutsches Institut für Medizinische Dokumentation und Information](https://www.dimdi.de/dynamic/en/dimdi/). (DIMDI). German Institute of Medical Documentation and Information

<https://www.dimdi.de/dynamic/en/further-services/health-technology-assessment/hta-reports/>

*Search the DAHTA database in the right-hand side or browse the ongoing HTA reports. Reports in German with English abstracts and summaries.*

[Languages: German, English]

## Ireland

[Health Information and Quality Authority](http://www.hiqa.ie/about-us). Health Technology Assessments

<https://www.hiqa.ie/reports-and-publications/health-technology-assessments>

[Language: English]

[Health Service Executive](http://www.lenus.ie/hse/pages/About Lenus.html). Irish Health Repository **(**Lenus**)**

<http://www.lenus.ie/hse/>

*Use “Advanced Search” at the top right of the page (results can be filtered on the results page) or use the browse tab to browse by “Authors,” “Subject,” etc.*

[Language: English]

## The Netherlands

[De Gezondheidsraad](http://www.gezondheidsraad.nl/en/about-us) (GR). Health Council of the Netherlands

<http://www.gezondheidsraad.nl/en/publications>

*Most articles in Dutch with English summaries. Use search box in the upper-right corner to search the entire site. Use “Search text” box on the right-hand side to search within report title or full-text. On the right-hand side, can also search by “Area of activity,” ”Keywords,” or “Publish date”.*

[Languages: Dutch, English]

[Zorginstituut Nederland](https://www.zorginstituutnederland.nl/). National Health Care Institute Netherlands

<https://english.zorginstituutnederland.nl/publications>

*Formerly College voor zorgverzekeringen (CVZ).*

[Language: Dutch, English]

## Norway

[Folkehelseinstituttet. Norwegian Institute of Public Health](https://www.fhi.no/om/om-fhi/). Publications

<https://www.fhi.no/en/publ/>

[Languages: Norwegian, English]

## Spain

[Agencia de Evaluación de Tecnologías Sanitarias, Instituto de Salud “Carlos III”](http://www.eng.isciii.es/ISCIII/es/contenidos/fd-el-instituto/quienes-somos.shtml). Institute of Health Carlos III

<http://publicaciones.isciii.es/>

*This online publications portal includes reports from the HTA agency "Agencia de Evaluación de Tecnologías Sanitarias (AETS)." Reports in Spanish, occasionally in English.*

[Languages: Spanish, English]

[Agència de Qualitat i Avaluació Sanitàries de Catalunya](http://aquas.gencat.cat/ca/sobre_aquas/) (AQuAS). Agency for Health Quality and Assessment of Catalonia

<http://aquas.gencat.cat/ca/publicacions/>

*Browse by “Medical specialty,” “Year,” or “Type of publication”. Reports in Catalan and Spanish but some have English summaries within the full-text publications.*

[Languages: Catalan, Spanish]

## Sweden

[Sahlgrenska Universitetssjukhuset. Sahlgrenska University Hospital.](https://www.sahlgrenska.se/om-sjukhuset/) Regional activity-based HTA

<https://www.sahlgrenska.se/forskning/htacentrum/>

[Languages: Swedish, English]

[Swedish Council on Health Technology Assessment](http://www.sbu.se/en/About-SBU) (SBU).

<https://www.sbu.se/en/publications/>

*Contains some English reports and others in which only the summaries have been translated. Search for keywords or browse recent reports under “Publications” tab or browse projects in progress under “Ongoing projects” tab.*

[Languages: Swedish, English]

## UK

[Healthcare Improvement Scotland.](http://www.healthcareimprovementscotland.org/about_us.aspx)  Published Resources.

[http://www.healthcareimprovementscotland.org](http://www.healthcareimprovementscotland.org/)

*Under "Access our reports, publications and tools," browse page by subject in right-hand "Healthcare area" box, or use keywords search box to enter keywords. Some overlap with NICE reports (also on checklist).*

[Language: English]

[National Institute for Health and Care Excellence](http://www.nice.org.uk/aboutnice/) (NICE). NHS National Institute for Health and Care Excellence

<http://www.nice.org.uk/>

*For keyword searching, use the search box. To browse by "topic" or "guidance by programme," select “NICE Guidance” tab in the top right-hand of the page.” For individual report, check different tabs in the middle of the page for additional documents (e.g. “Tools and resources” or “Evidence”).*

[Language: English]

[National Institute for Health and Care Excellence](http://www.nice.org.uk/aboutnice/) (NICE). Advice List. Published evidence summaries

<https://www.nice.org.uk/guidance/published>

*For keyword searching, use the search box. Search “Published,” “In consultation,” “In development,” and “Proposed” reports. Can also limit the results by product type in the left-hand menu.*

[Language: English]

[National Institute for Health Research](http://www.io.nihr.ac.uk/what-we-do/). (NIHR). Innovation Observatory

<http://www.io.nihr.ac.uk/>

*Browse by selecting the specialty of interest or use the search box for keyword searching.*

[Language: English]

[NHS Purchasing and Supply Agency](http://nhscep.useconnect.co.uk/Default.aspx). Centre for Evidence-based Purchasing (CEP)

<http://nhscep.useconnect.co.uk/CEPProducts/Catalogue.aspx>

*As of March 31, 2010, the Center has been decommissioned but older reports are still found at the Department of Health (UK) web site.*

[Language: English]

[NIHR Evaluation, Trials and Studies Coordinating Centre](http://www.nets.nihr.ac.uk/about) (NETSCC). Research Project

<https://www.journalslibrary.nihr.ac.uk/programmes/>

*The NETSCC project portfolio has now moved to the NIHR Journals Library website. Use the advanced search option. For search terms/keywords use one or more single words or enclose phrases in "double-quotes."*

[Language: English]

[UK Department of Health](http://www.nric.org.uk/about) (NHS). International Resource for Infection Control (iNRIC)

<http://www.nric.org.uk/resources>

*Search by keyword or browse by condition. Use the filters on the left side of the page to narrow down further.*

[Language: English]

[National Health Service UK](https://www.england.nhs.uk/about/) (NHS). NHS England

<http://www.england.nhs.uk/>

*Contains funding decisions and clinical policies for drugs. Use main site search box.*

[Language: English]

## US

[Agency for Healthcare Research and Quality](http://www.ahrq.gov/cpi/about/index.html) (AHRQ).

- - - - Technology Assessments

<http://www.ahrq.gov/research/findings/ta/>

*Use Ctrl-F to search keywords on this page or use top right-hand search box for less common keywords in order to search the whole site. If older reports (1990-2010) are needed scroll down to the bottom of the screen and click on the "Technology Assessment Archive".*

- - - - Evidence-Based Practice

<http://www.ahrq.gov/research/findings/evidence-based-reports/search.html>

*Use EPC Reports search box to search keywords, or limit by topic, year, etc. In progress reports can be searched separately under “EPC Type” section in the left-hand menu, but they are also included when searching the search box.*

- - - - Effective Health Care Reports

<https://effectivehealthcare.ahrq.gov/products-tools/>

*To search or browse for completed systematic reviews or technical briefs click on “Published Reports” and for reports in development, click on “Reports in Development”.*

[Languages: English, Spanish]

[Centers for Medicare & Medicaid Services](http://www.cms.gov/About-CMS/About-CMS.html) (CMS). Technology Assessments

<http://www.cms.gov/medicare-coverage-database/indexes/technology-assessments-index.aspx?TAId=85&bc=AAAQAAAAAAAA&>

*Change "View Items Per Page" to 100 and use Ctrl-F to search page. Some duplication with AHRQ site.*

[Language: English]

[ECRI Institute](http://www.ecri.org/About/Pages/default.aspx).

<http://www.ecri.org/>

*Subscription required.*

[Language: English]

[Institute for Clinical and Economic Review](http://www.icer-review.org/about/) (ICER).

<https://icer-review.org/materials/>

*Use search box to search keywords or browse reports. Filter by: “Topic,” "Material Type" or "Program Type" (in the middle of the page).* ***CTAF*** *has been consolidated with ICER.*

[Language: English]

[Washington State Health Care Authority](https://www.hca.wa.gov/about-hca) (HCA). Health Technology Review

<https://www.hca.wa.gov/about-hca/health-technology-assessment/health-technology-reviews>

[Language: English]

# Health Economics

## Canada

[Hospital for Sick Children [Toronto]](http://pede.ccb.sickkids.ca/pede/about.jsp). Paediatric Economic Database Evaluation (PEDE)

<http://pede.ccb.sickkids.ca/pede/search.jsp>

*This database contains over 2000 citations of full economic evaluations.*

[Language: English]

[Institute of Health Economics (IHE). Publications](https://www.ihe.ca/about/about-ihe)

<http://www.ihe.ca/index.php?/publications>

*For keyword search, use search box in the middle of the page or browse by “Result Type,” Years” or “Topics” in left hand menu. This site is also listed in the “Health Technology Assessment Agencies” category.* [Language: English]

[McMaster University](http://www.chepa.org/who-we-are). Centre for Health Economics and Policy Analysis. Publications database (CHEPA)

<http://www.chepa.org/research-products>

*Click on "Working Papers" in the left-hand menu for reports not in traditional journal literature.*

[Language: English]

[Public Health Agency of Canada](http://www.phac-aspc.gc.ca/about_apropos/index-eng.php) (PHAC). Economic Burden of Illness in Canada

<http://www.phac-aspc.gc.ca/ebic-femc/index-eng.php>

*Search only if burden of illness statistics required. Download the pdf document and perform a keyword search within.*

[Languages: English, French]

[Toronto Health Economics and Technology Assessment Collaborative](http://theta.utoronto.ca/content.php?pid=411861&sid=3372324) (THETA).THETA Publications and Knowledge Translation to Policy (KT) Activities

<http://theta.utoronto.ca/content.php?pid=411861&sid=3372336>
*To search this site click on the following sections and browse each page: "Publications” and "Reports”. The keyword search box in the upper right-hand margin does not retrieve all reports.*

[Language: English]

## International

[Agency for Healthcare Research and Quality](http://www.qualitymeasures.ahrq.gov/about/index.aspx) (AHRQ). National Quality Measures Clearinghouse

<https://www.ahrq.gov/gam/index.html>

*Contains information on specific evidence-based health care quality measures and measure sets.*

[Language: English]

[Australian Government Department of Health](http://www.pbs.gov.au/info/about-the-pbs). Pharmaceutical Benefits Scheme - Medicine Listing (PBS)

<http://www.pbs.gov.au/browse/medicine-listing>

*Browse alphabetical list for drug pricing.*

[Language: English]

[Federal Reserve Bank of St. Louis. Economic Research Division](http://repec.org/). Ideas database (IDEAS)

<http://ideas.repec.org/>

*Limit the search to "Papers," "Chapters," and "Books" (articles are already captured in database searches). NEED TO SCROLL DOWN the page to see search results.*

[Language: English]

[International Society for Pharmacoeconomics and Outcomes Research](https://www.ispor.org/about/our-mission) (ISPOR). Value in Health: Journal of the International Society for Pharmacoeconomics and Outcomes Research

<http://www.valueinhealthjournal.com/issues>

*Subscription required for some content. Use keyword search in top menu. Includes abstracts from ISPOR's annual conferences. Duplication with EMBASE database which also indexes these conference abstracts.*

[Language: English]

[National Centre for Pharmacoeconomics.](http://www.ncpe.ie/about/) (NCPE) Ireland. Pharmacoeconomic Evaluations

<http://www.ncpe.ie/pharmacoeconomic-evaluations/>

*Select the technology type of interest (i.e. Drugs) and browse using the alphabetical listing. Summaries not available for all technologies.*

[Language: English]

NHS Economic Evaluation Database (EED), economic evaluations of

health care interventions

*Searchable as part of the University of York* [*NHS CRD databases*](https://www.crd.york.ac.uk/CRDWeb/Homepage.asp)*.* *See the databases section.*

[Language: English]

[University of Aberdeen](http://www.abdn.ac.uk/heru/about/). Health Economics Research Unit (HERU)

<http://www.abdn.ac.uk/heru/outputs/publications/>

*Select publication types "HERU Briefing Papers" and "Reports" and browse by year within each category. Full-text links are not available for all reports.*

[Language: English]

# Clinical Practice Guidelines

## Canada

[Alberta Medical Association](http://www.topalbertadoctors.org/about-top/). Toward Optimized Practice (TOP)

<http://www.topalbertadoctors.org/cpgs.php?sid=1>

*Type keywords into the "CPG Search" box located in the left-hand menu.*

[Language: English]

[British Columbia Ministry of Health](http://www2.gov.bc.ca/gov/content/health/practitioner-professional-resources/bc-guidelines/about-the-guidelines). BC Guidelines

<http://www2.gov.bc.ca/gov/content/health/practitioner-professional-resources/bc-guidelines>

*Can browse by “Topic” or “Alphabetical Listing” from left-hand menu.*

[Language: English]

[Canadian Medical Association](https://www.cma.ca/about-cma) (CMA). CMA Infobase: Clinical Practice Guidelines

<https://www.cma.ca/En/Pages/clinical-practice-guidelines.aspx>

*To search full text (not just title), check off "Include full text in search" box below the search box. Can also browse by "Condition" or "Speciality" in the middle of the page.*

[Languages: English, French]

[Canadian Partnership Against Cancer](https://www.partnershipagainstcancer.ca/about-us/). Cancer Guidelines Database

<https://www.partnershipagainstcancer.ca/tools/cancer-guidelines-database/>

*Only search for cancer topics. To search keywords use search box or browse by “Type of cancer” or “Continuum of care”.*

[Language: English]

[Canadian Standards Association](https://www.csagroup.org/about-csa-group/) (CSA). Occupational Health and Safety

<http://shop.csa.ca/en/canada/products/occupational-health-and-safety/icat/ohs/&bklist=icat,4,shop,publications,ohs>

[Languages: English, French]

[The College of Physicians and Surgeons of Ontario](http://www.cpso.on.ca/About-Us) (CPSO). CPGs & Other Guidelines

<http://www.cpso.on.ca/Policies-Publications/CPGs-Other-Guidelines> [Language: English]

[Ontario Association of Medical Laboratories](https://oaml.com/) (OAML)

<https://oaml.com/guidelines/>

*Lab test guidelines; those marked with an asterisk are also in the CMA Infobase guideline repository.*

[Language: English]

[Public Health Agency of Canada](http://www.phac-aspc.gc.ca/about_apropos/index-eng.php) (PHAC). Disease Prevention and Control Guidelines

<http://www.phac-aspc.gc.ca/dpg-eng.php>

[Languages: English, French]

[Registered Nurses' Association of Ontario](http://rnao.ca/about) (RNAO). Nursing Best Practice Guidelines

<http://rnao.ca/bpg>

*To search by keyword use the search box in the middle of the page or to browse, click on “Browse by Topic” tab.*

[Language: English]

[University of Ottawa. School of Rehabilitation Science](http://www.health.uottawa.ca/rehabguidelines/en/login.php). Evidence-based Practice

<http://www.health.uottawa.ca/rehabguidelines/en/search.php>

[Languages: English, French]

[Winnipeg Regional Health Authority](http://www.wrha.mb.ca/about/aboutus.php) (WRHA). Evidence Informed Practice Tools

<http://www.wrha.mb.ca/professionals/ebpt/>

[Language: English]

##

## International

[Academy of Medicine of Malaysia](http://www.acadmed.org.my/index.cfm?&menuid=9). Clinical Practice Guidelines

<http://www.acadmed.org.my/index.cfm?&menuid=67>

*See also "General Guidelines" link in the left-hand menu.*

[Language: English]

[Aetna, Inc.](http://www.aetna.com/about-aetna-insurance/index.html) Clinical Policy Bulletins

- - - - <http://www.aetna.com/healthcare-professionals/policies-guidelines/medical_clinical_policy_bulletins.html> **(medical)**

*Scroll down the page for the CPB (Medical Clinical Policy Bulletin) search box or alternatively browse the "Alphabetical List" to see topics in alphabetical order.*

- - - - <http://www.aetna.com/healthcare-professionals/policies-guidelines/pcpb_menu.html> **(pharmaceutical)**

*Only bulletins from last one-two years (most recent) are available.*

[Language: English]

[American Association for Clinical Chemistry](https://www.aacc.org/about-aacc) (AACC). Practice Guidelines

[https://www.aacc.org/science-and-research/practice-guidelines#](https://www.aacc.org/science-and-research/practice-guidelines)

[Language: English]

[Best Practice Advocacy Centre New Zealand](http://www.bpac.org.nz/AboutUs.aspx) (bpacNZ). bpacNZ better medicine

<http://www.bpac.org.nz/Default.aspx>

*Especially useful for laboratory tests.*

[Language: English]

[Centers for Disease Control and Prevention](http://www.cdc.gov/about/) (CDC). *Public Health Genomics Knowledge Base. Guideline Database* <https://phgkb.cdc.gov/PHGKB/evidencerStartPage.action>

[Language: English]

[The Regulation and Quality Improvement Authority](https://rqia.org.uk/who-we-are/about-rqia/about-us-information-leaflet/) (RQIA). Guidelines

<https://rqia.org.uk/what-we-do/rqia-clinical-audit-programme/guidelines/> [Language: English]

[Haute Autorité de santé/ French National Authority for Health](http://www.has-sante.fr/portail/display.jsp?id=c_5443&pcid=c_5443) (HAS). Practice Guidelines

<http://www.has-sante.fr/portail/jcms/c_6056/en/recherche-avancee?portlet=c_39085&search_antidot=&lang=en&typesf=guidelines>
*Search box on top right-hand side will retrieve all reports (not just guidelines); to limit results to guidelines, select “Content type" tab in the left-hand menu then limit to “Practice Guidelines and Other Guides." This site is also searched in the “HTA Agency International” section of this checklist. Many reports are translated into English.*

[Languages: French, English]

[Institute for Clinical Systems Improvement](https://www.icsi.org/about_icsi/) (ICSI). Guidelines

<https://www.icsi.org/guidelines__more/>

*Reports available for purchase or to members only.*
[Language: English]

[ECRI Institute](http://www.ecri.org/About/Pages/default.aspx)**.** ECRI Guidelines Trust **(**ECRI**)**

<https://guidelines.ecri.org/>

*Need to register to access.*

[Languages: English]

[National Health and Medical Research Council](https://www.clinicalguidelines.gov.au/about.php) (NHMRC). Australia’s Clinical Practice Guidelines Portal

<http://www.clinicalguidelines.gov.au/>

*For completed guidelines search "Guideline Portal Search" box. For ongoing guidelines search "Guideline Register Search" box.*

[Language: English]

[National Institute for Health and Care Excellence](http://www.nice.org.uk/about) (NICE). NICE Guidelines

<http://www.nice.org.uk/guidance>

*This site is also searched in the HTA International section of this checklist. For keyword searching, use the search box. To browse by "topic" or "guidance by programme," select “NICE Guidance” tab in the top right-hand of the page.” For individual report, check different tabs in the middle of the page for additional documents (e.g. “Tools and resources” or “Evidence”).*

[Language: English]

[Scottish Intercollegiate Guidelines Network](http://www.sign.ac.uk/who-we-are.html) (SIGN)

<http://www.sign.ac.uk/our-guidelines.html>

*Search for completed guidelines under "Current guidelines". Click "Guidelines in development" or "Current proposals" in the left-hand menu for guidelines in development or topic proposals.*

[Language: English]

# Drug and Device Regulatory Approvals

## Canada

[Health Canada](http://www.hc-sc.gc.ca/ahc-asc/index-eng.php). ***Drugs***

- - - - Drugs and Health Products: Drug Product Database

<https://health-products.canada.ca/dpd-bdpp/index-eng.jsp>

*Contains drug product monographs for drugs approved for sale in Canada.*

- - - - Drugs and Health Products: Notice of Compliance Database

<https://health-products.canada.ca/noc-ac/index-eng.jsp>
*To verify if a drug has been approved in Canada.*

- - - - Drugs and Health Products: Patent Register

<http://www.hc-sc.gc.ca/dhp-mps/prodpharma/patregbrev/index-eng.php>

*Listing of medicinal ingredients, their associated patents, and the Canadian patent expiry dates.*

- - - - Summary Basis of Decision: Drugs (SBD)

<https://hpr-rps.hres.ca/reg-content/summary-basis-decision-result.php?lang=en&term>

*For keyword searching, use search box under “Filter items” or Select “All” under “Show entries” and use find feature (Ctrl-F) and search by drug name.*

[Languages: English, French]

[Health Canada](http://www.hc-sc.gc.ca/ahc-asc/index-eng.php). ***Devices***

- - - - Medical Devices Active Licence Listing (MDALL)

<https://health-products.canada.ca/mdall-limh/index-eng.jsp>

*Select “Active Licence Search” for class 2 or higher medical devices approved for sale in Canada.*

- - - - Summary Basis of Decision: Medical Devices (SBD)

<https://hpr-rps.hres.ca/reg-content/summary-basis-decision.php>
*Search keyword in the search box in the middle of the page.*

[Languages: English, French]

## International

[Australian Government Department of Health. Pharmaceutical Benefits Scheme](http://www.pbs.gov.au/info/about-the-pbs). Public Summary Documents by Product

<http://www.pbs.gov.au/info/industry/listing/elements/pbac-meetings/psd/public-summary-documents-by-product>

[Language: English]

[Australian Government Department of Health. Therapeutic Goods Administration](http://www.tga.gov.au/about-tga). Australian Public Assessment Reports for Prescription Medicines (AusPARs)

<http://www.tga.gov.au/ws-auspar-index>

*For keyword searching, use search box in the middle of the page. Search by product name, active ingredient or sponsor.*

[Language: English]

[Department of Health (UK)](https://www.gov.uk/government/organisations/medicines-and-healthcare-products-regulatory-agency/about). Medicines and Healthcare Products Regulatory Agency (MHRA)

<https://www.gov.uk/government/organisations/medicines-and-healthcare-products-regulatory-agency>

[Language: English]

[European Medicines Agency](https://www.ema.europa.eu/en/about-us) (EMA).

- - - - European Public Assessment Reports (EPAR)

<https://www.ema.europa.eu/en/medicines/field_ema_web_categories%253Aname_field/Human/ema_group_types/ema_medicine>

*For keyword searching, use the search box.*

- - - - Pending EC Decisions

<https://www.ema.europa.eu/medicines/field_ema_web_categories%253Aname_field/Human/ema_group_types/ema_smops>

*For keyword searching, use the search box.*

[Language: English]

[NHS Scotland](http://www.scottishmedicines.org.uk/About_SMC/What_we_do/index). Scottish Medicines Consortium (SMC)

<https://www.scottishmedicines.org.uk/medicines-advice/>

*For keyword searching, use the search box in the middle of the page. For in progress advices, click on the “Under consideration” tab. Advanced search provides more options.*

[Language: English]

not searched; not relevant [Pharmaceutical Management Agency of New Zealand](http://www.pharmac.health.nz/about). PHARMAC

<http://www.pharmac.health.nz/>

*For keyword searching, use search box in the top right corner. Use "Website" and "Schedule" tabs to search.*

[Language: English]

not searched; not relevant [US Food and Drug Administration](http://www.fda.gov/AboutFDA/Transparency/Basics/ucm192695.htm) (FDA). ***Devices***

- - - - Product Classification

<https://www.accessdata.fda.gov/scripts/cdrh/cfdocs/cfpcd/PCDSimpleSearch.cfm>

*This database contains device names and their associated product codes. Search by device type (e.g. stent) or go to “Advanced Search” for more options.*

- - - - 510(k) Premarket Notification

<http://www.accessdata.fda.gov/scripts/cdrh/cfdocs/cfPMN/pmn.cfm>

*U.S. device clearance notification site for class I, II & some III medical devices, for which a Premarket Approval application (PMA) is not required.*

- - - - 513(k) De Novo Device Classification

<https://www.accessdata.fda.gov/scripts/cdrh/cfdocs/cfpmn/denovo.cfm>

*Alternate approval pathway for novel low-moderate risk medical devices.*

- - - - Premarket Approval (PMA)

<https://www.accessdata.fda.gov/scripts/cdrh/cfdocs/cfpma/pma.cfm>

*U.S. device approval notification site for class III medical devices.*

- - - - Devices@FDA

<http://www.accessdata.fda.gov/scripts/cdrh/devicesatfda/index.cfm>

*Devices@FDA searches the PMN-510(k) Premarket Notification and PMA-Premarket Approval FDA databases.*

[Language: English]

not searched; not relevant [US Food and Drug Administration](http://www.fda.gov/AboutFDA/Transparency/Basics/ucm192695.htm) (FDA). ***Drugs***

- - - - Advisory Committees

<http://www.fda.gov/oc/advisory/default.htm>

*Type drug name in search box, take note of any relevant Advisory Committee names and dates, then go back to homepage and browse by that particular meeting minutes to find relevant documents.*

- - - - Drugs@FDA: FDA Approved Drug Products

<http://www.accessdata.fda.gov/scripts/cder/drugsatfda/>

*Search drug name, select the appropriate route of administration (if applicable), click on approval history, then link to review for medical and/or statistical information, etc. If medical and statistical reviews NOT available in the approval package, search Google to double check if not posted on a different page.*

- - - - Guidances (Drugs) <http://www.fda.gov/Drugs/GuidanceComplianceRegulatoryInformation/Guidances/default.htm>

*Contains FDA guidance documents on drugs. Use “Search all FDA Guidance Documents” search box in the middle of the page and change “Product” drop-down option to “Drugs”.*

[Language: English]

# Advisories and Warnings

## Canada

[Canadian Pharmacists Association](http://www.pharmacists.ca/index.cfm/about-cpha/) (CPhA). e-CPS

<https://www.e-therapeutics.ca/>

*This database contains approved Canadian pharmaceutical product monographs, requires a paid subscription to e-Therapeutics.*

[Language: English]

[Health Canada](http://www.hc-sc.gc.ca/ahc-asc/index_e.html).

- - - - Healthy Canadians Recalls & Alerts

<http://www.healthycanadians.gc.ca/recall-alert-rappel-avis/search-recherche/advanced-avancee/en>

*Choose "Health products" in the category drop-down menu.*

- - - - Health Product InfoWatch

<https://www.canada.ca/en/health-canada/services/drugs-health-products/medeffect-canada/health-product-infowatch/published-newsletters.html>

*Use find feature (Ctrl-F) and search by drug name for newsletters published prior to 2015. For newsletters published after 2014, adverse reactions are reported under "Monthly recap" link for each month. Formerly the Canadian Adverse Reaction Newsletter (CARN).*

- - - - Canada Vigilance Adverse Reaction Online Database

<http://cvp-pcv.hc-sc.gc.ca/arq-rei/index-eng.jsp>

*To search the database, fill in the appropriate criteria and then scroll down to click the "Search" button. There is an option to "Export Results" from the report page.*

[Languages: English, French]

##

## International

[Australian Government Department of Health. Therapeutic Goods Administration](http://www.tga.gov.au/about-tga) (TGA).

- - - - Australian Adverse Drug Reactions Bulletin

<http://www.tga.gov.au/australian-adverse-drug-reactions-bulletin-article-index>

*Has been replaced by the Medicines Safety Update, but still has reports from 1982-2009.*

- - - - Medical Device Incident Reporting & Investigation Scheme (IRIS) articles

<http://www.tga.gov.au/publication/medical-device-incident-reporting-investigation-scheme-iris-articles>

*Has been replaced by the Medical Devices Safety Update, but still has reports from 2001-2012.*

- - - - Medical Devices Safety Update

<http://www.tga.gov.au/publication/medical-devices-safety-update>

*Replaces the Medical Device Incident Reporting & Investigation Scheme.*

- - - - Medicines Safety Update

<http://www.tga.gov.au/publication/medicines-safety-update>

*Use Ctrl-F to search page. Replaces the Australian Adverse Drug Reactions Bulletin which has issues from 1982-2009.*

[Language: English]

##

[European Medicines Agency](http://www.ema.europa.eu/ema/index.jsp?curl=pages/about_us/document_listing/document_listing_000426.jsp&mid=WC0b01ac058001ce7d) (EMA).

- - - - European Database of Suspected Adverse Drug Reaction Reports

<http://www.adrreports.eu/en/index.html>
*Summarized adverse event reports. Flash must be installed to view summaries. Results can be saved as PDF or Excel file.*

- - - - Patient Safety

<https://www.ema.europa.eu/en/medicines/field_ema_web_categories%253Aname_field/Human/ema_group_types/ema_medicine/ema_medicine_patient_safety/1>

*Use Ctrl-F and search by drug name.*

- - - - Withdrawn Applications

<https://www.ema.europa.eu/en/medicines/field_ema_web_categories%253Aname_field/Human/ema_group_types/ema_medicine/field_ema_med_status/refused-38/field_ema_med_status/withdrawn-40/field_ema_med_status/withdrawn-application-41>

*Search by the drug name or browse.*

[Language: English]

[Medicines and Healthcare Products Regulatory Agency](https://www.gov.uk/government/organisations/medicines-and-healthcare-products-regulatory-agency/about) (MHRA). Drug Safety Update

<https://www.gov.uk/drug-safety-update>

*Enter keywords into search box on left-hand side of the page or limit by “Therapeutic area” and date.*

[Language: English]

[NHS England. Patient Safety Domain](http://www.england.nhs.uk/about/). Patient Safety Alerts (NRLS)

<https://improvement.nhs.uk/news-alerts/?keywords=&articletype=patient-safety-alert&after=&before=>

*To view safety alerts only, click on "Article type" drop-down on the left-hand side and select "Patient safety alerts". Alerts prior to 2013 are archived at* [*http://www.nrls.npsa.nhs.uk/alerts/*](http://www.nrls.npsa.nhs.uk/alerts/)*.*

[Language: English]

[New Zealand Medicines and Medical Devices Safety Authority](http://www.medsafe.govt.nz/other/about.asp).

- - - - Medsafe: Medical Devices

<http://www.medsafe.govt.nz/profs/device-issues.asp>

*This page provides information on current safety issues related to medical devices in New Zealand. Click on the "Archived Medical Devices Issues" link for older safety issues concerning medical devices.*

- - - - Medsafe: Prescriber Update Articles

<http://www.medsafe.govt.nz/publications/prescriber-update-index.asp>

*Use ‘find’ function (Ctrl-F) in your browser to locate safety issues on a particular medicine or class.*

[Language: English]

[US Food and Drug Administration](http://www.fda.gov/AboutFDA/Transparency/Basics/ucm192695.htm) (FDA).

- - - - MAUDE - Manufacturer and User Facility Device Experience

<http://www.accessdata.fda.gov/scripts/cdrh/cfdocs/cfmaude/search.cfm>

*Search by device's brand name.*

- - - - MedWatch: FDA Safety Information and Adverse Event Reporting Program

<https://www.fda.gov/Safety/MedWatch/SafetyInformation/default.htm>

*Alerts prior to 2017 are archived at:* [*http://wayback.archive-it.org/7993/20170110235327/http://www.fda.gov/Safety/MedWatch/SafetyInformation/default.htm*](http://wayback.archive-it.org/7993/20170110235327/http://www.fda.gov/Safety/MedWatch/SafetyInformation/default.htm)*.*

[Language: English]

# Drug Class Reviews

[Department of Veterans Affairs (US). Pharmacy Benefits Management Services](http://www.va.gov/about_va/). Drug Class Reviews

<http://www.pbm.va.gov/clinicalguidance/drugclassreviews.asp>
*Browse the drug class reviews list or type a keyword into the search box on the top right-hand menu to search the whole web site.*

[Language: English]

[Oregon Health & Science University. Center for Evidence-based Policy](https://centerforevidencebasedpolicy.org/). Drug Effectiveness Review Project (DERP)

<https://www.ncbi.nlm.nih.gov/books/NBK10527/toc/?report=reader>

*Browse the drug class reviews list.*

[Language: English]

[Saskatoon Health Region](http://www.rxfiles.ca/rxfiles/modules/aboutus/aboutus.aspx). RxFiles: Objective Comparisons for Optimal Drug Therapy

<http://www.rxfiles.ca/rxfiles/modules/druginfoindex/druginfo.aspx>

*Use the search box to search the whole web site. Subscription required.*

[Language: English]

# Clinical Trials Registries

[Biomed Central](http://www.isrctn.com/page/about). ISRCTN Registry

<http://www.isrctn.com/>

*Browse by medical topic or use the advanced search at the top right of the screen to search in specific fields such as funder, country, or participant gender.*

[Language: English]

[Canadian Partnership Against Cancer Corporation](http://www.canadiancancertrials.ca/aboutus.aspx?). Canadian Cancer Trials

<http://www.canadiancancertrials.ca/>

*For cancer topics only; most trials also in Clinicaltrials.gov.*

[Language: English and French]

[National Institute of Medical Statistics, Indian Council of Medical Research](http://ctri.nic.in/Clinicaltrials/login.php). Clinical Trials Registry - India (CTRI)

<http://ctri.nic.in/Clinicaltrials/advancesearchmain.php>

[Language: English]

[Thomson CenterWatch](http://www.centerwatch.com/about-centerwatch/index.htm). CenterWatch Clinical Trials Listing Service

<http://www.centerwatch.com/clinical-trials/listings/>

*For keyword search, use the search box. Browse by “Medical Condition” or “Therapeutic Area” tabs.*

[Language: English]

[US National Institutes of Health](https://clinicaltrials.gov/ct2/about-site/background). ClinicalTrials.gov

<http://clinicaltrials.gov/ct/screen/AdvancedSearch>
[Language: English]

[UK Department of Health](http://www.ukcrc.org/research-infrastructure/clinical-research-networks/uk-clinical-research-network-ukcrn/). UK Clinical Research Network Study Portfolio (UKCRN)

<http://www.ukcrc.org/research-infrastructure/clinical-trials-units/>

[Language: English]

[World Health Organization](http://www.who.int/ictrp/about/en/). International Clinical Trials Registry Platform Search Portal (ICTRP)

<http://www.who.int/trialsearch/>

*Use "Advanced Search" screen to limit results. Ignore trials with ID numbers beginning with "NCT," if you have already searched for trials from Clinicaltrials.gov.*

[Language: English]

# Canadian Drug Formularies

[Government of Canada](https://www.canada.ca/en/government/about.html). Prescription Drug Insurance Coverage

<https://www.canada.ca/en/health-canada/services/health-care-system/pharmaceuticals/access-insurance-coverage-prescription-medicines.html>

*Portal to Canadian drug plans, this site can be used to access different provincial / territorial and federal drug benefit programs. The federal drug benefit programs are listed under “Federal public drug benefit programs” link. The provincial and territorial drug plans are listed under “Provincial and territorial public drug benefit program link.*

[Languages: English, French]

# Canadian Physician Fees Schedules

[Alberta Health. Government of Alberta](http://www.health.alberta.ca/about-us.html). Fees Information for Health Professionals

<https://www.alberta.ca/fees-health-professionals.aspx>

[Language: English]

[Ministry of Health Services](https://www2.gov.bc.ca/gov/content/governments/organizational-structure/ministries-organizations/ministries/health). Province of British Columbia**.** Medical Services Commission Payment Schedule

<https://www2.gov.bc.ca/gov/content/health/practitioner-professional-resources/msp/physicians/payment-schedules/msc-payment-schedule> [Languages: English]

[Manitoba Health, Seniors and Active Living](https://www.gov.mb.ca/health/about.html). Government of Manitoba**.** Physician's Manual

<http://www.gov.mb.ca/health/manual/index.html>

[Language: English, French]

[New Brunswick Department of Health](http://www2.gnb.ca/content/gnb/en/departments/health.html). Physician’s Manual

<http://www2.gnb.ca/content/gnb/en/departments/health/healthprofessionals.html>

*Physician’s manual link located in the middle of the page under “Medicare”.*

[Language: English, French]

[Department of Health and Community Services](http://www.health.gov.nl.ca/health/department/index.html). Government of Newfoundland and Labrador**.** Information for Medical Care Plan (MCP) Providers

<http://www.health.gov.nl.ca/health/mcp/providers/index.html>

*Click on "Medical Care Plan (MCP) Medical Payment Schedule" link for fees.*

[Language: English]

[College of Physicians and Surgeons of Nova Scotia](https://cpsns.ns.ca/about-us/). Information and Services for Physicians Licensed in Nova Scotia

<https://cpsns.ns.ca/registration-licensing/fee-schedule/>

[Language: English]

[Ministry of Health and Long-Term Care](http://www.health.gov.on.ca/en/). Ontario Health Insurance (OHIP) Schedule of Benefits and Fees <http://www.health.gov.on.ca/english/providers/program/ohip/sob/sob_mn.html>

[Language: English]

[Régie de l'assurance maladie du Québec](http://www.ramq.gouv.qc.ca/en/regie/Pages/mission.aspx). Physician’s Manual – Fee for service

<http://www.ramq.gouv.qc.ca/fr/professionnels/medecins-omnipraticiens/manuels/Pages/facturation.aspx>

[Language: French]

[Ministry of Health, Government of Saskatchewan](https://www.saskatchewan.ca/government/government-structure/ministries/health). Physician Payment Schedules

[http://www.saskatchewan.ca/government/health-care-administration-and-provider-resources/resources-for-health-care-businesses-and-career-development/physician-career-resources#physician-paymmedicaent-schedules-newsletters-and-bulletins](http://www.saskatchewan.ca/government/health-care-administration-and-provider-resources/resources-for-health-care-businesses-and-career-development/physician-career-resources#physician-paymmedicaent-schedules-newsletters-and-bulletins)

[Language: English]

[Government of Yukon](http://www.hss.gov.yk.ca/mission.php). Yukon Health and Social Services

<http://www.hss.gov.yk.ca/paymentschedule.php>

*Click on "Online Pay Schedule for Yukon" link for fees.*

[Language: English]

# Databases (free)

*These databases are not limited to the retrieval of grey literature. Many, such as PubMed, retrieve traditional commercially published literature.*

*Subject-specific databases, as well as databases that focus on a particular product type (ex. guidelines, health technology assessments), are listed under the relevant categories (see Table of Contents).*

[Bandolier](http://www.bandolier.org.uk/). Bandolier Knowledge <http://www.bandolier.org.uk/knowledge.html>

[Language: English]

[Latin-American and Caribbean Center on Health Sciences Information](http://lilacs.bvsalud.org/en/) (LILACS)

<http://bases.bireme.br/cgi-bin/wxislind.exe/iah/online/?IsisScript=iah/iah.xis&base=LILACS&lang=I>

*LILACS is searched via the “Virtual health library” (VHL) portal and contains documents in Spanish, Portuguese, and English.*

[Languages: English, Portuguese, Spanish]

[McMaster University, McMaster Health Forum](https://www.healthsystemsevidence.org/about?). Health Systems Evidence

[http://www.healthsystemsevidence.org](http://www.healthsystemsevidence.org/)

*Registration is free to access this database. Contents include research evidence as well as Canadian health policy documents from: Canada's Evidence-Informed Healthcare Renewal (EIHR) Portal, Intergovernmental Organizations' Health Systems Documents Portal, and the Ontario Health System Documents Portal.*

[Language: English]

[National Center for Biotechnology Information](https://www.ncbi.nlm.nih.gov/home/about/) (NCBI). Bookshelf

<http://www.ncbi.nlm.nih.gov/books>

[Language: English]

[National Institute for Health and Care Excellence](http://www.nice.org.uk/About/What-we-do/Evidence-Services/Evidence-Search) (NICE). Evidence Search: Health and Social Care

<http://www.evidence.nhs.uk/>

*Some overlap with National Institute for Health and Care Excellence (NICE) and other UK NHS sites including Cochrane Library.*

[Language: English]

[TRIP Database](http://www.tripdatabase.com/about) (TRIP). Trip Database - Clinical Search Engine

<http://www.tripdatabase.com/>

*Free sign up required to access most results, paid membership required to access full content. On results page, use filters on right-hand side to refine initial search; those items highlighted with green colour are considered higher quality evidence. There is a lot of overlap with other evidence-based Web sites.*

[Language: English]

[University of York](http://www.york.ac.uk/crd/about/) (CRD). Centre for Reviews and Dissemination

<http://www.crd.york.ac.uk/crdweb/SearchPage.asp>

*CRD databases include Database of Abstracts of Reviews of Effects (DARE), Health Technology Assessment (HTA) database, and NHS Economic Evaluation Database (NHS EED). DARE and NHS EED are no longer updated as of March 2015. As of March 31, 2018, CRD is no longer adding new records to the HTA database (until a new database platform becomes available).*

[Language: English]

[University of York](https://www.crd.york.ac.uk/prospero/#aboutpage). PROSPERO: International prospective register of systematic reviews

<http://www.crd.york.ac.uk/prospero/>

*Registry of incomplete, ongoing systematic reviews.*

[Language: English]

# [US National Library of Medicine](http://www.nlm.nih.gov/about/index.html) (NLM). PubMed.

# <http://www.ncbi.nlm.nih.gov/pubmed>

*Biggest biomedical database in the world, not considered grey literature.*

[Language: English]

[US National Library of Medicine & National Institutes of Health](http://www.ncbi.nlm.nih.gov/pmc/about/intro/) (NIH). PubMed Central

<http://www.ncbi.nlm.nih.gov/pmc/>

*Duplicate of articles retrieved through PubMed but allows for full-text searching for those articles indexed within PubMed Central.*

# [Language: English]

# Databases (subscription-based)

[Wiley InterScience](http://www.cochrane.org/about-us). Cochrane Library

<http://onlinelibrary.wiley.com/o/cochrane/cochrane_search_fs.html>

*Library of Cochrane Systematic Reviews can be searched for free but requires a subscription for the full-text reports (some jurisdictions may provide free access to residents). Can also search University of York CRD databases through this portal.*

[Language: English]

[UpToDate](http://www.uptodate.com/home/about-us). UpToDate.com

<http://www.uptodate.com/online/login.do>

*Subscription required for this database of evidence based medicine information.*

[Language: English]

[Synergus](http://synergus.com/about). HTA Update

<https://synergus.onelogin.com/login>

*Subscription required.*

[Language: English]

# Health Statistics

## Canada

[Canadian Institute for Health Information](https://www.cihi.ca/en/about-cihi) (CIHI). Quick Stats

<https://www.cihi.ca/en/quick-stats>

*Browse each page or search using the search box in the middle of the page.*

[Languages: English, French]

[Health Canada](http://www.hc-sc.gc.ca/ahc-asc/index-eng.php). Health Canada: Advanced Search

<http://recherche-search.gc.ca/rGs/s_r?as_q=&as_epq=&as_oq=&as_eq=&1s_f3l2typ2=&as_nlo=&as_nlh&as_occt=&1s_s3t2s21rch=&1s_s4rt=&st1rt=0&st=a&num=10&langs=eng&cdn=health>

*Contains some statistical information on health in Canada.*

[Languages: English, French]

not searched; not relevant

[IQVIA](https://www.iqvia.com/about-us).

<https://www.iqvia.com/>

*Formerly IMS Health and Quintiles.*

[Language: English]

not searched; not relevant

[Institute for Clinical Evaluative Sciences](http://www.ices.on.ca/About-ICES.aspx) (ICES). Publications

<http://www.ices.on.ca/Publications.aspx>

*Use search box in top right-hand corner of the screen or use sidebar on right side of the page to limit by publication type, topic, and date.*

[Language: English]

[Institute of Health Economics](http://www.ihe.ca/about/about-ihe) (IHE). Database of Online Health Statistics

<http://www.ihe.ca/health-statistics-database>

*This database covers freely available web-based health statistics. Current focus is on Canadian and American statistics, but there is some international coverage.*

[Language: English]

[New Brunswick Ministry of Health, Office of the Chief Medical Officer of Health](http://www2.gnb.ca/content/gnb/en/departments/ocmoh/about_us.html). Epidemiology and Surveillance

<http://www2.gnb.ca/content/gnb/en/departments/ocmoh/epidemiology_surveillance.html>

[Languages: English, French]

[Public Health Agency of Canada](http://www.phac-aspc.gc.ca/about_apropos/index-eng.php) (PHAC).

- - - - Public Health Infobase

<http://infobase.phac-aspc.gc.ca/index-en.html>

*Archived material.*

- - - - Notifiable Diseases Online

<http://diseases.canada.ca/notifiable/>

*Click on “List of notifiable diseases” or “Notifiable disease charts” to get Canadian statistics on tracked (notifiable) diseases.*

- - - - Reports & Publications

<https://www.canada.ca/en/public-health/services/reports-publications.html>

*Browse alphabetical listing of PHAC publications or use Ctrl-F and search by keyword.*

- - - - Surveillance

<https://www.canada.ca/en/public-health/services/surveillance.html>

*Browse to see if a surveillance program on topic exists. The following sources are duplicates: "Canada Communicable Disease Report (CCDR) weekly" is indexed in PubMed, and both "Public Health Infobase" and "Notifiable Diseases Online" are searched separately in this checklist.*

[Languages: English, French]

[Statistics Canada](http://www.statcan.gc.ca/eng/about/about).

- - - - Canadian Cancer Registry (CCR)

<http://www23.statcan.gc.ca/imdb/p2SV.pl?Function=getSurvey&Id=440378>

*For older data, click on "Other reference periods" tab. For ‘Summary tables’ and ‘Publications’, click on "Related products" tab.*

- - - - Diseases and Physical Health Conditions

<https://www.statcan.gc.ca/eng/subjects/health/diseases_and_health_conditions>

*For keyword search, use search box in the left-hand side or filter results by other categories such as “Type”, “Geography” or “Survey or statistical program”.*

[Languages: English, French]

## US

[Centers for Disease Control and Prevention](http://www.cdc.gov/about/) (CDC)

- - - - FastStats A to Z

<http://www.cdc.gov/nchs/fastats/>

- - - - Morbidity and Mortality Weekly Report (MMWR)

<http://www.cdc.gov/mmwr/>

*Also indexed in PubMed.*

- - - - National Center for Health Statistics

<http://www.cdc.gov/nchs/>

[Languages: English and various others]

[National Cancer Institute](http://seer.cancer.gov/about/). Surveillance, Epidemiology, and End Results Program (SEER)

<http://seer.cancer.gov/>

[Languages: English]

## International

[Organisation for Economic Co-operation and Development](http://www.oecd.org/about/) (OECD). OECD Data

<https://data.oecd.org/>
*Search or browse for appropriate health statistics.*

[Languages: English, French, Dutch, Italian, Japanese, Russian]

[World Health Organization](http://www.who.int/about/en/) (WHO). Global Health Observatory (GHO)

<http://www.who.int/gho/en/>

*Find health data on 193 WHO members by browsing the navigational links. Supersedes WHO Statistical Information System (WHOSIS).*

[Languages: English, French, Spanish, Russian, and various others]

# Internet Search

## Search Engines

*For most topics a comprehensive Internet search is impossible. For systematic searches we recommend reviewing the top 50 to 100 hits in a search engine such as Google.*

[Google](http://www.google.ca/intl/en/about.html)

[http://www.google.com](http://www.google.com/)

*If no results, try another search engine.*

[Language: English]

[Google Scholar](http://scholar.google.com/intl/en/scholar/about.html).

<http://scholar.google.ca/schhp?hl=en&as_sdt=0,5>

[Language: English]

[Canadian Medical Association](https://www.cma.ca/about-cma) (CMA). Affiliated, Associated Societies and Observer Organizations

<https://www.cma.ca/affiliates-associated-societies-and-observer-organizations>

*Links to Canadian professional associations that may be of interest or can use search engine to locate others if needed.*

[Language: English]

Professional Associations/Manufacturers' Sites (if applicable)

## Open Access Journals

[Lund University Libraries](https://doaj.org/about). Directory of Open Access Journals (DOAJ)

<http://www.doaj.org/>

*Click on "Advanced Search" to search by specific fields such as title or abstract; can also browse by subject, language, etc.*

[Language: English]

# Miscellaneous

## Behavioural Change

[Canadian Agency for Drugs and Technologies in Health](https://www.cadth.ca/rx-change) (CADTH). Rx for Change

<https://www.cadth.ca/resources/rx-for-change/database/browse>

[Language: English]

## Natural Medicine and Environmental Health

[United States Environmental Protection Agency](http://hero.epa.gov/index.cfm/content/home) (EPA). Health & Environmental Research Online (HERO)

<http://hero.epa.gov/index.cfm/search/main>

[Language: English]

[Alternative Medicine Foundation](http://www.amfoundation.org/about.htm). HerbMed®

<http://www.herbmed.org/>

*Subscription required for full access.*

[Language: English]

[Memorial Sloan-Kettering Cancer Center](https://www.mskcc.org/about). About Herbs

<https://www.mskcc.org/cancer-care/treatments/symptom-management/integrative-medicine/herbs/search>

[Language: English]

#

## Dentistry

[Aetna Inc.](https://www.aetna.com/about-us.html) Aetna Dental Clinical Policy Bulletins

<https://www.aetna.com/health-care-professionals/clinical-policy-bulletins/dental-clinical-policy-bulletins.html>

[Language: English]

[ADA. Center for Evidence-Based Dentistry](http://ebd.ada.org/en/about/). Evidence Database

<http://ebd.ada.org/en/evidence/evidence-by-topic>

*Choose a topic to see systematic reviews, guidelines, or summaries.*

[Language: English]

## Diagnostic Tests

[American Association for Clinical Chemistry](https://www.aacc.org/about-aacc) (AACC). Practice Guidelines

<https://www.aacc.org/science-and-research/practice-guidelines>

[Language: English]

[Centers for Disease Control and Prevention](http://www.cdc.gov/about/) (CDC). Public Health Genomics Knowledge Base. Guideline Database

<https://phgkb.cdc.gov/PHGKB/evidencerStartPage.action>

[Language: English]

[Ontario Association of Medical Laboratories](https://oaml.com/about-oaml/) (OAML). Guidelines

<https://oaml.com/guidelines/>

*Lab test guidelines; those marked with an asterisk are also in the CMA Infobase guideline repository (see Clinical Practice Guidelines section of this checklist).*

[Language: English]

## Mental Health

[Substance Abuse & Mental Services Administration](https://www.samhsa.gov/). Evidence-Based Practices Resource Center (NREPP)

<https://www.samhsa.gov/ebp-resource-center>

*Online database of mental health and substance abuse interventions/programs that have been reviewed and rated.*

[Language: English]

## Nursing

[Canadian Nurses Association](https://www.cna-aiic.ca/en/about-cna) (CNA). Download/Buy

<https://www.cna-aiic.ca/en/download-buy>

*Search or browse Download/Buy section on the left-hand side.* [Languages: English, French]

[Registered Nurses' Association of Ontario](http://rnao.ca/about) (RNAO). Publications & Resources

<http://rnao.ca/resources>

*Includes best practice guidelines, position statements, reports, and other publications. Use the search bar in the middle of the page to search within publications.*

[Language: English]

## Physiotherapy/Rehabilitation

[Centre of Evidence-Based Physiotherapy](http://www.pedro.org.au/english/about-us/cebp/) (CEBP). Physiotherapy Evidence Database: PEDro

<http://www.pedro.org.au/>

*Click "Begin an advanced search" to find information.*

[Languages: English, French, Chinese, German, Spanish, Portuguese, Italian, Japanese, Korean, Turkish, Tamil]

[National Rehabilitation Information Center](http://www.naric.com/?q=en/about) (NARIC). REHABDATA database

<http://www.naric.com/?q=en/SearchRehabdata>

[Language: English]

[University of Ottawa. School of Rehabilitation Sciences](http://www.health.uottawa.ca/rehabguidelines/en/login.php). Evidence-based Practice

<http://www.health.uottawa.ca/rehabguidelines/en/search.php>

[Languages: English, French]

[University of Queensland](http://www.otseeker.com/About/Default.aspx). OTseeker

<http://www.otseeker.com/>

*Contains abstracts of systematic reviews and randomized controlled trials relevant to occupational therapy. Trials have been critically appraised and rated for validity and interpretability.*

[Language: English]
